# Electrostatic Work Causes Unexpected Reactivity in Ionic Photoredox Catalysts in Low Dielectric Constant Solvents

**DOI:** 10.1021/acs.jpcb.5c01038

**Published:** 2025-04-04

**Authors:** Justin L. Ratkovec, Justin D. Earley, Max Kudisch, William P. Kopcha, Eve Yuanwei Xu, Robert R. Knowles, Garry Rumbles, Obadiah G. Reid

**Affiliations:** † Department of Chemistry, University of Colorado Boulder, Colorado 80309, United States; ‡ National Renewable Energy Laboratory, Golden, Colorado 80309, United States; § Department of Chemistry, Princeton University, Princeton, New Jersey 08544, United States; ∥ Renewable and Sustainable Energy Institute, University of Colorado Boulder, Boulder, Colorado 80309, United States

## Abstract

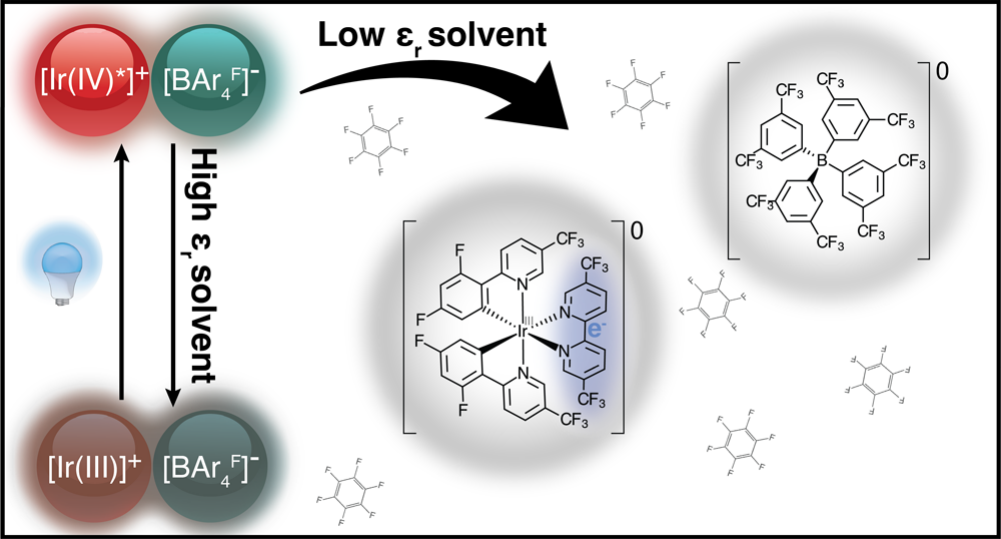

We show that in low dielectric constant (ε_*r*_) solvents, the prototypical cationic photoredox catalyst [Ir(III)(dFCF_3_ppy)_2_-(5,5′-dCF_3_bpy)]^+^ is capable of oxidizing its counterion in an unexpected photoinduced electron transfer (PET) process. Photoinduced oxidation of the tetrakis[3,5-bis(trifluoromethyl)phenyl]borate (abbv. [BAr_4_^F^]^−^) anion leads to its irreversible decomposition and a buildup of the neutral Ir(III)(dFCF_3_ppy)_3_-(5,5′-dCF_3_ bpy^·–^) (abbv. [Ir(dCF_3_^·-^)]^0^) species. The rate constant of the PET reaction, *k*_*rxn*_, between the two oppositely charged ions was determined by monitoring the growth of absorption features associated with the singly reduced product molecule, [Ir(dCF_3_^·–^)]^0^, in various solvents with a range of ε_*r*_. The PET reaction between the ions of [Ir(dCF_3_) – BAr_4_^F^] is predicted to be nonspontaneous (Δ*G*_PET_ ≥ 0) in high ε_*r*_ solvents, such as acetonitrile, and we observe that *k*_*rxn*_ ≃ 0 under these circumstances. However, *k*_*rxn*_ increases as ε_*r*_ decreases. We attribute this change in spontaneity to the electrostatic work described by the Born (Δ*G*_*S*_) and Coulomb () correction terms to the change in Gibbs free energy of a PET (Δ*G*_PET_). The electrostatic work associated with these often-neglected corrections can be utilized to design novel and surprising photoredox chemistry. Our facile preparation of [Ir(dCF_3_^·–^)]^0^ is one example of a general rule: ion-paired reactants can result in energetic neutral products that chemically store photon energy without an associated Coulomb binding between them.

## Introduction

Photoredox catalysis is a light-induced approach to drive chemical reactions, often unattainable through traditional thermal means. By precisely transferring excited-state energy through oxidation or reduction of a substrate, photoredox catalysts enable a wide array of chemical transformations.^1−6^

Herein, we focus on how ion pairing occurs between the prototypical cationic photoredox catalyst, [5,5′-Bis(trifluoromethyl)-2,2′-bipyridine-N1,N1′]bis[3,5-difluoro-2-[5-(trifluoromethyl)-2-pyridinyl-N]phenyl-C]Iridium(III) (abbv. [Ir(dCF_3_)]^+^), and a counterion can cause unexpected reactivity in low dielectric constant (ε_*r*_) solvents. Specifically, we examine photoinduced oxidation of the common counterion [BAr_4_^F^]^−^. Although this phenomenon is fully predicted by long-established physics, we find that the relevant corrections for the calculation of the Gibbs free energy change have fallen into disuse. It is our purpose here to demonstrate their vitality and utility for understanding and controlling photoredox reactivity in lower ε_*r*_ solvents.

We chose [Ir(dCF_3_)]^+^ and the two common counterions [PF_6_]^−^ and [BAr_4_^F^]^−^ as our model system because similar homoleptic and heteroleptic iridium-based complexes have been at the center of photoredox catalysis. They possess vast electronic and structural tunability,^7^ enabling them to meet the conditions required for a wide range of chemical reactions. Moreover, a growing use case for these iridium complexes is in low ε_*r*_ solvent environments, where the correction terms we have discussed above become extremely important. This is in addition to other interesting effects of ion-pair association in photoredox catalysis that have so far focused on how the counterion affects the photophysics of a chromophore,^8−11^ how Coulombically binding molecules can enhance energy transfer rates,^12,13^ and how the steric hindrance presented by the bound counterion influences photocatalytic reactivity.^14−16^

**Figure 1:**
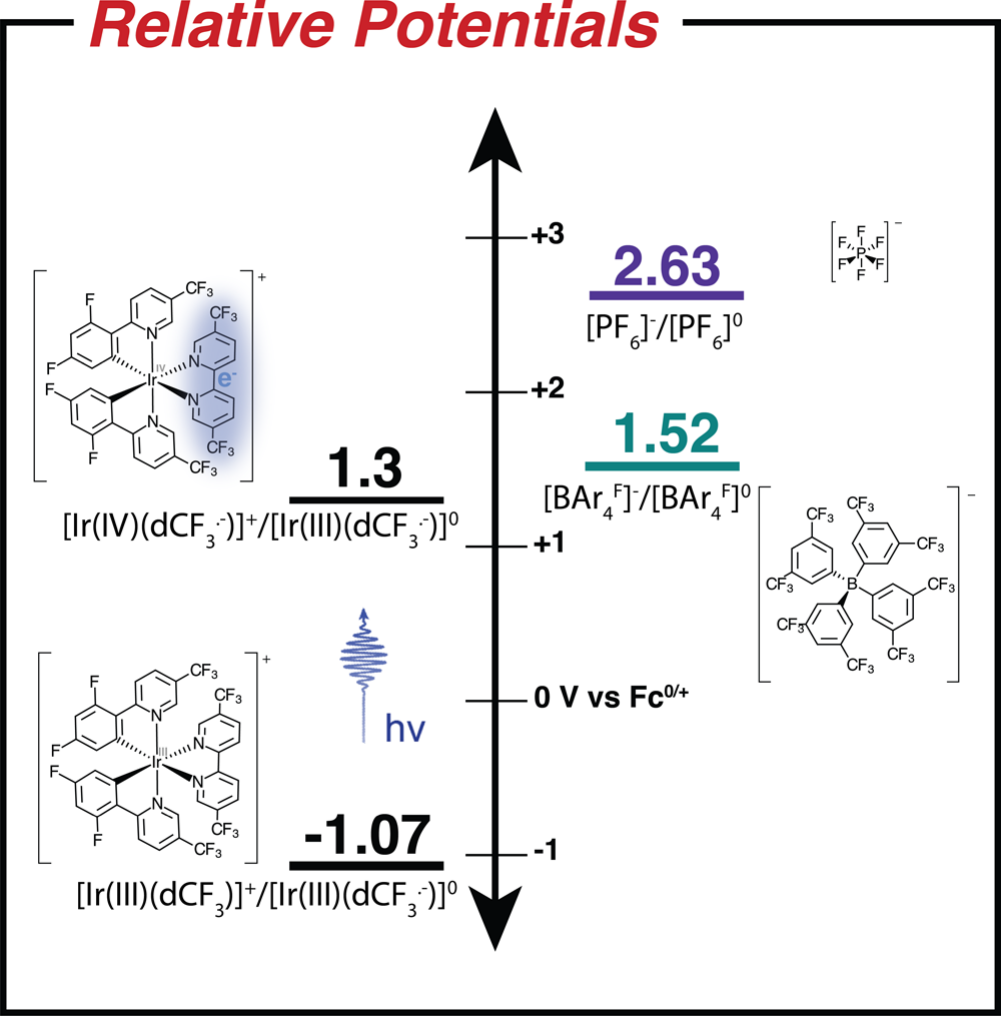
Reduction potentials of [Ir(dCF_3_)]^+^ in its ground and excited state and the oxidation potentials of [BAr_4_^F^]^−^ and [PF_6_]^−^.^17−19,24^ All potentials are measurements in acetonitrile relative to Fc^0/+^.

The photoinduced electron transfer (PET) we observe between [Ir(dCF_3_)]^+^ and tetrakis[3,5-bis(trifluoromethyl)phenyl]borate (abbv. [BAr_4_^F^]^−^) is surprising because of the large oxidation potential involved: 1.56 V vs Fc^0/+^ and 2.63 V vs Fc^0/+^ measured in acetonitrile for [BAr_4_^F^]^−^ and [PF_6_]^−^,^17−19^ respectively (see Figure 1). Using these numbers uncorrected suggests that the Gibbs free energy change for electron transfer to the excited state of [Ir(dCF_3_)]^+^ should be Δ*G* = +0.22 and +1.326 eV, respectively.^20^ The key point is that this calculation holds true only in the solvent wherein the redox potentials were measured. This observation is commonplace for studies of electron transfer that produce an ion pair from neutral molecules, where the driving force and rate of electron transfer both decline as the solvent ε_*r*_ is reduced. The fact that the opposite trend occurs for reactants that begin as an ion pair and produce neutral molecules is much less widely appreciated. As we describe in detail below and show experimentally, this phenomenon is well described by the combined application of the Born and electrostatic work corrections to calculate the Gibbs free energy change for electron transfer. The former correction accounts for an expected shift in the redox potential of any molecule as a function of the solvent ε_r_, while the latter describes the electrostatic work that is either expended or extracted during a PET event.

Notably, measuring redox potentials in a low ε_*r*_ environment is quite difficult due to the low solubility of supporting electrolytes,^21,22^ and these electrolytes themselves influence the solvent ε_*r*_.^23^ In the present case, the low solubility of [Na-BAr_4_^F^] in low ε_*r*_ solvents, the tendency of [BAr_4_^F^]^−^ to degrade upon oxidation, and the oxidation potential of [PF_6_]^−^ being outside of most solvent windows make cyclic voltammetry in the solvents of interest here futile.

In what follows, we use a combination of UV–vis absorption spectroscopy, electron paramagnetic resonance (EPR), and nuclear magnetic resonance (NMR) to study the irreversible PET reaction between [Ir(dCF_3_)]^+^ and as a function of solvent ε_*r*_, which becomes exergonic for all ε_*r*_ ≤ 4. Our experiments are uniquely facilitated in this model system by the known molar absorptivity of the product molecule in a low ε_*r*_ solvent (benzene ε_*r*_ = 2.28) with an easily distinguishable absorption spectrum, the product persisting on the time scale of hours, and the good solubility of [Ir(dCF_3_) – BAr_4_^F^] in some low ε_*r*_ solvents. We also show through these experiments that PET within [Ir(dCF_3_) – PF_6_] remains endergonic throughout the window of ε_*r*_ measured, making it a useful control.

These experiments highlight the importance of both counterion and solvent choice in determining the reactivity of the system and suggest innovative concepts for storing photon energy in the neutral products of PET between charged species.

## Results and Discussion

**Figure 2:**
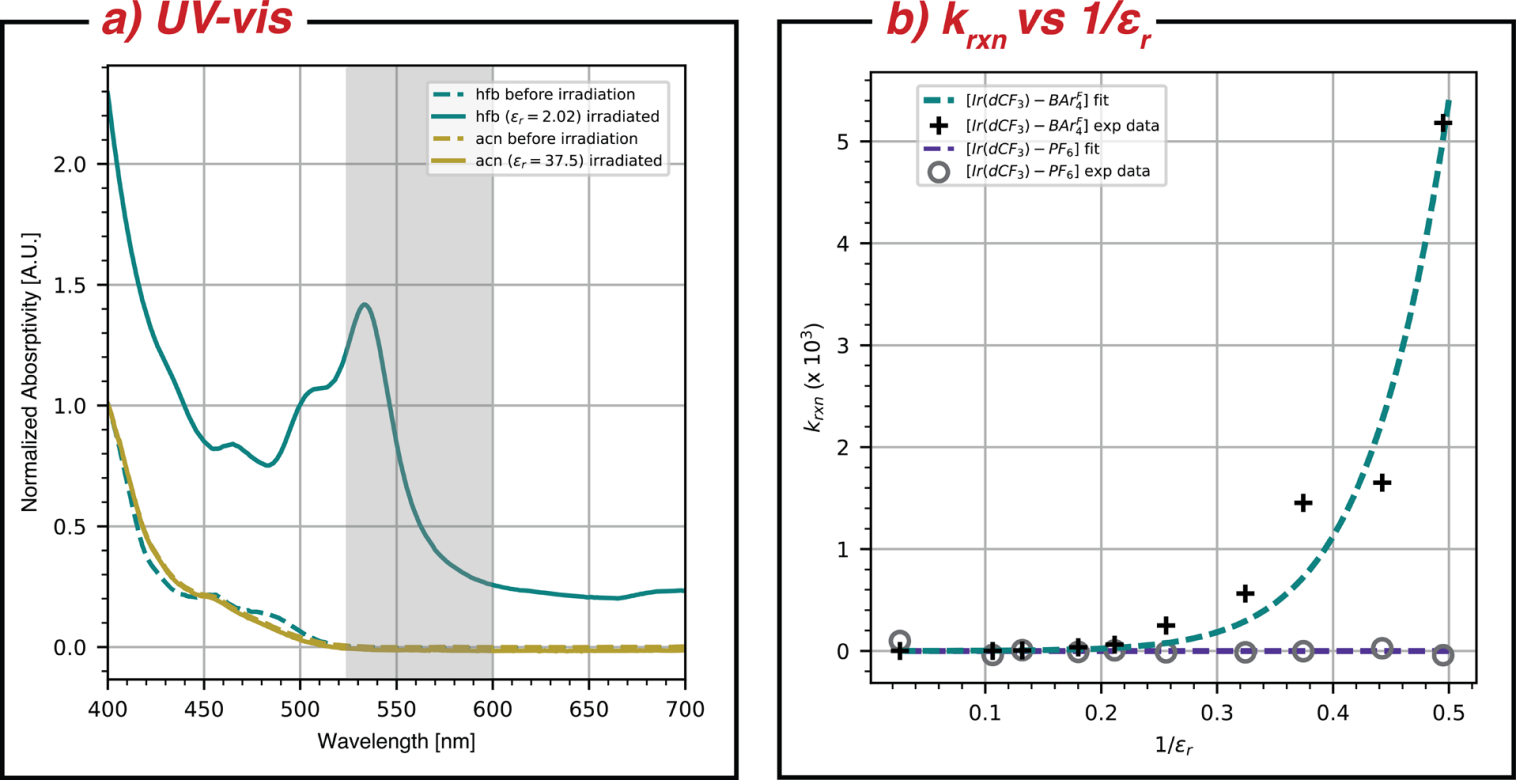
(a) UV/vis absorption spectra of [Ir(dCF_3_)]^+^ and [Ir(dCF_3_^·–^)]^0^ taken in hexafluorbenzene (teal) and acetonitrile (gold) before (dashed) and after (solid) illumination. The small differences in absorption spectra of [Ir(dCF_3_)]^+^ in either solvent prior to irradiation show negligible solvatochromatic shifts. The shaded region shows the spectral range that was integrated to quantify the progress of the reaction. (b) Measured rate constant, *k*_*rxn*_, as a function of the solvent dielectric constant, ε_*r*_, for both [Ir(dCF_3_) – BAr_4_^F^] (black+) and [Ir(dCF_3_) – PF_6_] (gray o). The semiclassical Marcus equation was fitted to both data sets (dashed lines).

Figure 2a,b summarizes the major results of this work. Panel (a) shows a comparison of absorption spectra before and after continuous wave illumination with 470 nm light (60 min with 30 mW) for [Ir(dCF_3_) – BAr_4_^F^] in acetonitrile (gold) and hexafluorobenzene (teal). No photoinduced changes appear in acetonitrile; however, in hexafluorobenzene, the diagnostic spectrum of Ir(III)(dFCF_3_ppy)_2_-(5,5′-dCF_3_bpy^·–^) (abbv. [Ir(dCF_3_^·–^)]^0^) at ≃ 530 nm^25^ is dominant after illumination. The absence of significant solvatochromic shifts in the absorption spectra of [Ir(dCF_3_)]^+^ prior to irradiation shows that there is no substantive change in the excited state energy that could account for this behavior. The growth of the absorption band shaded in gray is entirely due to production of [Ir(dCF_3_^·–^)]^0^ as discussed below, and we use its integral combined with the known extinction coefficient^25^ assumed to be invariant with the solvent to calculate the reaction rate constants (*k*_*rxn*_) shown in Figure 2b. Here, we plot *k*_*rxn*_ as a function of ε_*r*_ for both [BAr_4_^F^]^−^ and [PF_6_]^−^ anions, showing that the rate constant for photoinduced production of [Ir(dCF_3_^·–^)]^0^ increases continuously and dramatically as the dielectric constant of the solvent drops below 3 for [BAr_4_^F^]^−^, but not for [PF_6_]^−^, consistent with the greater oxidation potential of the latter. Evidently, when ε_*r*_ is low enough, the excited state of [Ir(dCF_3_) – BAr_4_^F^] is capable of oxidizing the [BAr_4_^F^]^−^ counterion to produce [Ir(dCF_3_^·–^)]^0^. The remainder of this paper is devoted to a detailed characterization of the reaction products and explaining how this unexpected reaction is possible.

Isolation of reduced heteroleptic Ir complexes has previously been achieved, resulting in a series of neutral iridium-based complexes, including [Ir(dCF_3_^·–^)]^0^.^25^ These were prepared by chemically reducing the cationic iridium complex with KC_8_ in a thawing benzene solution, where the extra electron density localized on the bipyridine ligand drastically changed the features observed by UV/vis absorption, EPR, and NMR spectroscopy.

One of the most prominent changes was the growth of several features in the UV/vis absorption spectra, where the [Ir(dCF_3_^·–^)]^0^ produced by chemical reduction displayed UV/vis spectral features with two vibronic progressions, with little overlap with [Ir(dCF_3_)]^+^, centered at 530 and 850 nm. The 530 nm band is used in Figure 1 to track the photoinduced production of [Ir(dCF_3_^·–^)]^0^.

We do not observe photoinduced generation of [Ir(dCF_3_^·–^)]^0^ in any solvent with ε_*r*_ > 9, excluding two solvents (pyridine and DMF) having either oxidation potentials that are sufficiently low to be oxidized by a photoexcited [Ir(dCF_3_)]^+^, a more stabilized ion-pair product state compared to [Ir(dCF_3_) – PF_6_], enabling electrostatic work-assisted PET, or the presence of impurities (see the SI: Oxidizing Solvents for more details). The dependence of this transformation on ε_*r*_ is discussed below.

Two additional spectroscopic tools, EPR and NMR, were used to confirm our photoinduced production of [Ir(dCF_3_^·–^)]^0^. EPR spectra provide direct evidence for a photoinduced one-electron reduction of [Ir(dCF_3_)]^+^, while NMR reveals that [BAr_4_^F^]^−^ is indeed the electron donor and that oxidation thereof drives its decomposition. The ground state of [Ir(dCF_3_)]^+^ is closed-shell (*S* = 0), whereas the ground state of [Ir(dCF_3_^·–^)]^0^ is open-shell (*S* = 1/2) with a ligand-centered radical localized mainly on the bpy ligand. The ligand-centered reduction of [Ir(dCF_3_)]^+^ results in an axial EPR spectrum at *T* = 10 K that is consistent with reported spectra (see the SI: EPR for more details).^25^ These features appear in our experiments only after irradiation and under low ε_*r*_ conditions already described above, concomitant with the UV/vis band at 530 nm.

^1^H NMR analysis of irradiated [Ir(dCF_3_) – BAr_4_^F^] in benzene shows that the growth of the organic radical and 530 nm absorption features are associated with the degradation of [BAr_4_^F^]^−^. We observe two peaks at δ 7.65 and 7.54 ppm associated with a [BAr_4_^F^]^−^ decrease in intensity and a litany of new peaks in the range of δ 7.3 to 8.6 ppm that grow in only after irradiation and in a low ε_r_ solvent (see the SI: NMR for more details). We propose that photoinduced oxidation of [BAr_4_^F^]^−^ is followed by decomposition, which is what allows [Ir(dCF_3_^·–^)]^0^ to persist in solution. Previous work indicates the degradation of [BAr_4_^F^]^−^ upon oxidation by cyclic voltammetry into a multitude of product molecules.^17^

**Figure 3:**
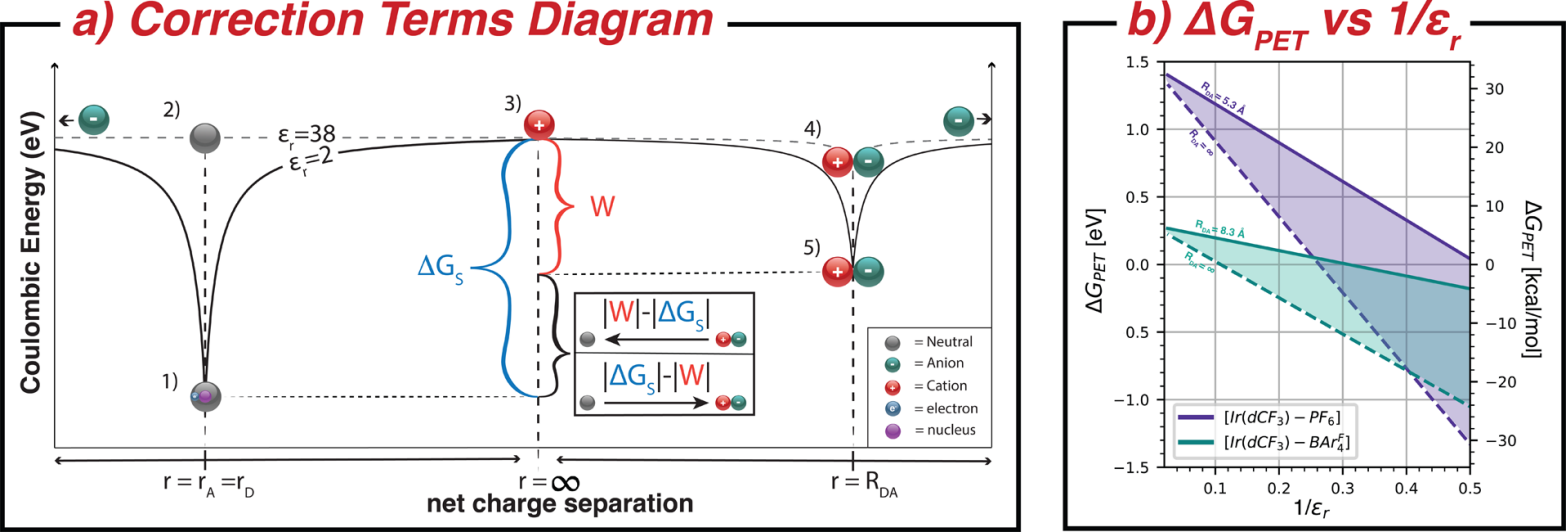
(a) Illustration of the electrostatic work associated with the Born and Coulombic corrections to Δ*G*_PET_. The left-hand side of the diagram shows the contribution from the Born correction as the electrostatic work associated with the creation of a charged species in a dielectric that differs from that of the dielectric in which the redox potentials were measured. At position (1), an electron is paired with a positively charged molecular ion, giving an overall neutral molecule in a solvent dielectric of 2. The electron is moved to an infinite distance (*r* = *∞*) with respect to the starting position and creates a cation at position (3). The difference in the energy required to create a cation in a solvent dielectric of 2 and 38 atoms is given by the Born correction (Δ*G*_*S*_). If the radii of the donor and the acceptor molecule are the same (*r* = *r*_*A*_ = *r*_*D*_), then the magnitude of the Born correction will be the same for both.^35,36^ The right-hand side of the diagram shows the Coulombic correction, where position (5) is a contact ion pair with a center-to-center radius of *R*_*DA*_. The electrostatic work required to separate the two ions to infinite distances and reach position (3) in a solvent dielectric of 2 is the value of . The summation of the two correction terms () becomes significant when an electron transfer occurs in low dielectric media and its sign is dictated by the charge of the reactants and product species.  will be positive when starting with neutral reactants forming charged products and will be negative when starting with charged species forming neutral products. (b) Calculated Δ*G*_PET_ as a function of the dielectric constant of the solvent, ε_*r*_, is depicted and tabulated for [Ir(dCF_3_) – BAr_4_^F^] (teal) and [Ir(dCF_3_) – PF_6_] (purple) using eq 1. For each chemical system, the solid line assumes that the ion pair exists in contact for all values of ε_*r*_ with their *R*_*DA*_ values being 8.3 and 5.3 Å for [Ir(dCF_3_) – BAr_4_^F^] and [Ir(dCF_3_) – PF_6_], respectively. The dashed line for each ion pair is the Δ*G*_PET_ calculated for all ε_*r*_ values for the limit of *R*_*DA*_ →*∞*, or as the contact ion-pair assumption breaks down. The shaded area between the solid and dashed lines is a linear progression of *R*_*DA*_ as it approaches the limit.

As noted in Figure 2a,b, the key variable that enables [BAr_4_^F^]^−^ oxidation and production of [Ir(dCF_3_^·–^)]^0^ is a low ε_*r*_ solvent, which evidently converts this reaction from being endergonic in acetonitrile, as illustrated in Figure 1, to being exergonic in hexafluorobenzene and other low ε_*r*_ solvents. Eq 1 provides a quantitative explanation for how this occurs, combined with the pictorial representation of the electrostatic correction terms in Figure 3. The Gibbs energy change of a photoinduced electron-transfer reaction is given by
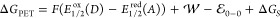
1where F is the Faraday constant,  is the zero-to-zero energy of the principle chromophore excited-state, *E*_1/2_^ox^(*D*) is the standard oxidation potential of the donor, *E*_1/2_^red^(*A*) is the standard reduction potential of the acceptor typically measured in solvents with large values of ε_*r*_ and assumes no change in ε_*r*_ between the measurement and photochemical experiment.^20,23,26^ The measurements of the oxidation and reduction potentials are normally done through cyclic voltammetry, where the electrolyte solution has a large ε_*r*_, and when the PET takes place in a lower dielectric-constant environment, there are two electrostatic correction terms that must be taken into account: the Born correction (Δ*G*_*S*_) and the electrostatic work (). These terms are
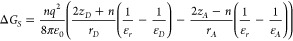
2
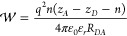
3where *n* is the number of electrons being transferred in the PET event; *r*_*D*_ and *r*_*A*_ are the radii of the donor and acceptor molecules, respectively; *q* is the charge of an electron; ε_0_ is the permittivity of free space; ε_*r*_ is the dielectric constant of the solvent in which the PET occurs; ε_*D*_ and ε_*A*_ are the dielectric constants of the solvent, where the redox potentials of the donor and acceptor molecules were measured, respectively; and *R*_*DA*_ is the distance between the donor and acceptor centers.^27−32^ Values for *r*_*D*_ and *r*_*A*_ were found by estimating the radius of each molecule from the respective optimized geometries. *R*_*DA*_ was determined as the distance between the central atoms of each ion of either ion pair with an optimized geometry. The geometry of each ion pair was determined via DFT calculations (see the SI: Computational Methods for more details).

Both correction terms arise from an electrostatic potential produced from the interaction of two oppositely charged species, and their overall contributions to Δ*G*_PET_ can be understood through Figure 3a. The left-hand side of Figure 3a illustrates the Born correction as the difference in the energy required to separate an electron from a neutral molecule at the radius of the molecule, *r*_*A*_ or *r*_*D*_, to infinite distances in media with ε_*r*_ = 38 vs that with ε_*r*_ = 2. The right-hand side shows the electrostatic work correction, which corresponds to the work required to separate two oppositely charged ions from their minimum center-to-center distance, *R*_*DA*_, to infinity. When the reaction occurs in a high ε_*r*_ solvent, the correction terms have little effect on the overall thermodynamics of the process. However, as ε_*r*_ decreases, the magnitude of the potential wells for both correction terms increases and changes the thermodynamics of a PET that produces either neutral or charged species.

For a PET process within an ion pair as a reactant state yielding neutral products, such as [Ir(dCF_3_) – BArF] shown in Figure 2, the overall contributions from the correction terms are negative and favorable for the forward PET. The overall negative contributions from the correction terms in a low ε_*r*_ solvent are illustrated in Figure 3a. The reactant state is at position (5), where the ion pair exists at a minimum radius, *R*_*DA*_. The electrostatic work extracted by associating the ion pair from infinite distances is quantified by the magnitude of  or the difference in energy between positions (5) and (3) in Figure 3a. This association stabilizes the reactant state, hindering the production of neutral products.

However, the Born correction is larger. The difference in energy between positions (3) and (1) is associated with moving an electron from infinite distances onto the cation to produce a neutral molecule. As a result, the neutral product state experiences a net stabilization relative to the ionic reactants as ε_*r*_ is reduced relative to where the redox potentials were measured, giving a negative overall contribution to Δ*G*_PET_.

This surprising behavior is simply the opposite of a more familiar situation: when neutral reactant molecules undergo PET to become an ion pair. In that case, the overall contributions to Δ*G*_PET_ from these corrections are positive, making the process less favorable in low ε_*r*_ solvents. Previous work by Gould and Farid^33^ has explicitly shown the tendency of Δ*G*_PET_ to become more positive for neutral reactants, producing radical ion pairs in solvents with a range of ε_*r*_ as predicted from the Born and Coulombic correction terms. Additionally, the increase in magnitude of the Coulombic potential created by an ion pair in decreasing ε_*r*_ has been calculated by more sophisticated means.^32,34^

These relatively simple electrostatic corrections quantitatively explain the trends that we observe for the rate constant of the reaction in Figure 2b. There, we measured the rate of photoinduced oxidation between the excited state of [Ir(dCF_3_)]^+^ and its corresponding counteranion, [BAr_4_^F^]^−^ or [PF_6_]^−^, at concentrations of ∼0.05 mM in solvents ranging from ε_*r*_ = 2 to ε_*r*_ = 38.

This experimental data demonstrate that Δ*G*_PET_ is dependent on ε_*r*_ of the solvent and becomes more favorable when the reactants are ions, and the products are neutral molecules. The fit lines in Figure 2b use the Marcus rate equation with Δ*G*_PET_ calculated as a function of dielectric constant according to eq 1; and these values of , Δ*G*_*S*_, and Δ*G*_PET_ are tabulated for both anions (Tables 1 and 2) and illustrated in Figure 3b. In contrast, the calculation of Δ*G*_PET_ without the correction terms predicts no reactivity of [Ir(dCF_3_)]^+^ with either counterion. This change in reactivity demonstrates the profound impact ε_*r*_ has on photoredox reactions and the necessity to include the electrostatic work and the Born correction when calculating Δ*G* for electron transfer processes in low ε_*r*_ environments.

**Table 1:** Summary of the [Ir(dCF_3_) – BAr_4_^F^] Born and Coulombic Correction Terms to the Change in the Gibbs Free Energy in Units of kcal/mol and the Measured Composite Rate Constant for the Formation of [Ir(dCF_3_^·–^)]^0^ in Each Solvent Condition in Units of s^–1^^a^

solvent	ε_*r*_		Δ*G*_*s*_	Δ*G*_PET_	Δ*G*_PET_^Uncorrected^	*k*_*rxn*_
hexafluorobenzene	2.02	19.9	–29.0	–4.04	5.07	5.2 × 10^3^
1,4-difluorobenzene	2.26	17.8	–25.8	–2.90	5.07	1.7 × 10^3^
87.5 (dfb)/12.5 (fbz)	2.67	15.1	–21.6	–1.43	5.07	1.5 × 10^3^
75 (dfb)/25 (fbz)	3.08	13.1	–18.5	–0.341	5.07	5.6 × 10^2^
50 (dfb)/50 (fbz)	3.91	10.3	–14.2	1.15	5.07	2.5 × 10^2^
25 (dfb)/75 (fbz)	4.73	8.50	–11.5	2.11	5.07	6.2 × 10^1^
fluorobenzene	5.55	7.24	–9.52	2.79	5.07	3.5 × 10^1^
tetrahydrofuran	7.58	5.30	–6.55	3.85	5.07	4.8 × 10^0^
α,α,α-trifluorotoluene	9.40	4.40	–4.96	4.29	5.07	–1.4 × 10^1^
acetonitrile	37.5	1.08	0.00	6.13	5.07	–1.1 × 10^1^

a The Gibbs free energy change without corrections (Δ*G*_PET_^Uncorrected^) is also provided. The constants used to calculate the correction terms and Δ*G*_PET_ as a function of ε_*r*_ are listed as follows: *F* = 96485.3321 C · mol^–1^, *E*_1/2_^ox^(*D*) = +1.52 V vs Fc^0/+^, *E*_1/2_^red^(*A*) = – 1.07 V vs Fc^0/+^, , *q* = 1.602176487 × 10^–^^19^ C, *n* = 1, ε_0_ = 8.854 × 10^–^^12^ F · m^–1^, *z*_*D*_ = – 1, *z*_*A*_ = +1, *r*_*D*_ = 5.58 × 10^–^^10^ m, *r*_*A*_ = 5.16 × 10^–^^10^ m, ε_*D*_ = ε_*A*_ = 37.5, *R*_*DA*_ = 8.25 × 10^–^^10^ m*.*

**Table 2:** Summary of the [Ir(dCF_3_) – PF_6_] Born and Coulombic Correction Terms to the Change in the Gibbs Free Energy in Units of kcal/mol and the Measured Composite Rate Constant for the Formation of [Ir(dCF_3_^·–^)]^0^ in Each Solvent Condition in Units of s^–1^^a^

solvent	ε_*r*_		Δ*G*_*s*_	Δ*G*_PET_	Δ*G*_PET_^Uncorrected^	*k*_*rxn*_
hexafluorobenzene	2.02	31.3	–60.6	1.29	30.5	4.2 × 10^1^
1,4-difluorobenzene	2.26	28.0	–53.8	4.75	30.5	2.6 × 10^1^
87.5 (dfb)/12.5 (fbz)	2.67	23.7	–45.0	9.24	30.5	–2.9 × 10^0^
75 (dfb)/25 (fbz)	3.08	20.5	–38.6	12.5	30.5	–1.4 × 10^1^
50 (dfb)/50 (fbz)	3.91	16.2	–29.7	17.1	30.5	–1.4 × 10^1^
25 (dfb)/75 (fbz)	4.73	13.4	–23.9	20.0	30.5	5.9 × 10^0^
fluorobenzene	5.55	11.4	–19.9	22.1	30.5	–9.3 × 10^0^
tetrahydrofuran	7.58	8.35	–13.7	25.3	30.5	7.9 × 10^0^
α,α,α-trifluorotoluene	9.40	6.73	–10.3	27.0	30.5	–4.0 × 10^1^
acetonitrile	37.5	1.68	0.00	32.2	30.5	9.7 × 10^1^

a The Gibbs free energy change without corrections (Δ*G*_PET_^Uncorrected^) is also provided. The constants used to calculate the correction terms and Δ*G*_PET_ as a function of ε_*r*_ are listed as follows: *F* = 96485.3321 C · mol^–1^, *E*_1/2_^ox^(*D*) = +2.626 V vs Fc^0/+^, *E*_1/2_^red^(*A*) = – 1.07 V vs Fc^0/+^, , *q* = 1.602176487 × 10^–^^19^ C, *n* = 1, ε_0_ = 8.854 × 10^–^^12^ F · m^–1^, *z*_*D*_ = – 1, *z*_*A*_ = +1, *r*_*D*_ = 1.71 × 10^–^^10^ m, *r*_*A*_ = 5.16 × 10^–^^10^ m, ε_*D*_ = ε_*A*_ = 37.5, *R*_*DA*_ = 5.25 × 10^–^^10^ m.

## Conclusions

Chemists are accustomed to thinking of the anion as an inert spectator in photoredox reactions using cationic chromophores. This work shows that this is emphatically not true in all instances and that simply changing solvents could lead to unexpected electron transfer reactions within ionic photocatalysts. This surprising behavior is explained by the favorable energetic contributions of the Born and Coulombic corrections to a PET process when the reactants are ionically associated in a low ε_*r*_ solvent and the products are neutral species. In this specific case, we show that the magnitude of these corrections is sufficient to change the endergonic oxidation of [BAr_4_^F^]^−^ by an excited-state [Ir(dCF_3_)]^+^ in acetonitrile to an exergonic process in any solvent with an ε_*r*_ of ≤ 4, such as toluene (ε_*r*_ ≈ 3).

These results should serve both as a warning and as inspiration to the photoredox community. Counterions cannot be considered unconditionally stable. However, we also demonstrate a facile route to the preparation of one-electron-reduced iridium chromophores through irreversible oxidation of the anion and show the potential value of PET within an ion pair to store valuable photon energy. Ion-paired reactants eliminate the kinetic diffusion control that hinders other intermolecular electron transfer processes, while the neutrality of the product states could allow them to diffuse apart efficiently, as there is no Coulomb potential left to bind them.

## Supporting Information Available

Experimental methods and data; oxidizing solvent molecules; and tables of the correction term values given in eV (PDF)
