# Supplementary material for: Electrostatic Work Causes Unexpected Reactivity in Ionic Photoredox Catalysts in Low Dielectric Constant Solvents
Source: J Phys Chem B. 2025 Apr 4;129(15):3895–901. doi: 10.1021/acs.jpcb.5c01038 (PMC12010321; doi:10.1021/acs.jpcb.5c01038)
Supplement: Supplementary file 1 — jp5c01038_si_001.pdf [file jp5c01038_si_001.pdf]

Electrostatic Work Causes Unexpected Reactivity in Ionic Photoredox Catalysts in Low Dielectric Constant Solvents: Supporting Information

Justin L. Ratkovec[1,2], Justin D. Earley[1,2], Max Kudisch[1], William P. Kopcha[1], Eve Yuanwei Xu[3], Robert R. Knowles[3], Garry Rumbles[1,2,4], and Obadiah G. Reid[1,4,*]

[1]National Renewable Energy Laboratory, Golden, CO 80401
[2]University of Colorado Boulder, Department of Chemistry, Boulder, CO 80309
[3]Princeton University, Princeton, NJ 08544
[4]University of Colorado Boulder, Renewable and Sustainable Energy Institute, Boulder, CO 80309
[*]Obadiah.Reid@nrel.gov

April 1, 2025

Contents

1 Experimental Methods and Data **S2**
- 1.1 UV/vis Spectroscopy S2
  - 1.1.1 Integrated absorption as a function of irradiation time S23
- 1.2 Electron paramagnetic resonance (EPR) S26
- 1.3 Nuclear Magnetic Resonance (NMR) S31
- 1.4 Time-Resolved Photoluminescence (long time-scales) S33
- 1.5 Time-Resolved Photoluminescence (short time-scales) S34
- 1.6 Computational Methods S35
- 1.7 Dielectric Loss S49
- 1.8 Time-Resolved Dielectric Loss (TRDL) S52
- 1.9 Ultrafast Transient Absorption S55

2 Oxidizing Solvents **S57**

3 Correction term values in eV Tables **S60**

1 Experimental Methods and Data

1.1 UV/vis Spectroscopy

Experimental Setup:

The photoreactor setup used to obtain all of the UV-vis absorption spectra for this work is shown in Figure S1. The illumination source used is a Thorlabs M470L4 - 470 nm, 760 mW (Min) Mounted LED, 1000 mA at a measured power of 0.655 mW mesaured with a Thorlabs Digital Handheld Optical Power and Energy Meter (PM100D paired with S120VC). The whole setup is on a Agilent Cary baseplate such that the setup can be placed in an existing Agilent Cary UV-Vis-NIR spectrophotometer. The Agilent Cary 5000 UV-Vis-NIR spectrophotometer was used to collect all of the UV-Vis spectra and was setup to collect each spectra every 30 seconds for 120 total scans within the wavelength range of 400-800 nm, a scan rate of 1818.182 nm/min, data interval of 1 nm, and an average time of 0.033 s. A total of 3 mL of the prepared solution is placed in a 1 cm pathlength cuvette (Spectrocell Inc. RF-3010-T). The first absorption spectrum is subtracted from each spectrum resulting in the resulting difference spectra.

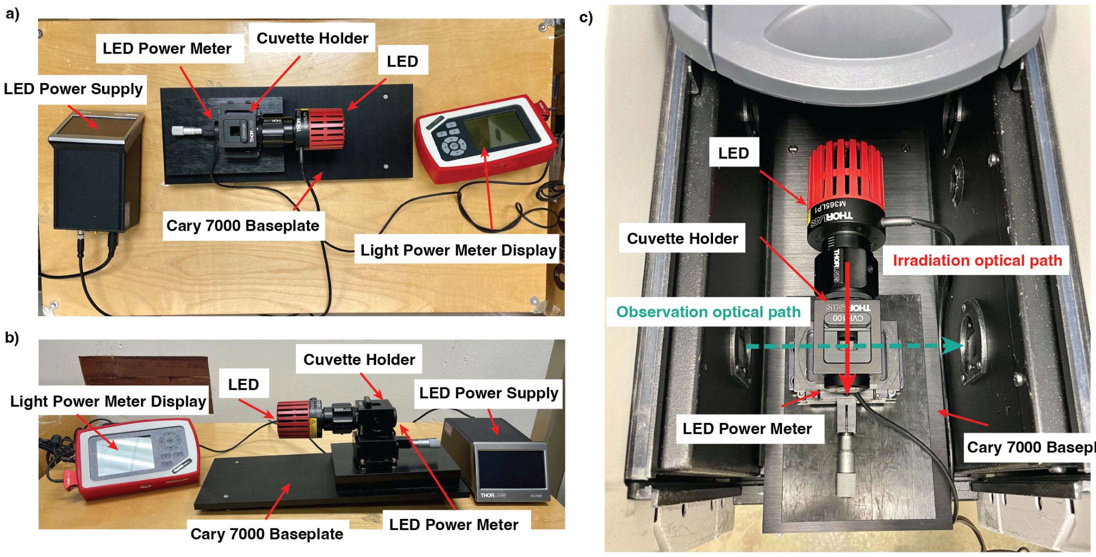

Figure S1: The photoreactor setup used within this work to obtain time-dependent steady-state UV-vis spectra is shown with external power supplies in (a) and (b) and installed in the Agilent Cary 7000 UV-Vis-NIR spectrophotometer.

Hexafluorobenzene:

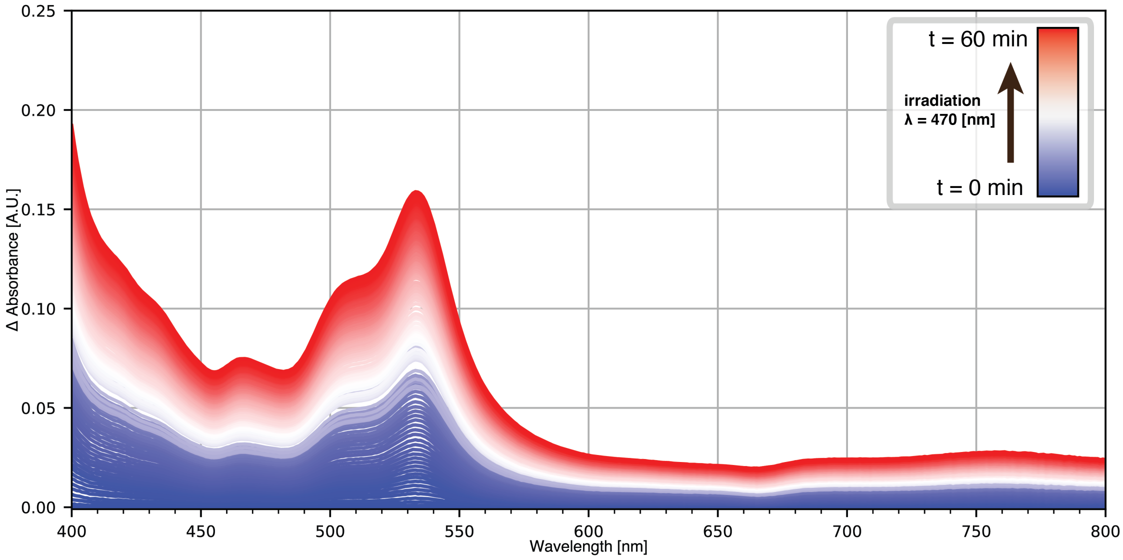

Figure S2: $[Ir(dCF_3) - BAr_4^F]$ dissolved in hexafluorobenzene (hfb) with a concentration of 0.052 mM and irradiated with 0.655 mW of 470 nm light in the photoreactor setup described above. A total of 120 spectra were collected every 30 seconds. The first absorption spectrum is subtracted from each spectrum resulting in the resulting difference spectra.

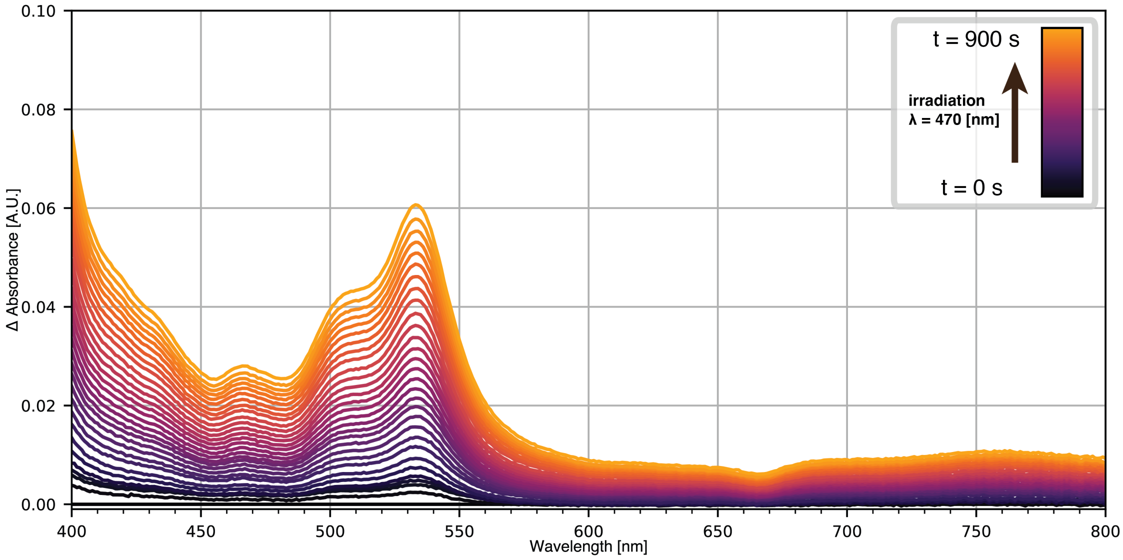

Figure S3: $[Ir(dCF_3) - BAr_4^F]$ dissolved in hexafluorobenzene (hfb) with a concentration of 0.052 mM and irradiated with 0.655 mW of 470 nm light in the photoreactor setup described above. A total of 10 spectra were collected every 30 seconds. The first absorption spectrum is subtracted from each spectrum resulting in the resulting difference spectra.

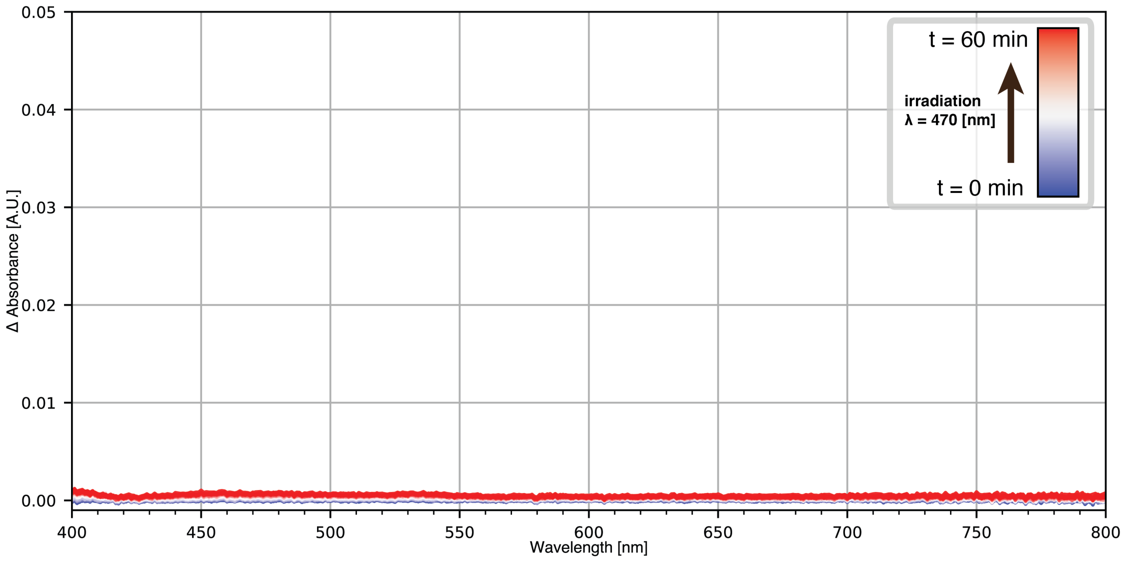

Figure S4: $[Ir(dCF_3) - PF_6]$ dissolved in hexafluorobenzene (hfb) with a concentration of 0.093 mM and irradiated with 0.655 mW of 470 nm light in the photoreactor setup described above. A total of 120 spectra were collected every 30 seconds. The first absorption spectrum is subtracted from each spectrum resulting in the resulting difference spectra.

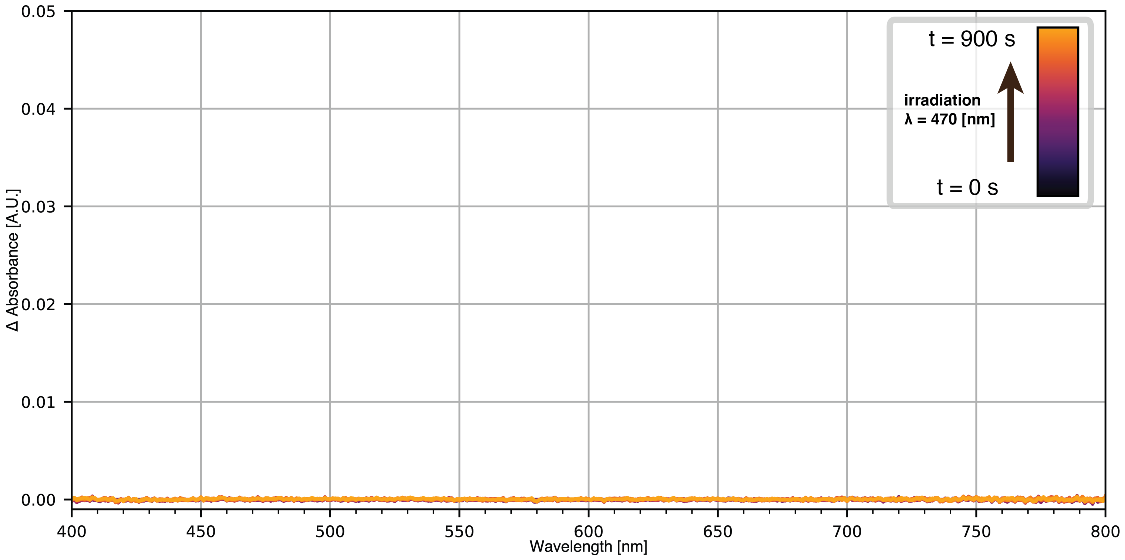

Figure S5: $[Ir(dCF_3) - PF_6]$ dissolved in hexafluorobenzene (hfb) with a concentration of 0.093 mM and irradiated with 0.655 mW of 470 nm light in the photoreactor setup described above. A total of 10 spectra were collected every 30 seconds. The first absorption spectrum is subtracted from each spectrum resulting in the resulting difference spectra.

1,4-Difluorobenzene:

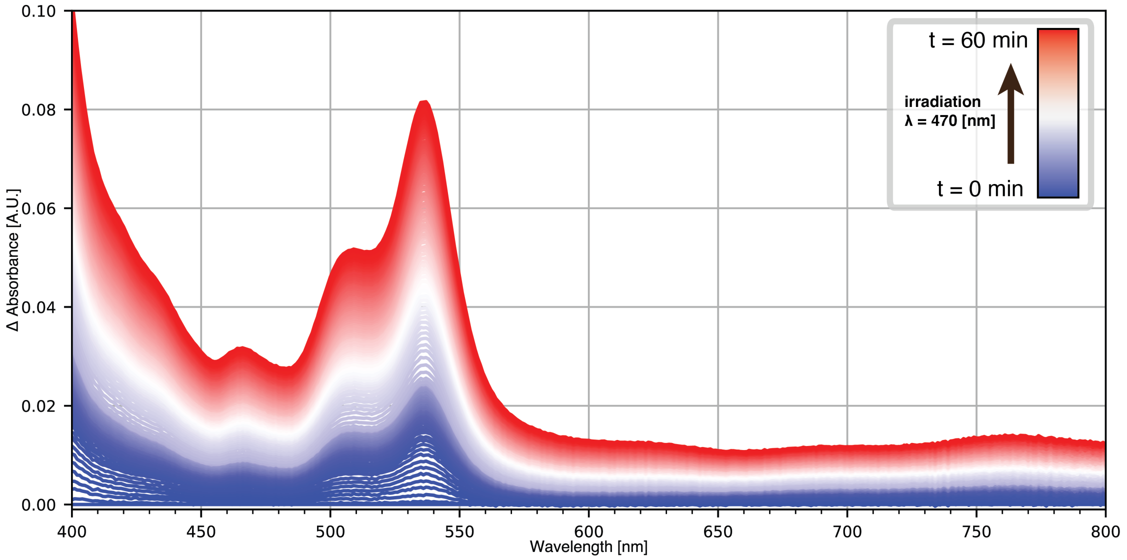

Figure S6: $[Ir(dCF_3){-}BAr_4^F]$ dissolved in 1,4-difluorobenzne (dfb) with a concentration of 0.058 mM and irradiated with 0.655 mW of 470 nm light in the photoreactor setup described above. A total of 120 spectra were collected every 30 seconds. The first absorption spectrum is subtracted from each spectrum resulting in the resulting difference spectra.

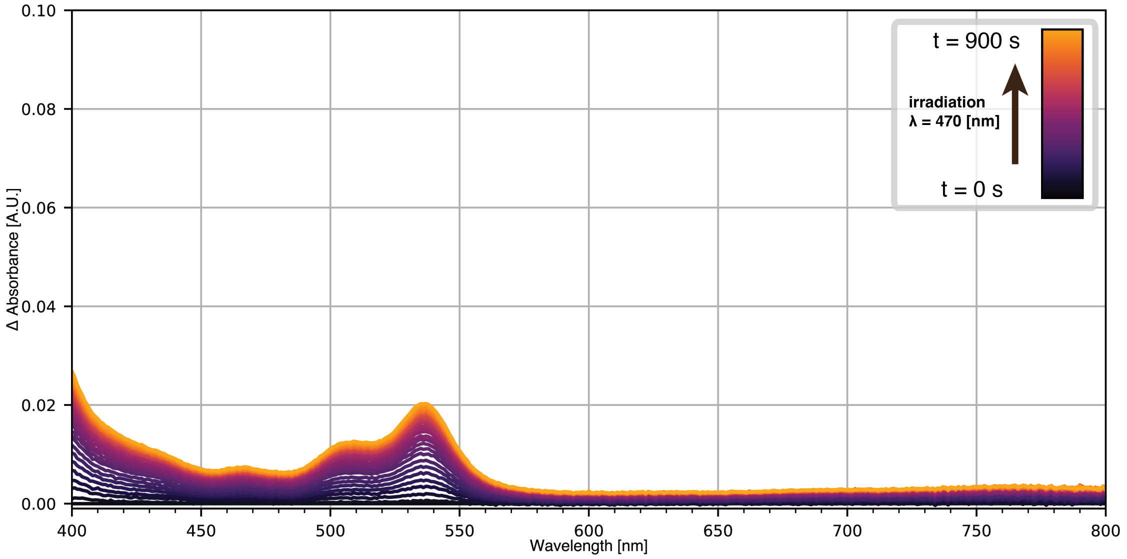

Figure S7: $[Ir(dCF_3){-}BAr_4^F]$ dissolved in 1,4-difluorobenzne (dfb) with a concentration of 0.058 mM and irradiated with 0.655 mW of 470 nm light in the photoreactor setup described above. A total of 10 spectra were collected every 30 seconds. The first absorption spectrum is subtracted from each spectrum resulting in the resulting difference spectra.

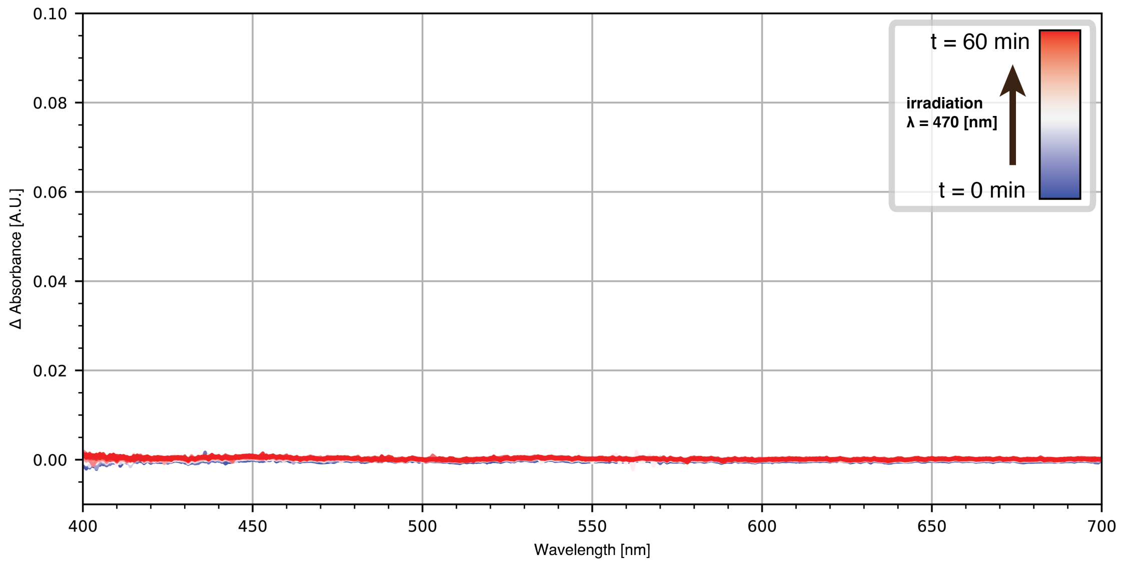

Figure S8: $[Ir(dCF_3) - PF_6]$ dissolved in 1,4-difluorobenzne (dfb) with a concentration of 0.10 mM and irradiated with 0.655 mW of 470 nm light in the photoreactor setup described above. A total of 120 spectra were collected every 30 seconds. The first absorption spectrum is subtracted from each spectrum resulting in the resulting difference spectra.

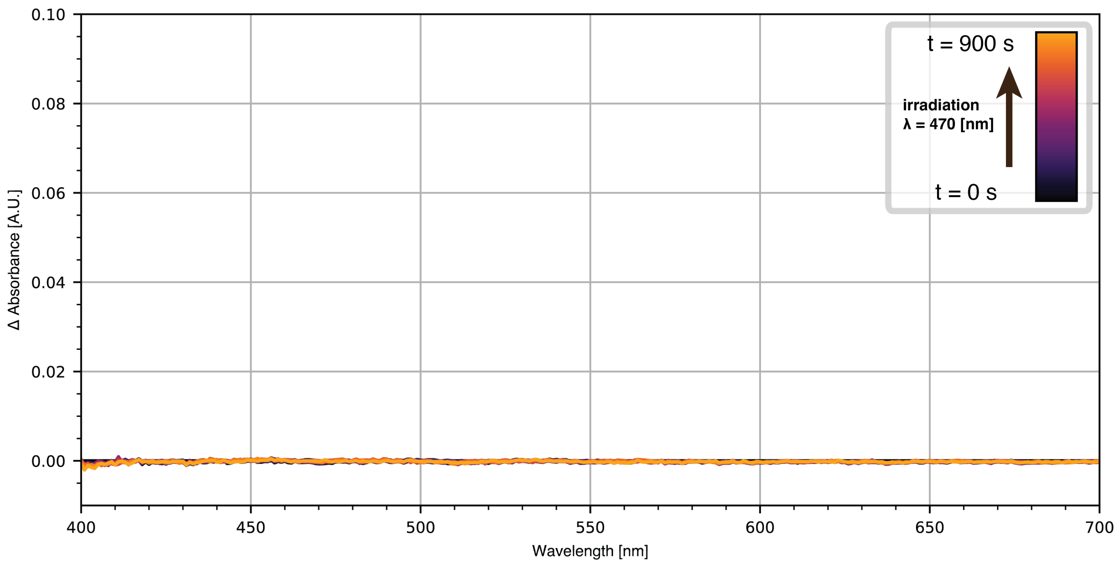

Figure S9: $[Ir(dCF_3) - PF_6]$ dissolved in 1,4-difluorobenzne (dfb) with a concentration of 0.10 mM and irradiated with 0.655 mW of 470 nm light in the photoreactor setup described above. A total of 10 spectra were collected every 30 seconds. The first absorption spectrum is subtracted from each spectrum resulting in the resulting difference spectra.

1,4-Difluorobenzene (87.5) / Fluorobenzene (12.5):

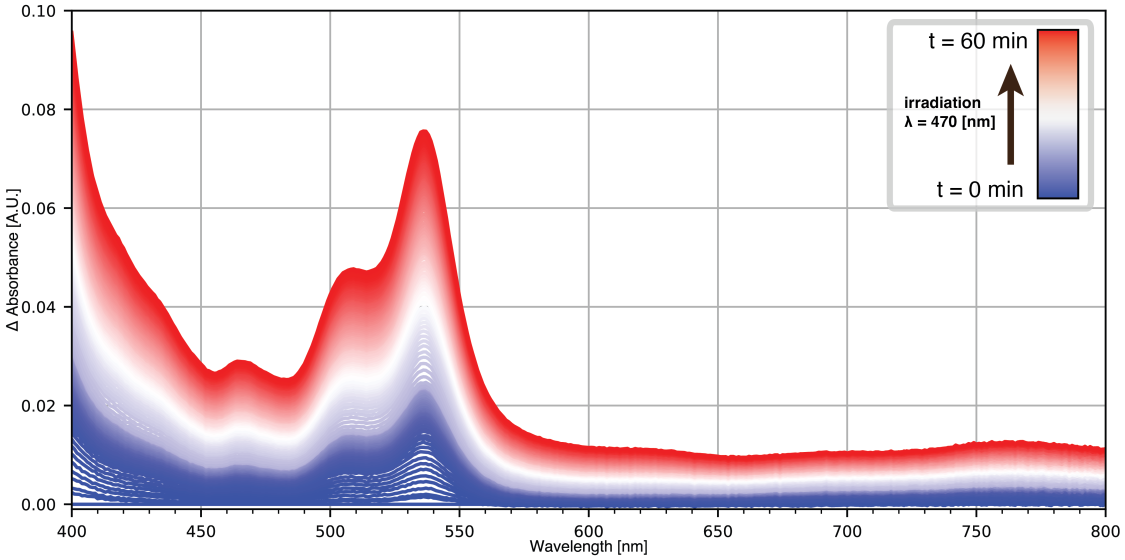

Figure S10: $[Ir(dCF_3) - BAr_4^F]$ dissolved in a mixture of 87.5% 1,4-difluorobenzne (dfb) and 12.5% fluorobenzene by volume with a concentration of 0.051 mM and irradiated with 0.763 mW of 470 nm light in the photoreactor setup described above. A total of 120 spectra were collected every 30 seconds. The first absorption spectrum is subtracted from each spectrum resulting in the resulting difference spectra.

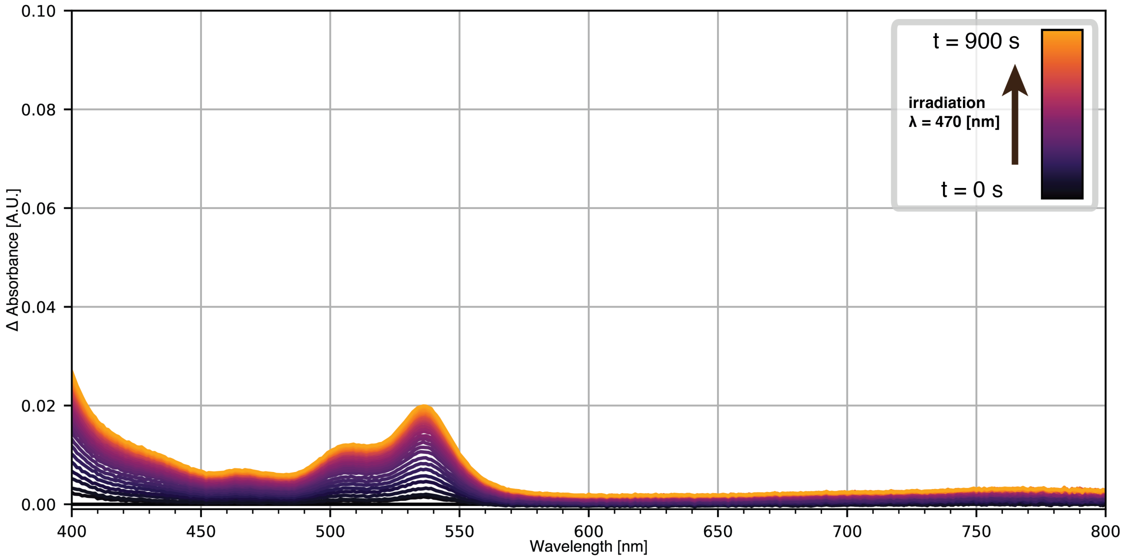

Figure S11: $[Ir(dCF_3) - BAr_4^F]$ dissolved in a mixture of 87.5% 1,4-difluorobenzne (dfb) and 12.5% fluorobenzene by volume with a concentration of 0.051 mM and irradiated with 0.763 mW of 470 nm light in the photoreactor setup described above. A total of 30 spectra were collected every 30 seconds. The first absorption spectrum is subtracted from each spectrum resulting in the resulting difference spectra.

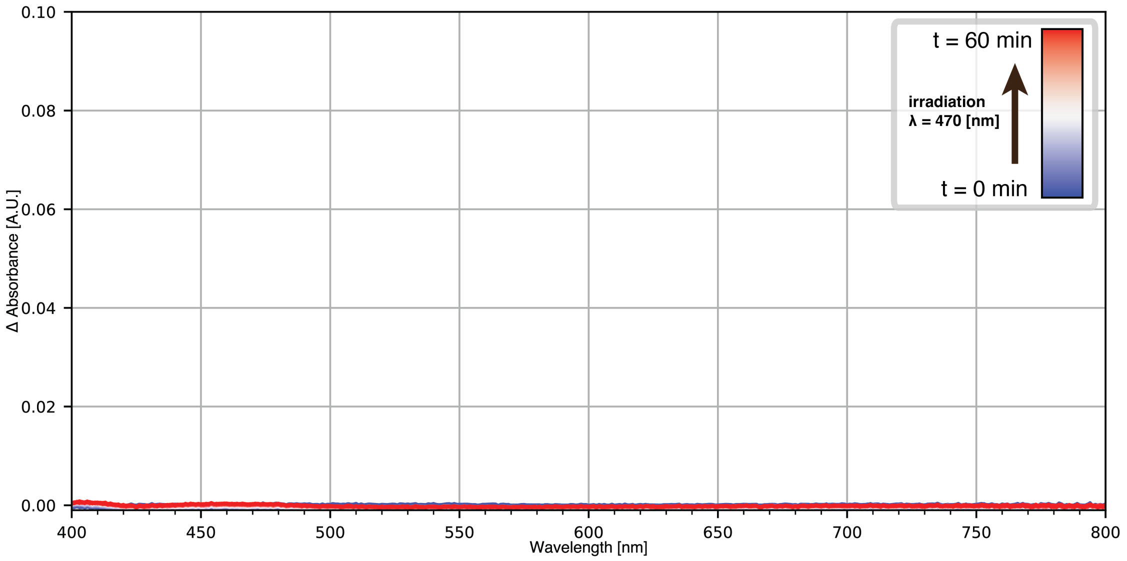

Figure S12: $[Ir(dCF_3) - PF_6]$ dissolved in a mixture of 87.5% 1,4-difluorobenzne (dfb) and 12.5% fluorobenzene by volume with a concentration of 0.073 mM and irradiated with 0.763 mW of 470 nm light in the photoreactor setup described above. A total of 120 spectra were collected every 30 seconds. The first absorption spectrum is subtracted from each spectrum resulting in the resulting difference spectra.

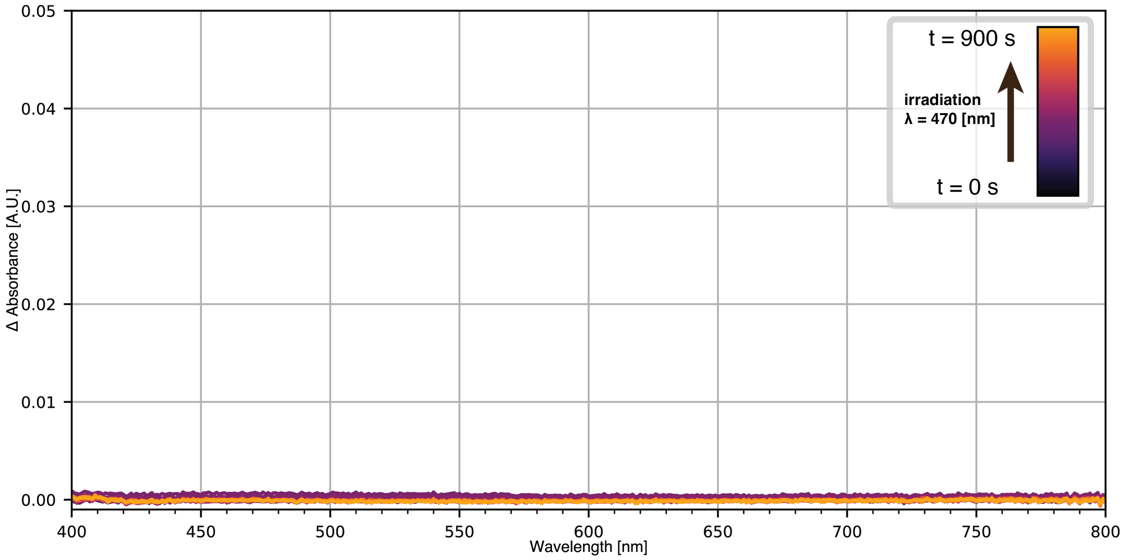

Figure S13: $[Ir(dCF_3) - PF_6]$ dissolved in a mixture of 87.5% 1,4-difluorobenzne (dfb) and 12.5% fluorobenzene by volume with a concentration of 0.073 mM and irradiated with 0.763 mW of 470 nm light in the photoreactor setup described above. A total of 30 spectra were collected every 30 seconds. The first absorption spectrum is subtracted from each spectrum resulting in the resulting difference spectra.

1,4-Difluorobenzene (75) / Fluorobenzene (25):

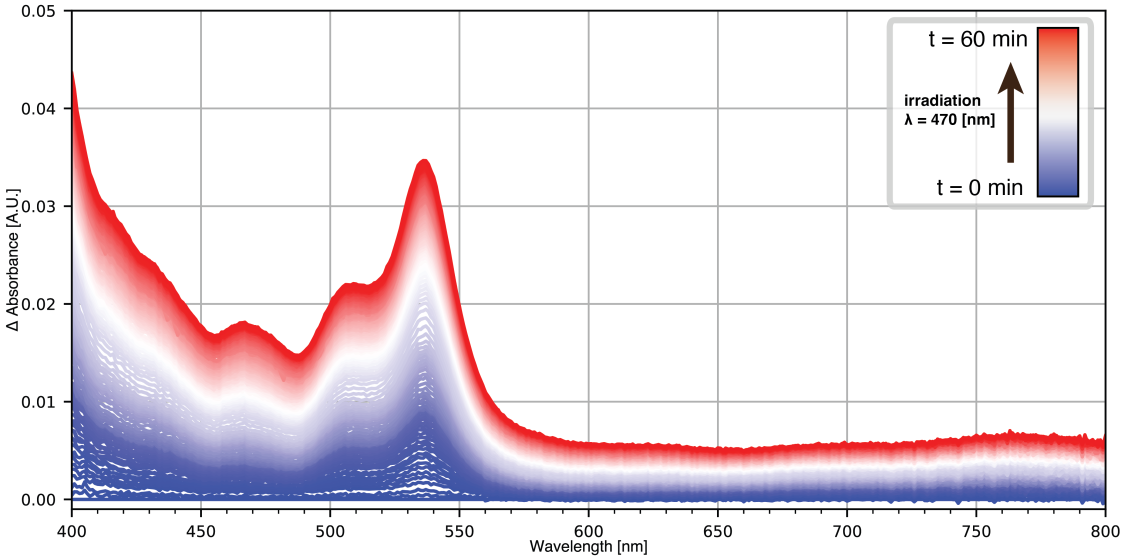

Figure S14: $[Ir(dCF_3) - BAr_4^F]$ dissolved in a mixture of 75% 1,4-difluorobenzne (dfb) and 25% fluorobenzene by volume with a concentration of 0.052 mM and irradiated with 0.763 mW of 470 nm light in the photoreactor setup described above. A total of 120 spectra were collected every 30 seconds. The first absorption spectrum is subtracted from each spectrum resulting in the resulting difference spectra.

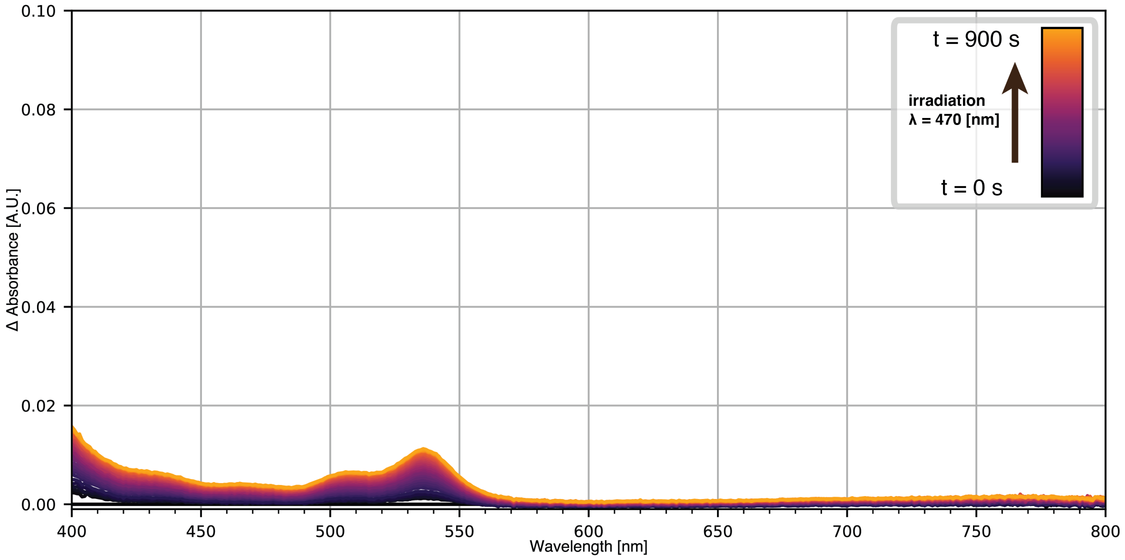

Figure S15: $[Ir(dCF_3) - BAr_4^F]$ dissolved in a mixture of 75% 1,4-difluorobenzne (dfb) and 25% fluorobenzene by volume with a concentration of 0.052 mM and irradiated with 0.763 mW of 470 nm light in the photoreactor setup described above. A total of 30 spectra were collected every 30 seconds. The first absorption spectrum is subtracted from each spectrum resulting in the resulting difference spectra.

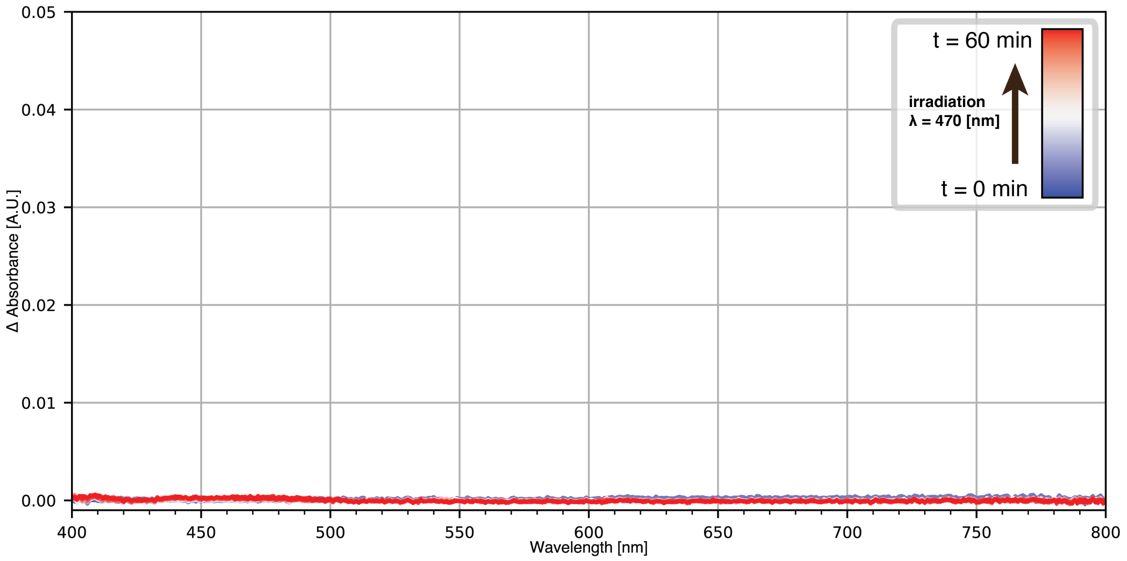

Figure S16: $[Ir(dCF_3)-PF_6]$ dissolved in a mixture of 75% 1,4-difluorobenzne (dfb) and 25% fluorobenzene by volume with a concentration of 0.060 mM and irradiated with 0.763 mW of 470 nm light in the photoreactor setup described above. A total of 120 spectra were collected every 30 seconds. The first absorption spectrum is subtracted from each spectrum resulting in the resulting difference spectra.

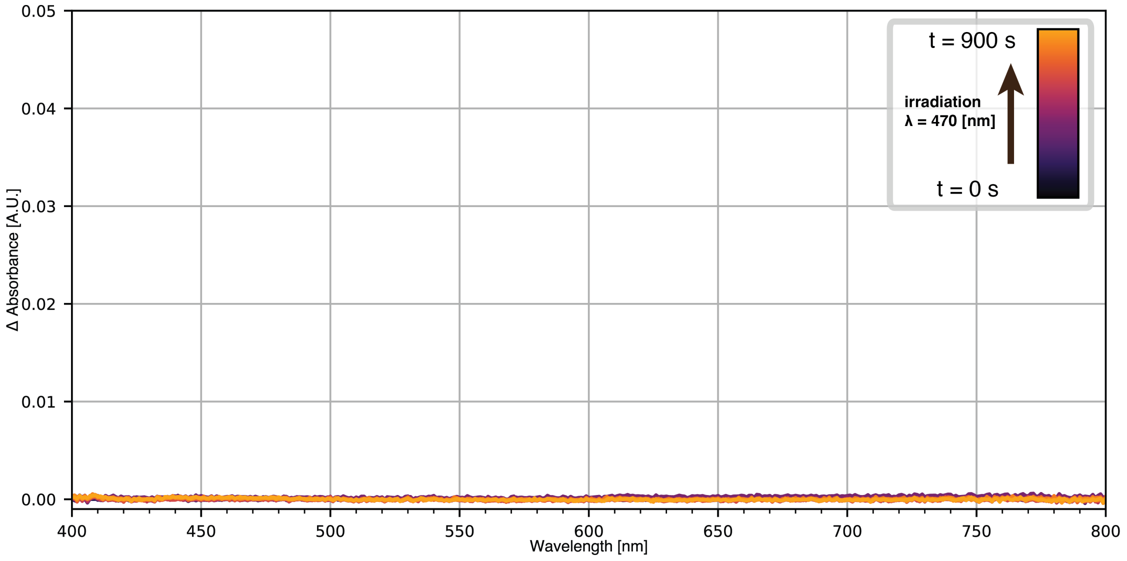

Figure S17: $[Ir(dCF_3)-PF_6]$ dissolved in a mixture of 75% 1,4-difluorobenzne (dfb) and 25% fluorobenzene by volume with a concentration of 0.060 mM and irradiated with 0.763 mW of 470 nm light in the photoreactor setup described above. A total of 30 spectra were collected every 30 seconds. The first absorption spectrum is subtracted from each spectrum resulting in the resulting difference spectra.

1,4-Difluorobenzene (50) / Fluorobenzene (50):

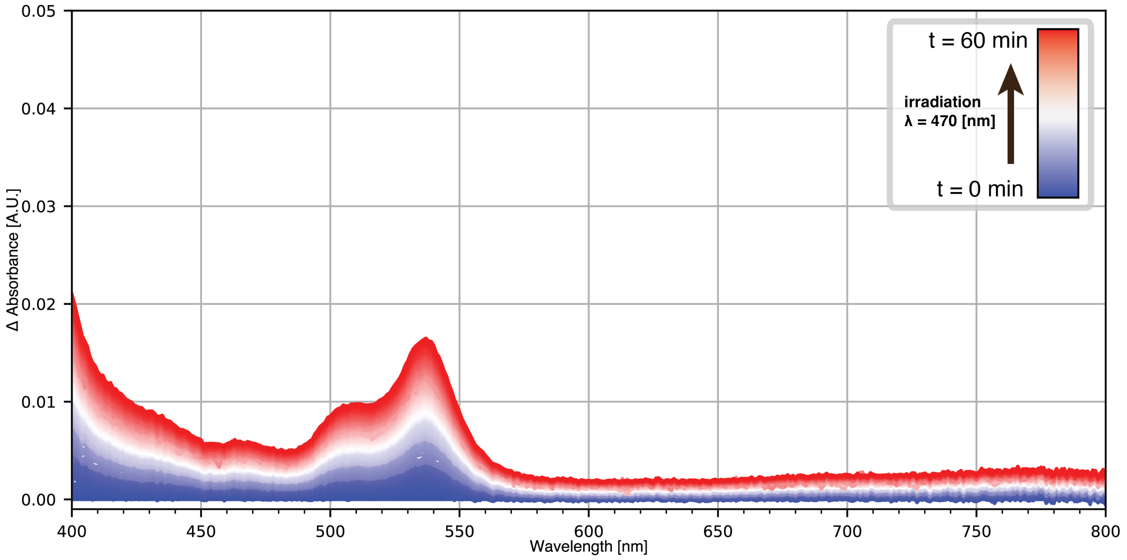

Figure S18: $[Ir(dCF_3) - BAr_4^F]$ dissolved in a mixture of 50% 1,4-difluorobenzne (dfb) and 50% of fluorobenzene by volume with a concentration of 0.048 mM and irradiated with 0.763 mW of 470 nm light in the photoreactor setup described above. A total of 120 spectra were collected every 30 seconds. The first absorption spectrum is subtracted from each spectrum resulting in the resulting difference spectra.

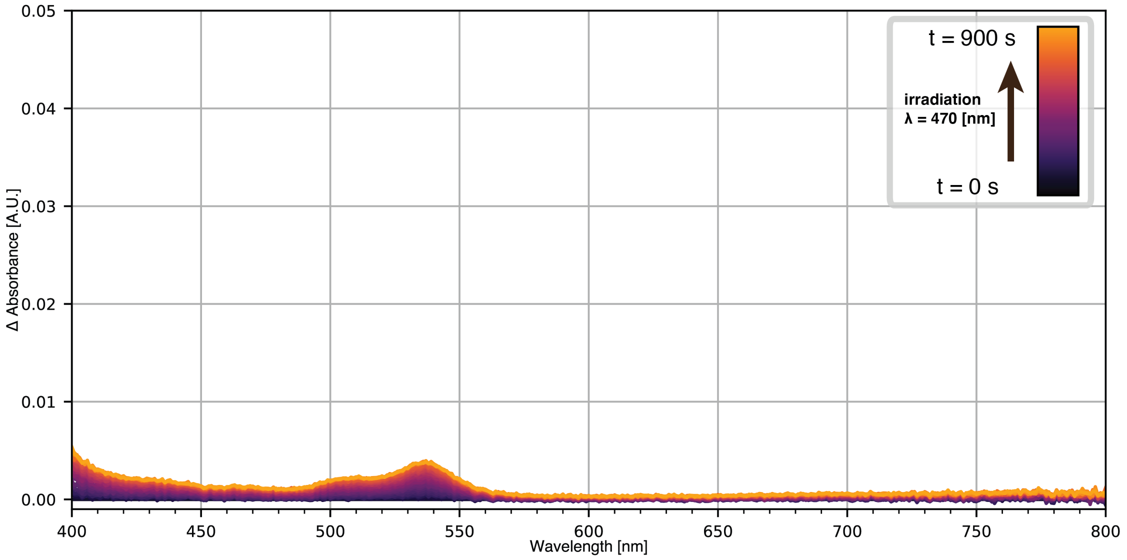

Figure S19: $[Ir(dCF_3) - BAr_4^F]$ dissolved in a mixture of 50% 1,4-difluorobenzne (dfb) and 50% fluorobenzene by volume with a concentration of 0.048 mM and irradiated with 0.763 mW of 470 nm light in the photoreactor setup described above. A total of 30 spectra were collected every 30 seconds. The first absorption spectrum is subtracted from each spectrum resulting in the resulting difference spectra.

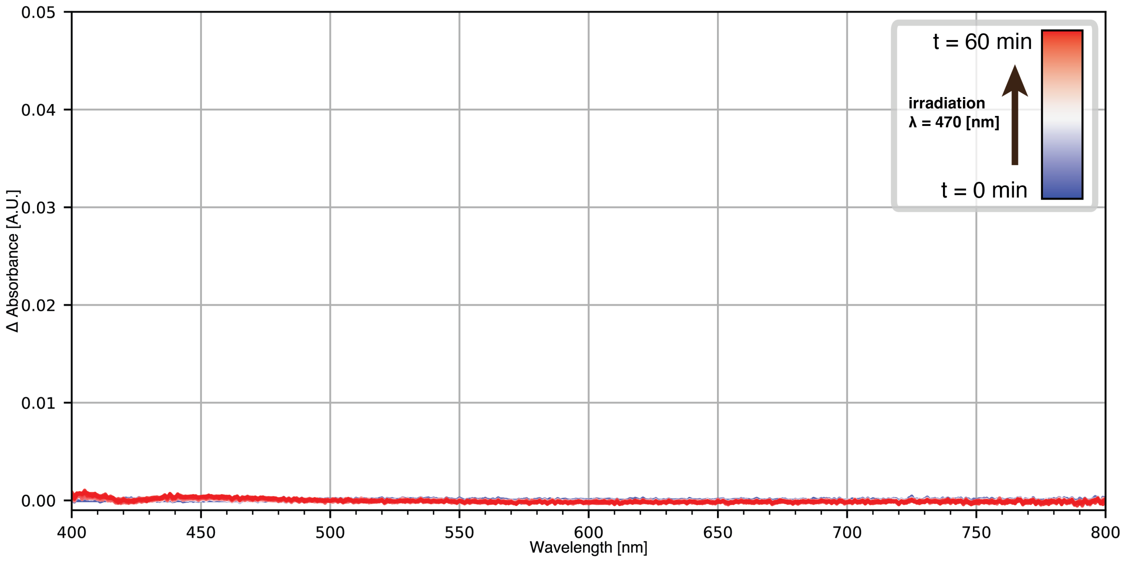

Figure S20: $[Ir(dCF_3) - PF_6]$ dissolved in a mixture of 50% 1,4-difluorobenzne (dfb) and 50% fluorobenzene by volume with a concentration of 0.065 mM and irradiated with 0.763 mW of 470 nm light in the photoreactor setup described above. A total of 120 spectra were collected every 30 seconds. The first absorption spectrum is subtracted from each spectrum resulting in the resulting difference spectra.

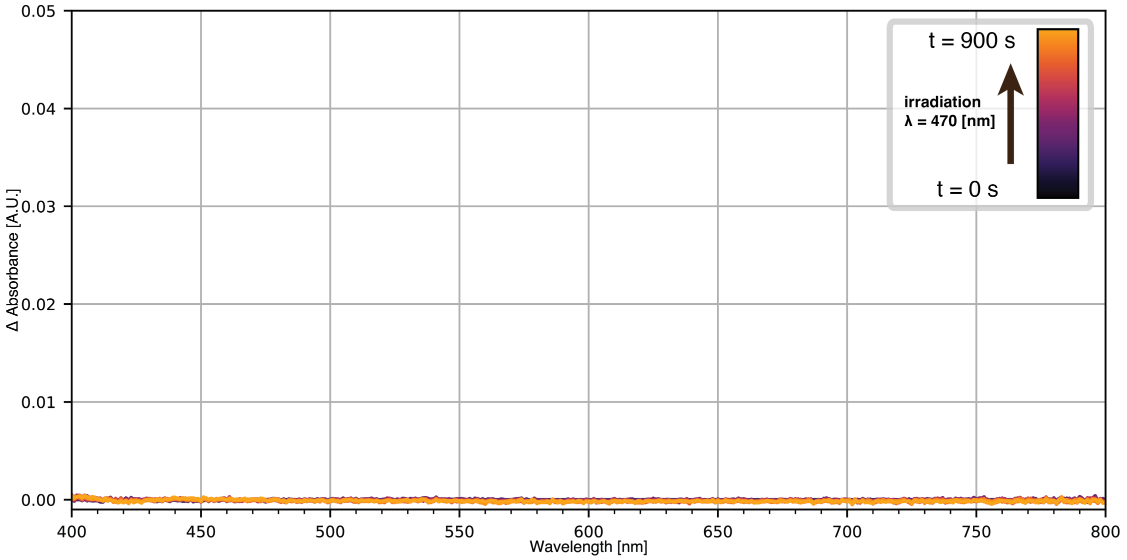

Figure S21: $[Ir(dCF_3) - PF_6]$ dissolved in a mixture of 50% 1,4-difluorobenzne (dfb) and 50% of fluorobenzene by volume with a concentration of 0.065 mM and irradiated with 0.763 mW of 470 nm light in the photoreactor setup described above. A total of 30 spectra were collected every 30 seconds. The first absorption spectrum is subtracted from each spectrum resulting in the resulting difference spectra.

1,4-Difluorobenzene (25) / Fluorobenzene (75):

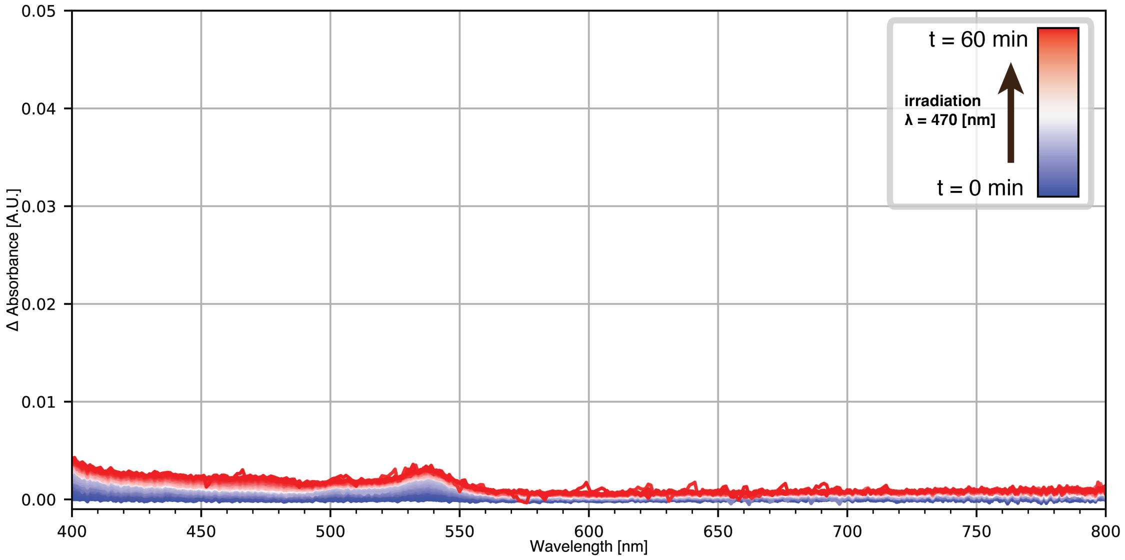

Figure S22: $[Ir(dCF_3) - BAr_4^F]$ dissolved in a mixture of 25% 1,4-difluorobenzne (dfb) and 75% of fluorobenzene by volume with a concentration of 0.063 mM and irradiated with 0.763 mW of 470 nm light in the photoreactor setup described above. A total of 120 spectra were collected every 30 seconds. The first absorption spectrum is subtracted from each spectrum resulting in the resulting difference spectra.

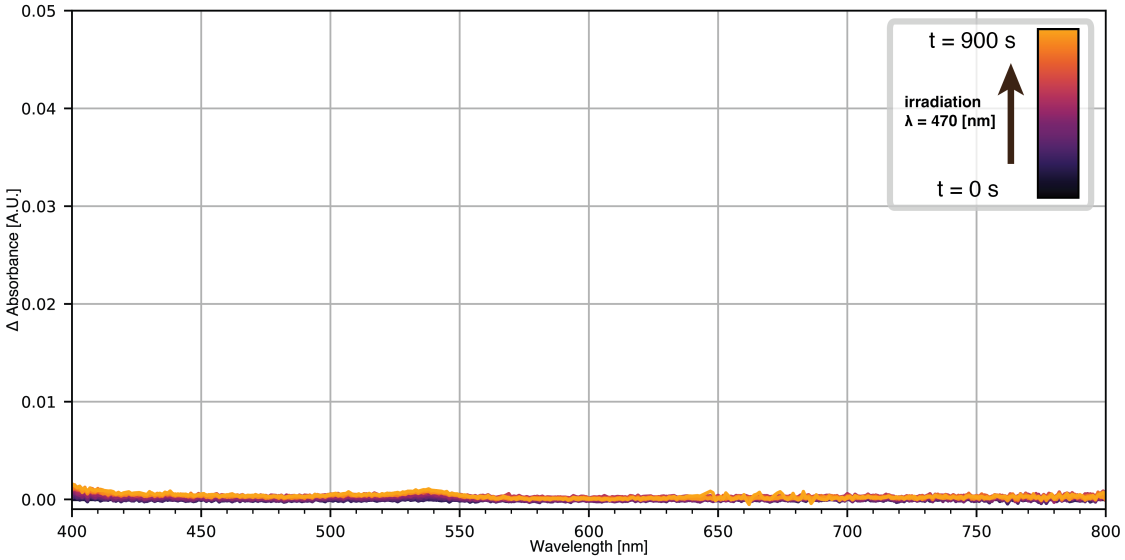

Figure S23: $[Ir(dCF_3) - BAr_4^F]$ dissolved in a mixture of 25% 1,4-difluorobenzne (dfb) and 75% of fluorobenzene by volume with a concentration of 0.063 mM and irradiated with 0.763 mW of 470 nm light in the photoreactor setup described above. A total of 30 spectra were collected every 30 seconds. The first absorption spectrum is subtracted from each spectrum resulting in the resulting difference spectra.

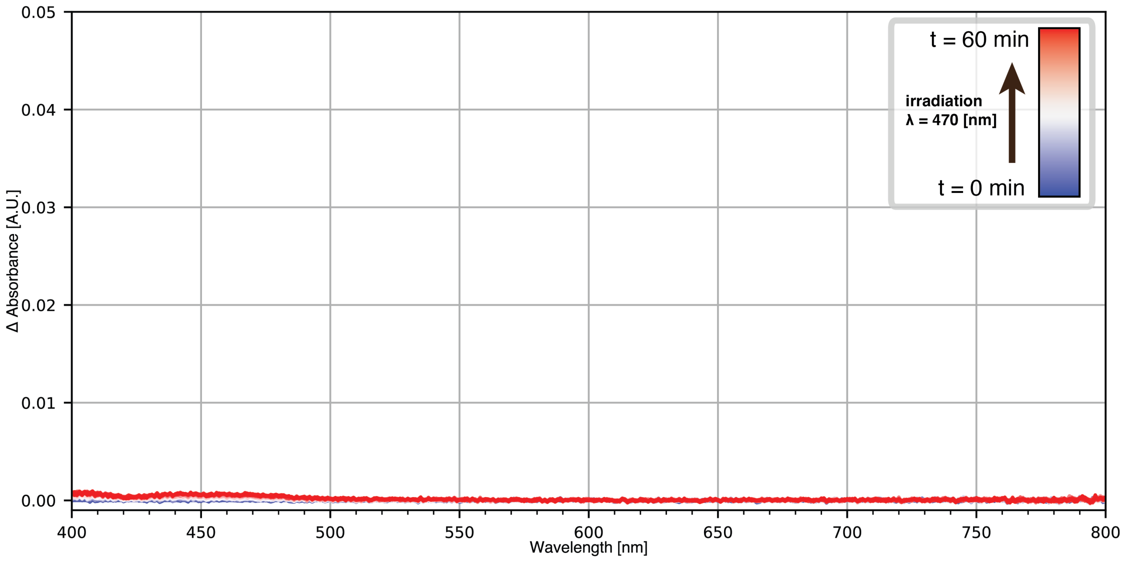

Figure S24: $[Ir(dCF_3) - PF_6]$ dissolved in a mixture of 25% 1,4-difluorobenzne (dfb) and 75% of fluorobenzene by volume with a concentration of 0.083 mM and irradiated with 0.763 mW of 470 nm light in the photoreactor setup described above. A total of 120 spectra were collected every 30 seconds. The first absorption spectrum is subtracted from each spectrum resulting in the resulting difference spectra.

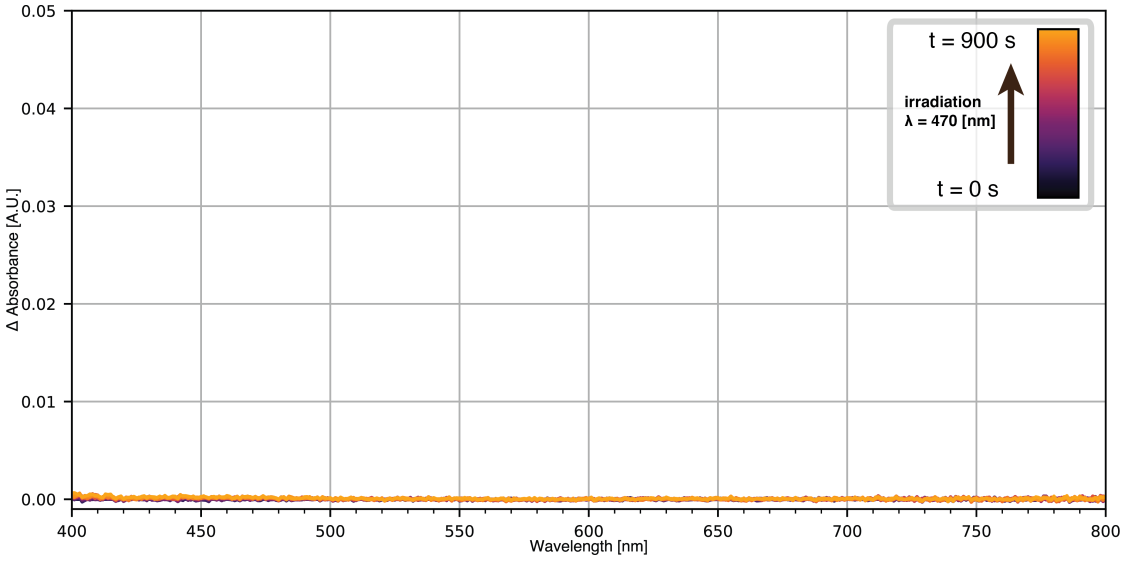

Figure S25: $[Ir(dCF_3) - PF_6]$ dissolved in a mixture of 25% 1,4-difluorobenzne (dfb) and 75% of fluorobenzene by volume with a concentration of 0.083 mM and irradiated with 0.763 mW of 470 nm light in the photoreactor setup described above. A total of 30 spectra were collected every 30 seconds. The first absorption spectrum is subtracted from each spectrum resulting in the resulting difference spectra.

Fluorobenzene:

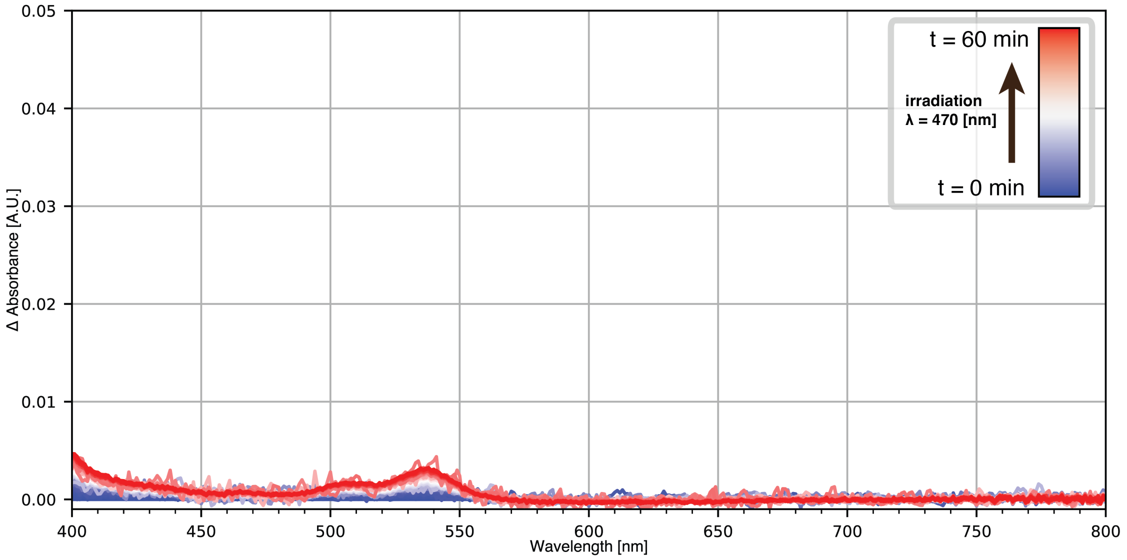

Figure S26: $[Ir(dCF_3){-}BAr_4^F]$ dissolved in fluorobenzene (fbz) with a concentration of 0.063 mM and irradiated with 0.655 mW of 470 nm light in the photoreactor setup described above. A total of 120 spectra were collected every 30 seconds. The first absorption spectrum is subtracted from each spectrum resulting in the resulting difference spectra.

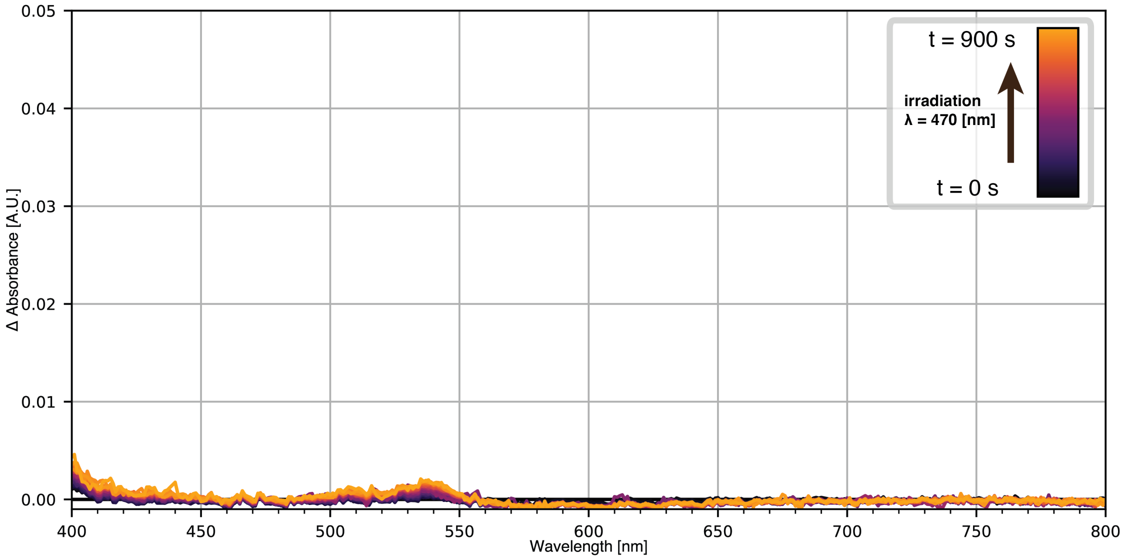

Figure S27: $[Ir(dCF_3){-}BAr_4^F]$ dissolved in fluorobenzene (fbz) with a concentration of 0.063 mM and irradiated with 0.655 mW of 470 nm light in the photoreactor setup described above. A total of 10 spectra were collected every 30 seconds. The first absorption spectrum is subtracted from each spectrum resulting in the resulting difference spectra.

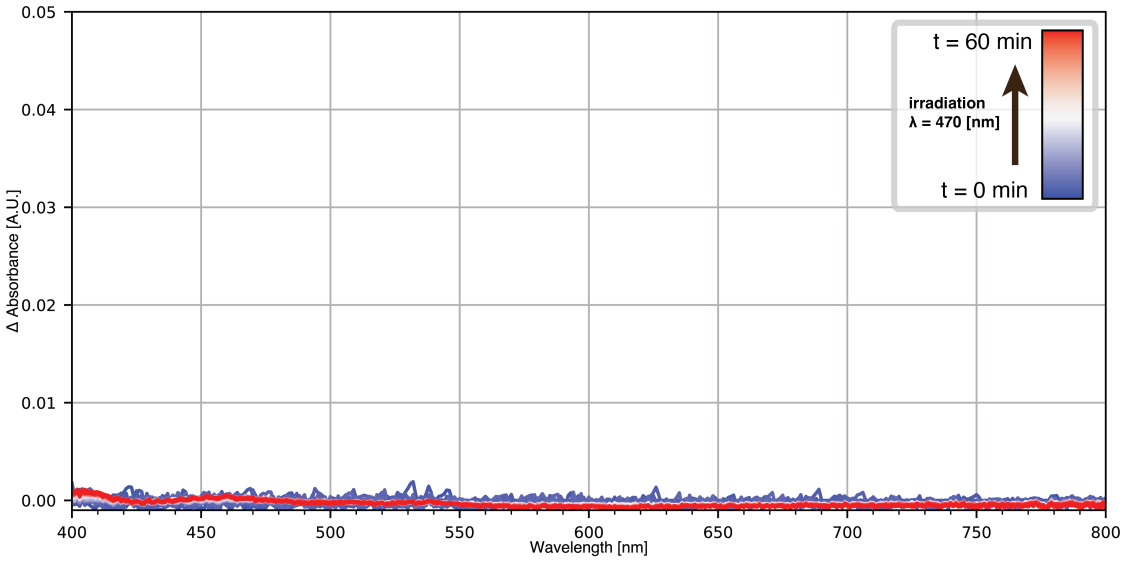

Figure S28: $[Ir(dCF_3) - PF_6]$ dissolved in fluorobenzene (fbz) with a concentration of 0.12 mM and irradiated with 0.655 mW of 470 nm light in the photoreactor setup described above. A total of 120 spectra were collected every 30 seconds. The first absorption spectrum is subtracted from each spectrum resulting in the resulting difference spectra.

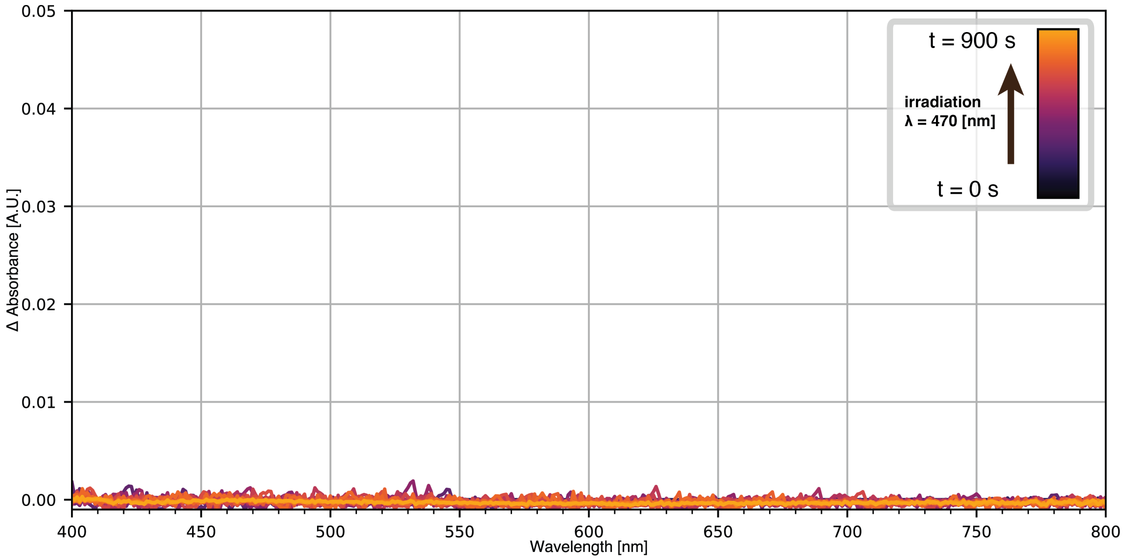

Figure S29: $[Ir(dCF_3) - PF_6]$ dissolved in fluorobenzene (fbz) with a concentration of 0.12 mM and irradiated with 0.655 mW of 470 nm light in the photoreactor setup described above. A total of 10 spectra were collected every 30 seconds. The first absorption spectrum is subtracted from each spectrum resulting in the resulting difference spectra.

Tetrahydrofuran:

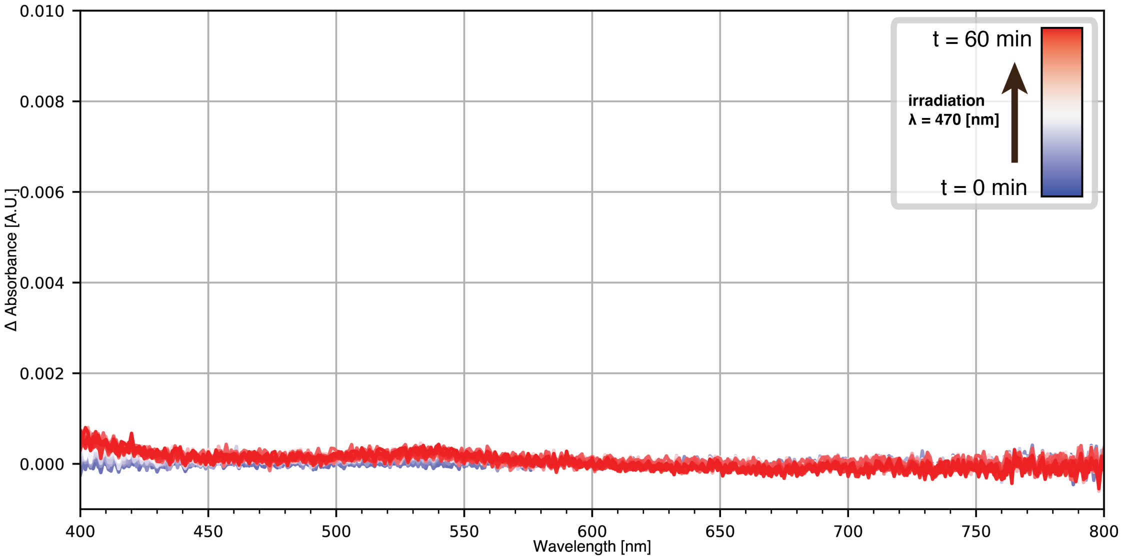

Figure S30: $[Ir(dCF_3) - BAr_4^F]$ dissolved in tetrahydrofuran (thf) with a concentration of 0.069 mM and irradiated with 0.655 mW of 470 nm light in the photoreactor setup described above. A total of 120 spectra were collected every 30 seconds. The first absorption spectrum is subtracted from each spectrum resulting in the resulting difference spectra.

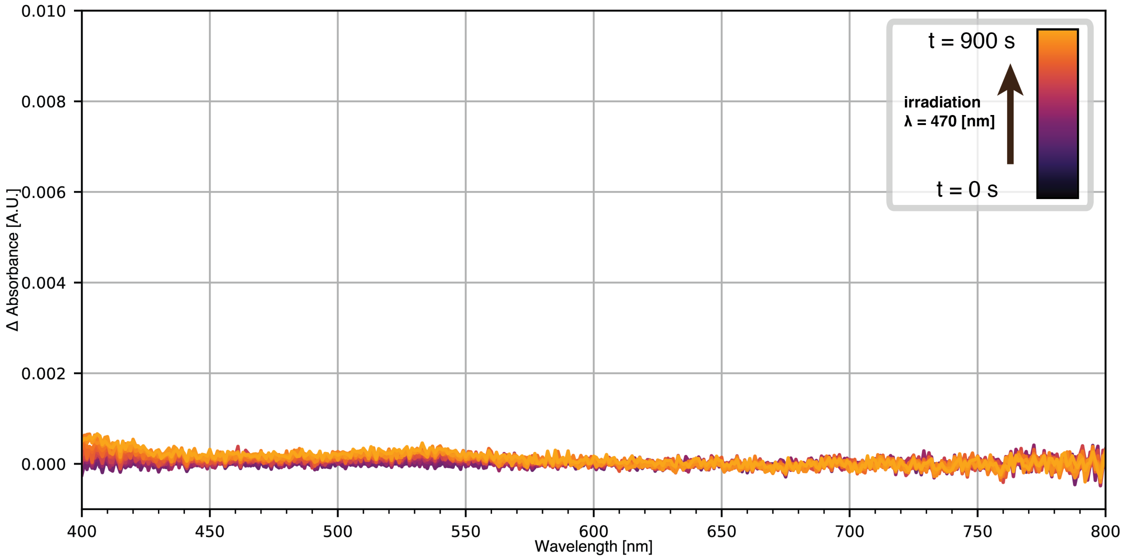

Figure S31: $[Ir(dCF_3) - BAr_4^F]$ dissolved in tetrahydrofuran (thf) with a concentration of 0.069 mM and irradiated with 0.655 mW of 470 nm light in the photoreactor setup described above. A total of 10 spectra were collected every 30 seconds. The first absorption spectrum is subtracted from each spectrum resulting in the resulting difference spectra.

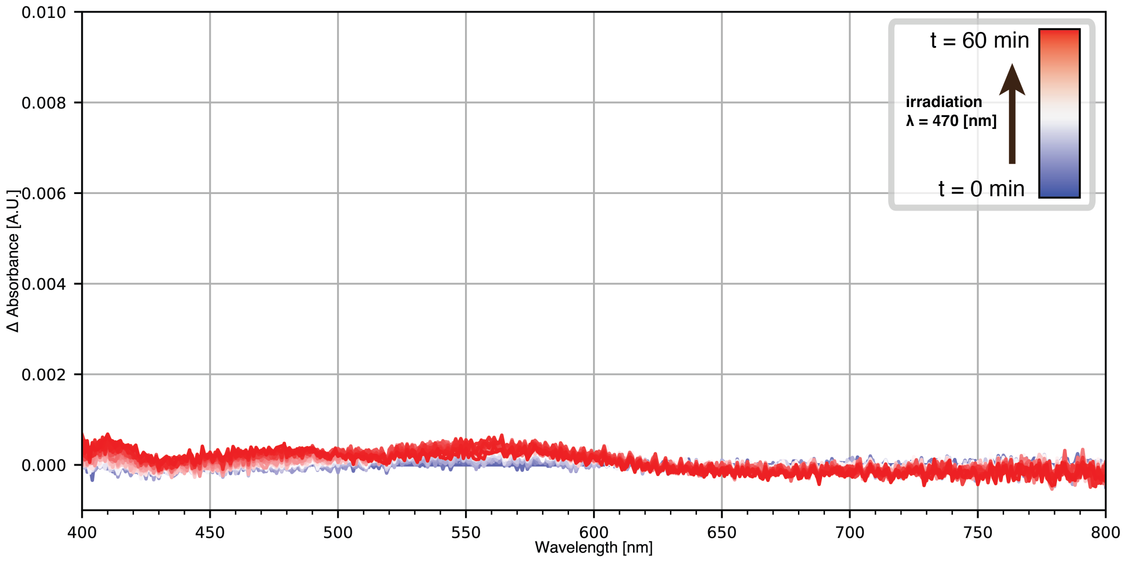

Figure S32: $[Ir(dCF_3)-PF_6]$ dissolved in tetrahydrofuran (thf) with a concentration of 0.12 mM and irradiated with 0.655 mW of 470 nm light in the photoreactor setup described above. A total of 120 spectra were collected every 30 seconds. The first absorption spectrum is subtracted from each spectrum resulting in the resulting difference spectra.

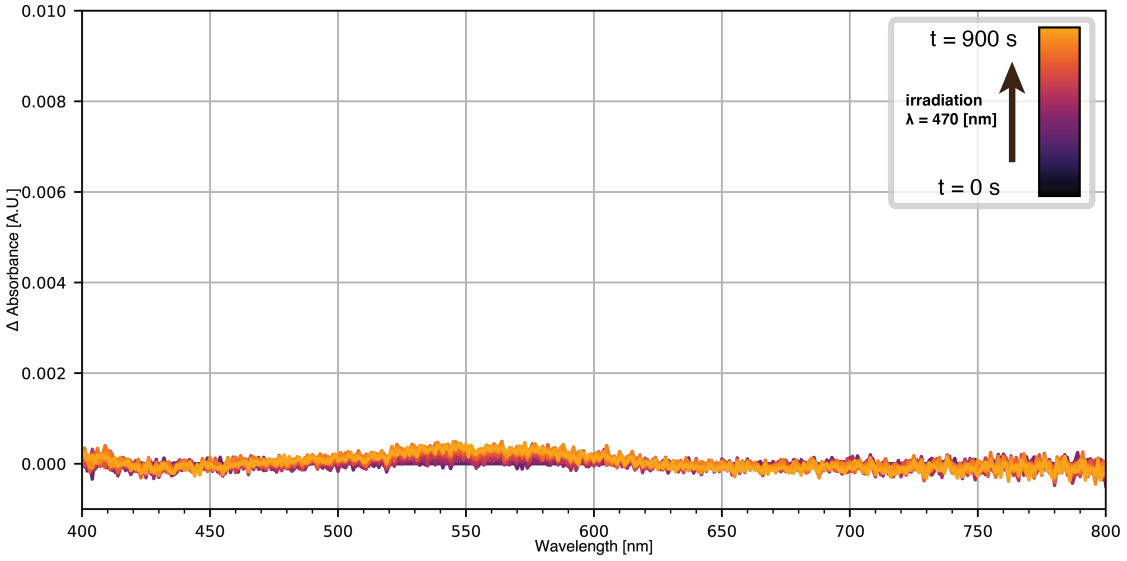

Figure S33: $[Ir(dCF_3)-PF_6]$ dissolved in tetrahydrofuran (thf) with a concentration of 0.12 mM and irradiated with 0.655 mW of 470 nm light in the photoreactor setup described above. A total of 10 spectra were collected every 30 seconds. The first absorption spectrum is subtracted from each spectrum resulting in the resulting difference spectra.

α, α, α- trifluorotoluene:

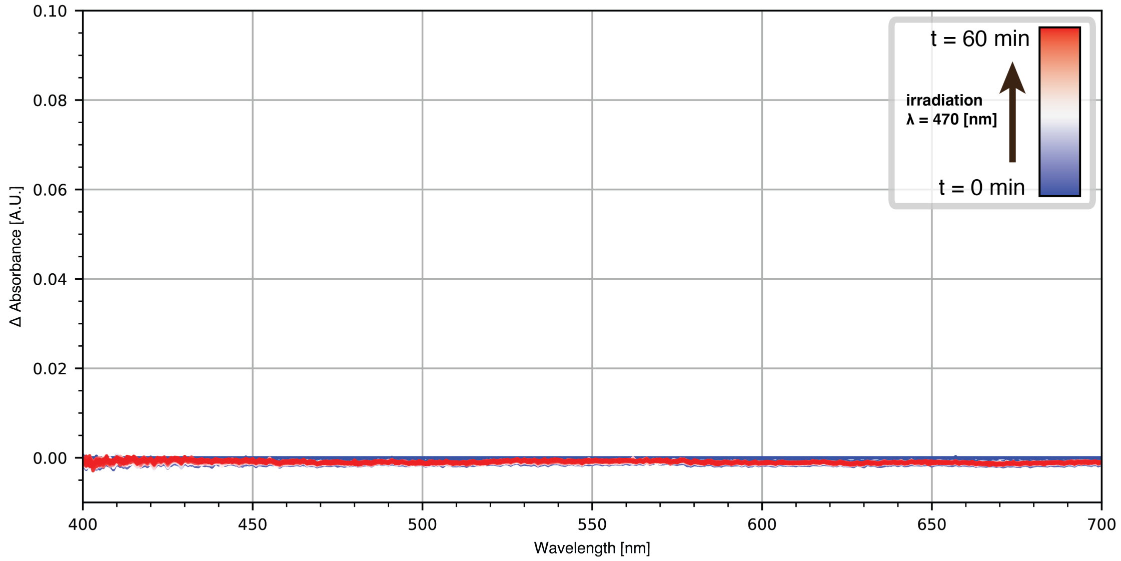

Figure S34: $[Ir(dCF_3) - BAr_4^F]$ dissolved in α, α, α- trifluorotoluene (tft) with a concentration of 0.078 mM and irradiated with 0.655 mW of 470 nm light in the photoreactor setup described above. A total 120 spectra were collected every 30 seconds. The first absorption spectrum is subtracted from each spectrum resulting in the resulting difference spectra.

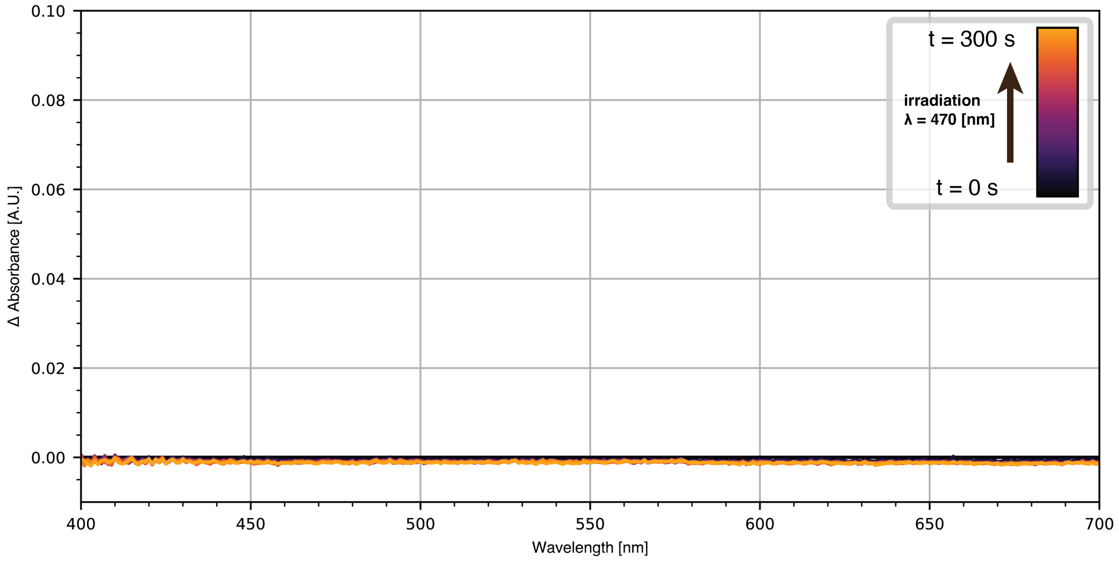

Figure S35: $[Ir(dCF_3) - BAr_4^F]$ dissolved in α, α, α- trifluorotoluene (tft) with a concentration of 0.078 mM and irradiated with 0.655 mW of 470 nm light in the photoreactor setup described above. A total 10 spectra were collected every 30 seconds. The first absorption spectrum is subtracted from each spectrum resulting in the resulting difference spectra.

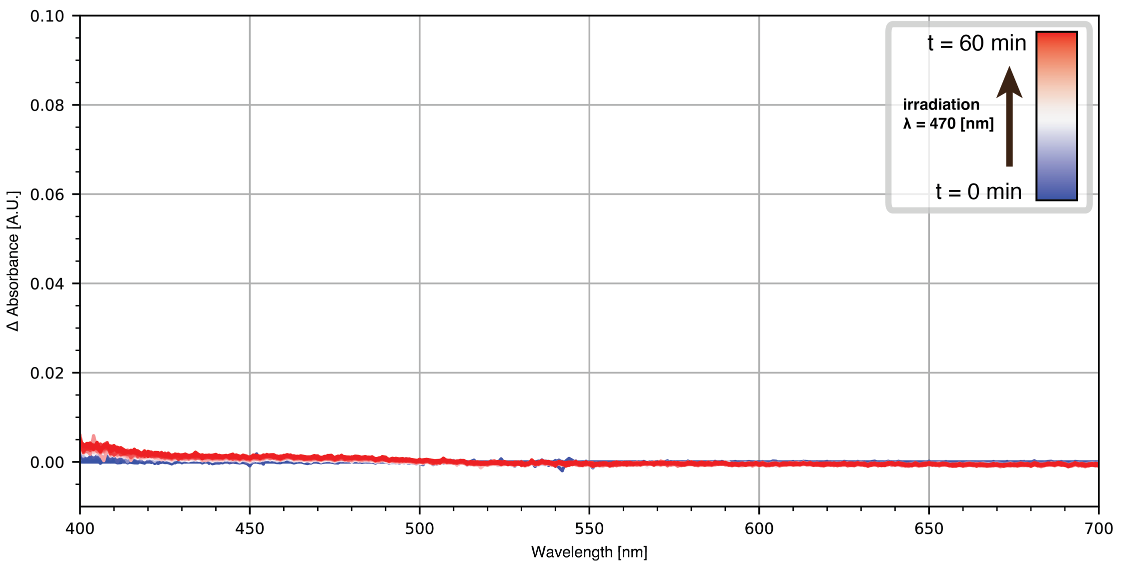

Figure S36: $[Ir(dCF_3) - PF_6]$ dissolved in α, α, α- trifluorotoluene (tft) with a concentration of 0.13 mM and irradiated with 0.655 mW of 470 nm light in the photoreactor setup described above. A total of 120 spectra were collected every 30 seconds. The first absorption spectrum is subtracted from each spectrum resulting in the resulting difference spectra.

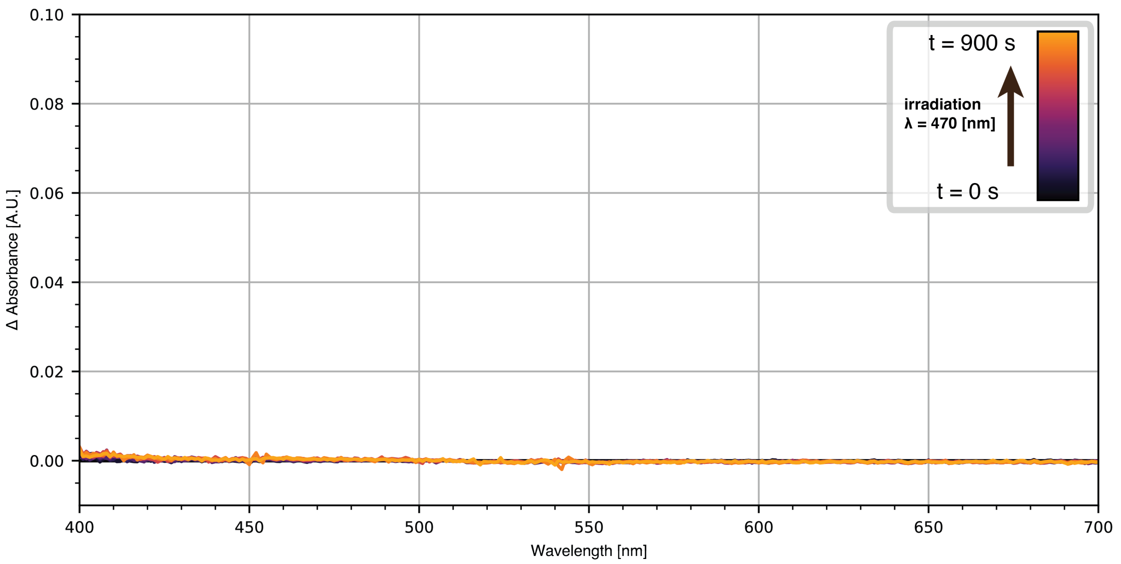

Figure S37: $[Ir(dCF_3) - PF_6]$ dissolved in α, α, α- trifluorotoluene (tft) with a concentration of 0.13 mM and irradiated with 0.655 mW of 470 nm light in the photoreactor setup described above. A total of 10 spectra were collected every 30 seconds. The first absorption spectrum is subtracted from each spectrum resulting in the resulting difference spectra

Acetonitrile:

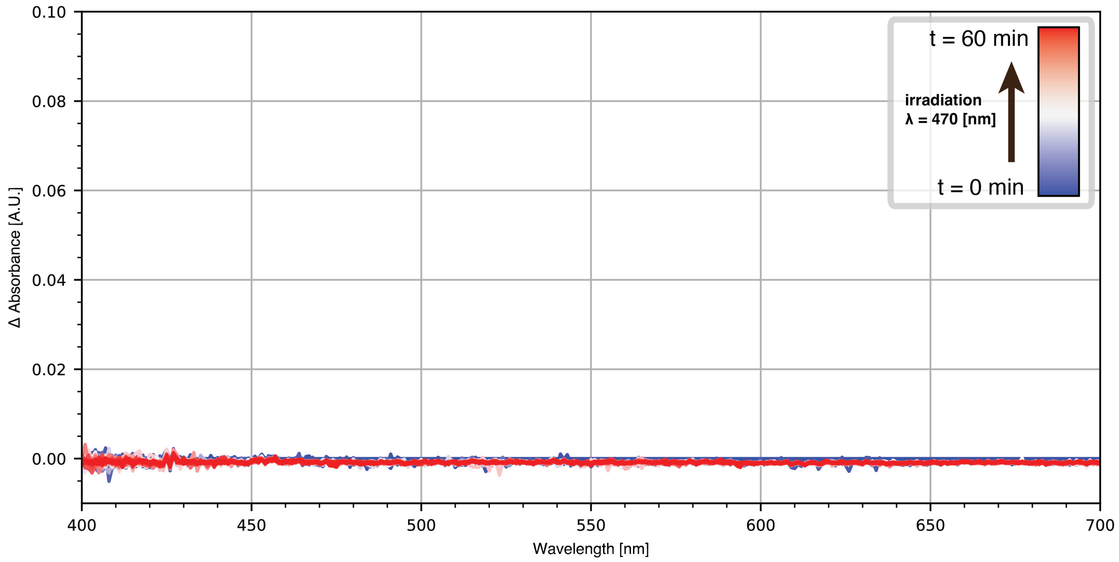

Figure S38: $[Ir(dCF_3) - BAr_4^F]$ dissolved in acetonitrile (acn) with a concentration of 0.094 mM and irradiated with 0.655 mW of 470 nm light in the photoreactor setup described above. A total 120 spectra were collected every 30 seconds. The first absorption spectrum is subtracted from each spectrum resulting in the resulting difference spectra.

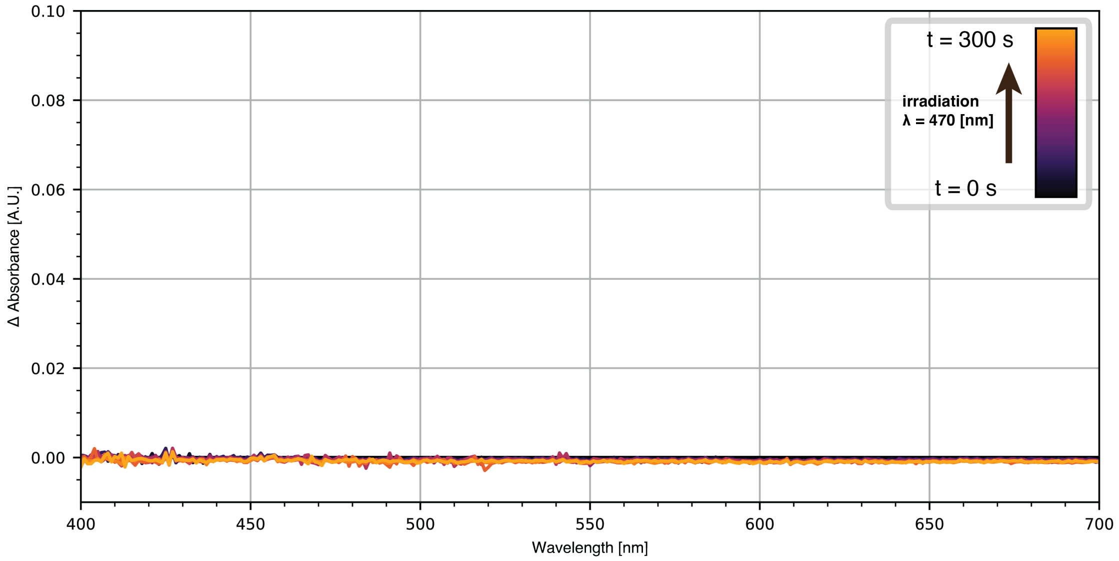

Figure S39: $[Ir(dCF_3) - BAr_4^F]$ dissolved in acetonitrile (acn) with a concentration of 0.094 mM and irradiated with 0.655 mW of 470 nm light in the photoreactor setup described above. A total 10 spectra were collected every 30 seconds. The first absorption spectrum is subtracted from each spectrum resulting in the resulting difference spectra.

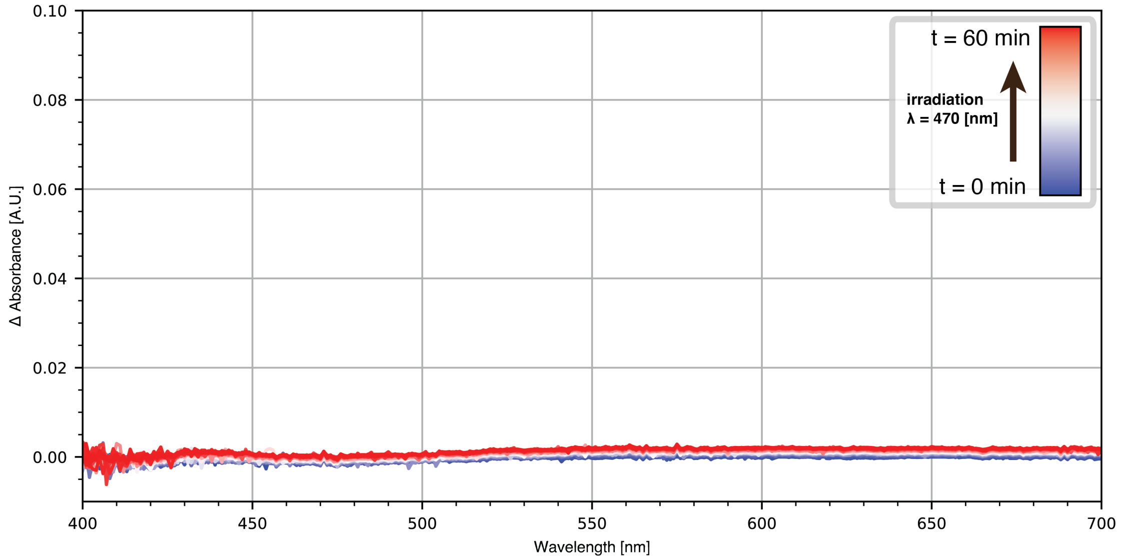

Figure S40: $[Ir(dCF_3) - PF_6]$ dissolved in acetonitrile (acn) with a concentration of 0.13 mM and irradiated with 0.655 mW of 470 nm light in the photoreactor setup described above. A total of 120 spectra were collected every 30 seconds. The first absorption spectrum is subtracted from each spectrum resulting in the resulting difference spectra

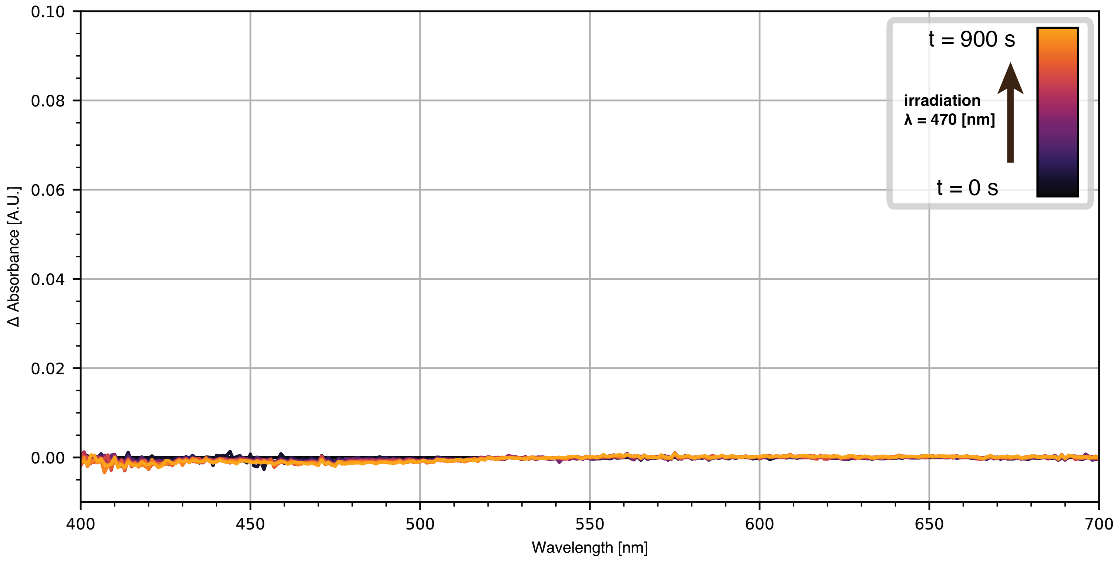

Figure S41: $[Ir(dCF_3) - PF_6]$ dissolved in acetonitrile (acn) with a concentration of 0.13 mM and irradiated with 0.655 mW of 470 nm light in the photoreactor setup described above. A total of 10 spectra were collected every 30 seconds. The first absorption spectrum is subtracted from each spectrum resulting in the resulting difference spectra

1.1.1 Integrated absorption as a function of irradiation time

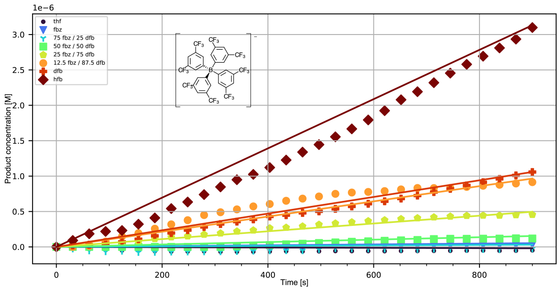

Figure S42: The change in the concentration of $[Ir(dCF_3^{\cdot-})]^0$ as a function of time in the range of solvents for $[Ir(dCF_3) - BAr_4^F]$. The lines are linear fits to the data points used to calculated the composite rate constant k_{rxn} as described below.

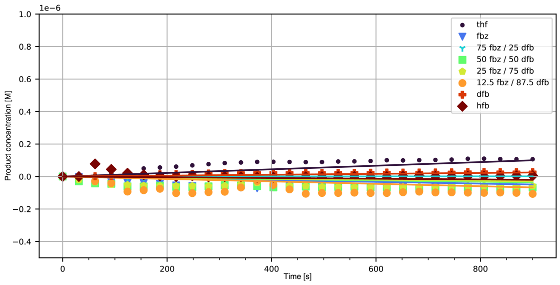

Figure S43: The change in the concentration of $[Ir(dCF_3^{\cdot-})]^0$ as a function of time in the range of solvents for $[Ir(dCF_3) - PF_6]$. The lines are linear fits to the data points used to calculated the composite rate constant k_{rxn} as described below.

To calculate the rate constants of the PET as seen in Equation 1, we need to know the rate of production of the product molecule.

$$Rate = \frac{\partial[P]}{\partial t} = k_{rxn}[R] \tag{1}$$

By rearranging Equation 1 we can isolate k_{rxn} on the left-hand side of the equation and its form is shown in Equation 2.

$$k_{rxn} = \frac{\partial[P]}{\partial t[R]} \tag{2}$$

Due to instrumentation constraints, a continuous change is not attainable for the product concentration nor the time, therefore, the change can be made discrete and it assumed that the rate in between points remains constant as shown in Equation 3

$$k_{rxn} = \frac{\Delta[P]}{\Delta t[R]} \tag{3}$$

The value of $\frac{\Delta[P]}{\Delta t}$ can be thought of as a slope for a linear curve and can be obtained through the linear regression of the plot of [P] vs time as in Figure S42 and S43. The concentration of product molecule for each time point is determined by taking

the integral of the absorption spectra at a certain time duration of irradiation from 525 - 600 nm to account for any broadening or shifts in oscillator strength in the spectra in the various solvents for the product molecule. With the known molar extinction coefficient over the same wavelength region, Beer-Lambert law can be used to calculate the concentration of product molecule, where l is the pathlength and ϵ_M is the molar exctinction coefficient. This is shown in mathematical form in Equation 4

$$[P] = \frac{\int_{525}^{600} A(\lambda) d\lambda}{l \cdot \int_{525}^{600} \epsilon_M d\lambda} \tag{4}$$

Finally, the reactants are not just the concentration of iridium complexes in the solution, but their excited-state population. This can be calculated for the steady-state equilibrium concentration of excited states with Equation 5, where F is the flux of the continuous wave light source in $n_{ph}/cm^2/s$, Φ_A is the optical density of the solution at the excitation wavelength, τ_r is the excited-state lifetime of the chromophore, and l is the pathlength of the cuvette.

$$[R] = \frac{F \Phi_A \tau_r}{l} \tag{5}$$

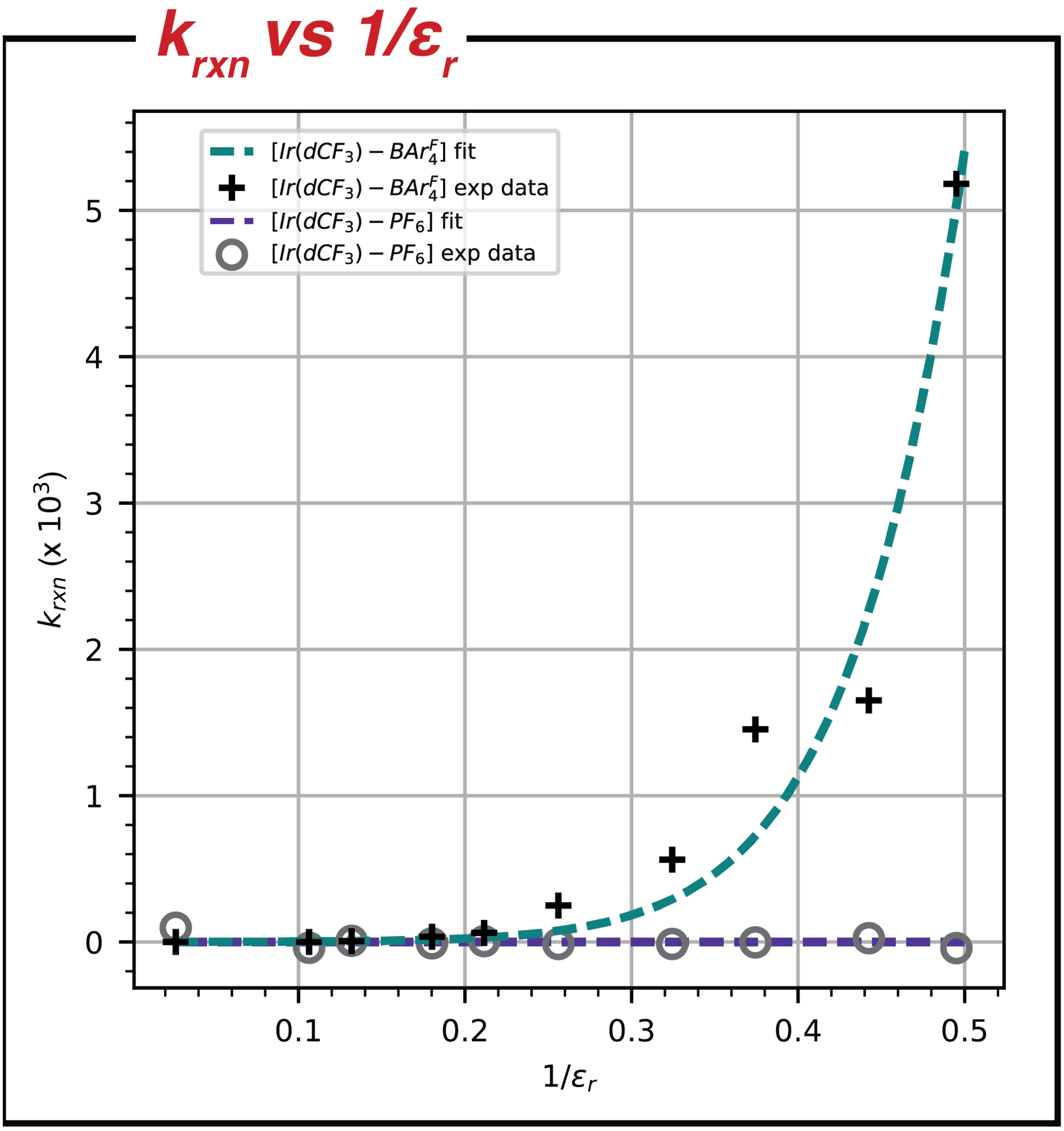

Figure S44: Experimental spectrum and fit for $[Ir(dCF_3)-BAr_4^F]$ and $[Ir(dCF_3)-PF_6]$, irradiated for 60 min time (470 nm LED, 0.6 mW power). Fit parameters to the Marcus Theory equation, electronic coupling (V) = 0.086 cm^{-1}, reorganization energy (λ) = 4.10 eV for only $[Ir(dCF_3)-BAr_4^F]$ data set. The large value for the reorganization energy is most likely due to the degradation of $[BAr_4^F]^-$ upon oxidation.

1.2 Electron paramagnetic resonance (EPR)

Instrumentation

For continuous wave electron paramagnetic resonance experiments, X-band (9.37 GHz) data were collected on an Elexsys E500 spectrometer equipped with a super high-Q resonator (Bruker, USA), cryogen-free helium system (ColdEdge Technologies, USA), and MercuryiTC temperature controller (Oxford Instruments, UK).

Sample preparation and collection parameters

$[Ir(dCF_3) - BAr_4^F]$ was prepared by dissolving 0.48 mg of solid in 5 mL of hexafluorobenzene to form a 0.0515 mM solution inside a N2-filled glovebox. 3 mL of the solution was transferred to a 1 cm quartz cuvette and irradiated in the custom photoreactor setup (470 nm, 29.0 mW power) for 10 minutes. The cuvette was brought back into the glovebox and 100 μL of the irradiated solution was transferred to an EPR tube fitted with a Teflon screw cap closure (Wilmad 727-LPV-250M) and the tube was removed from the glovebox. The sample was frozen in liquid nitrogen and inserted into the instrument where it was further cooled to the measurement temperature of 4.0 K or 10.0 K. Data was acquired with the following instrument parameters unless otherwise indicated.

Table S1: Summary of EPR parameter values

**Parameter**	**Value**
MW frequency	9.3720 GHz
MW power	0.1002 mW
Signal Averages	3
Gain	60 dB
Field resolution	512 pts
Conversion Time	80.0 ms
Modulation Amplitude	10.0 G
Modulation Frequency	100 kHz
Temperature	10.0 K

Simulation of EPR Spectra

The EasySpin toolbox[1] and the function "pepper" within Matlab version R2023A (MathWorks) was utilized for simulations. The Nelder-Mead optimization method for least squares fitting was chosen along with a target of the "data as is".

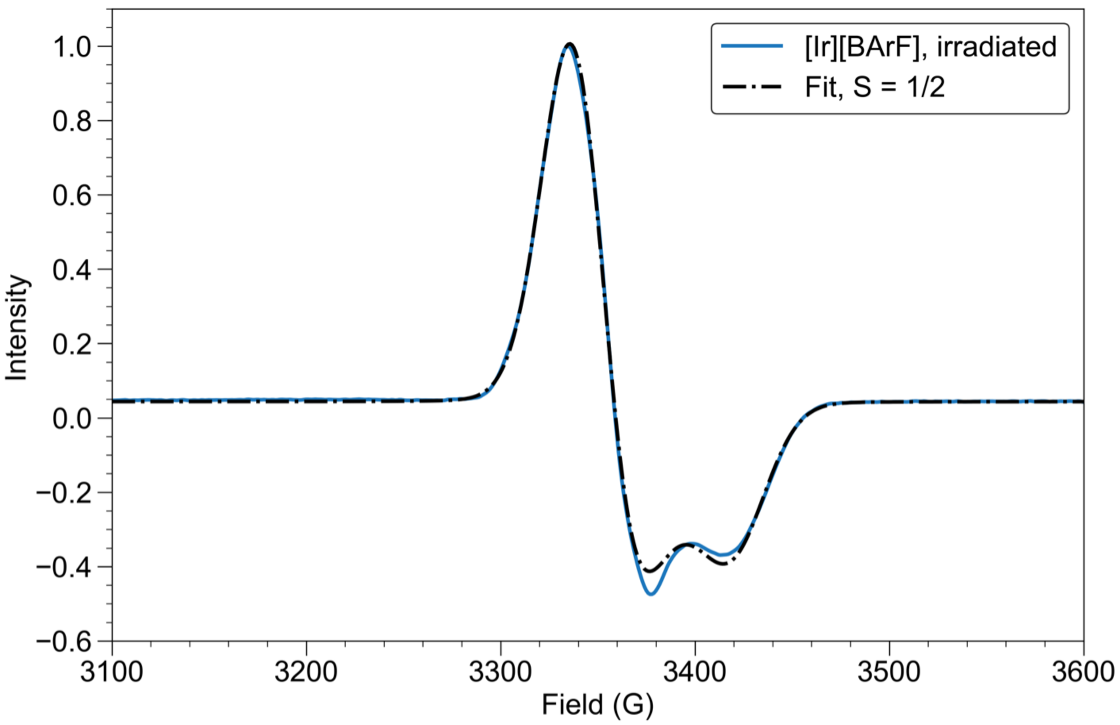

Figure S45: Experimental spectrum and fit for [Ir][BArF], irradiated for X time (470 nm LED, 29.0 mW power). Simulation parameters given below.

Table S2: Summary of EPR parameter values

**Parameter**	**Value**
g_x	2.0015
g_y	2.0015
g_z	1.9580
S	1/2
lw (gaussian)	4.016
lw (lorrenzian)	0.196

These output parameters agree well with reported EPR data for singly reduced cationic Ir(III) complexes where axial symmetry is typically observed with gx = gy ¡ gz. As such, this EPR data constitutes strong evidence for the formation of a reduced Ir complex via irradiation of the [Ir][BArF] solution.

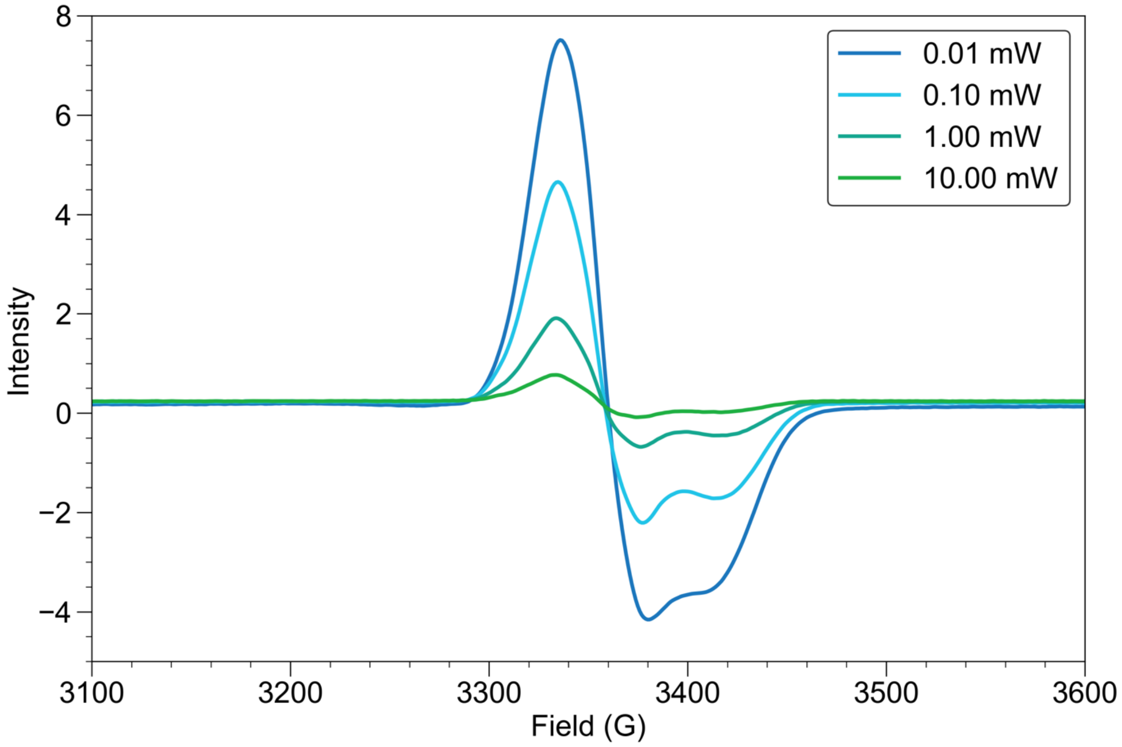

Figure S46: EPR spectra for [Ir][BArF], irradiated for X time (365 nm LED, X power) with varying MW power from 0.01-10.00 mW.

No significant changes in spectral shape are observed as a function of power. 33 dB attenuation (0.1 mW power) was chosen for fitting as it was determined qualitatively to have the best signal to noise.

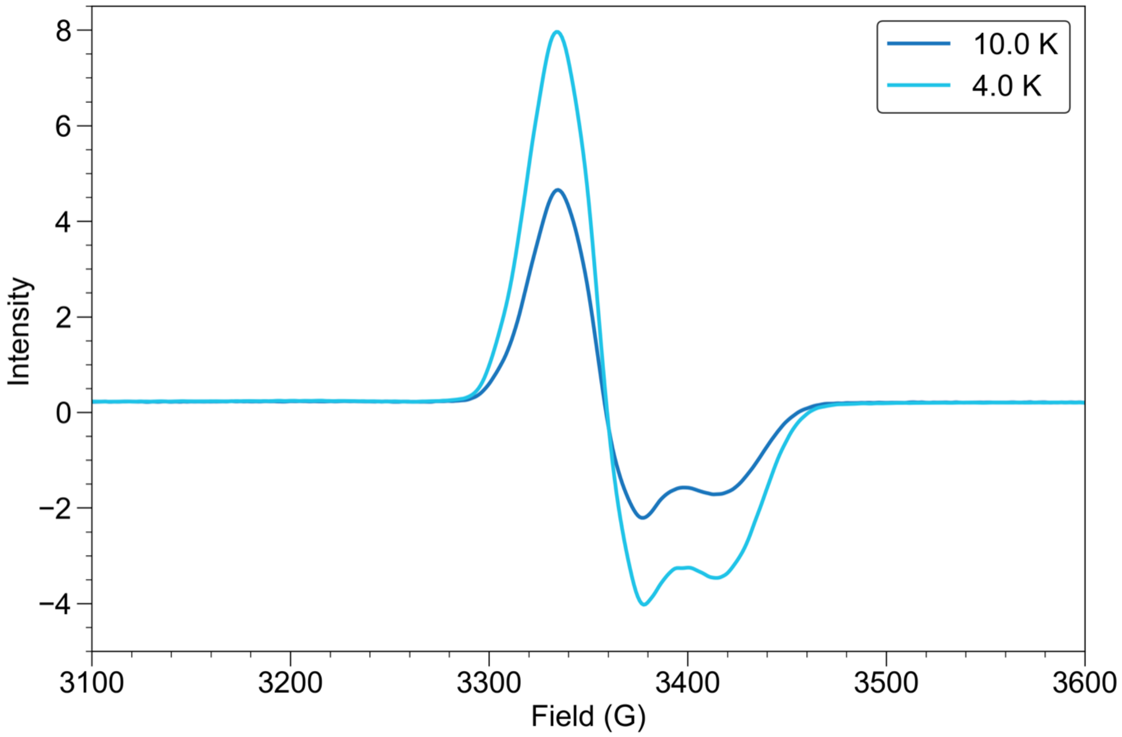

Figure S47: EPR spectra for [Ir][BArF], irradiated for X time (365 nm LED, X power) acquired at temperatures T = 10.0 K or T = 4.0 K.

No significant changes in spectral shape are observed as a function of temperature. 10.0 K was chosen for fitting for the most straightforward comparison with literature data.[2]

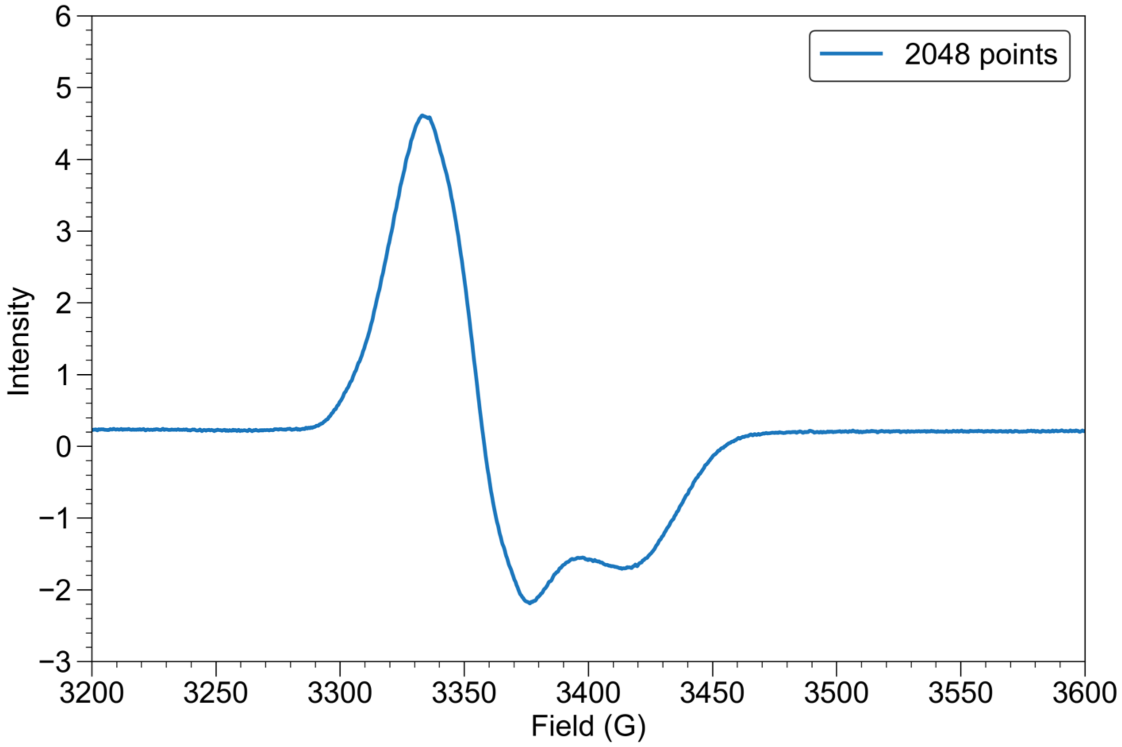

Figure S48: EPR spectrum for [Ir][BArF], irradiated for X time (470 nm LED, 29.0 mW) acquired with 2048 x-axis points as opposed to 512 points as used for all other spectra.

Increasing the field resolution does not reveal any new features, suggesting 512 points is sufficient.

1.3 Nuclear Magnetic Resonance (NMR)

Solutions of $[Ir(dCF_3) - BAr_4^F]$ and $[Ir(dCF_3) - PF_6]$ at 5 mM were prepared in hexafluorobenzne (Sigma 326720-5G). NMR spectra were taken of each solution using a Bruker 400 MHz NMR spectrometer. Each solution was monitored before and after irradiation with a 456 nm Kessel lamp with a duration of 10 minutes. Figure S49 shows no change in the NMR spectra before and after irradiation indicating no reactions have occurred in the solution containing $[Ir(dCF_3) - PF_6]$. Figure S50 shows the degradation of the $[BAr_4^F]^-$ anion after irradiation. The peaks located at δ 7.65 and 7.54 ppm, also shaded in a red box in the figure are assigned to the hydrogens located on $[BAr_4^F]^-$. The intensity of these two peaks decrease after irradiation indicating a decrease in the concentration of $[BAr_4^F]^-$ in the solution. This reduction in concentration of $[BAr_4^F]^-$ is associated with the introduction of numerous peaks previously not present in the NMR spectra prior to irradiation demonstrating the degradation of $[BAr_4^F]^-$ upon irradation of the solution. All of these peaks associated with degradation products are designated with red arrows overlayed on the spectrum.

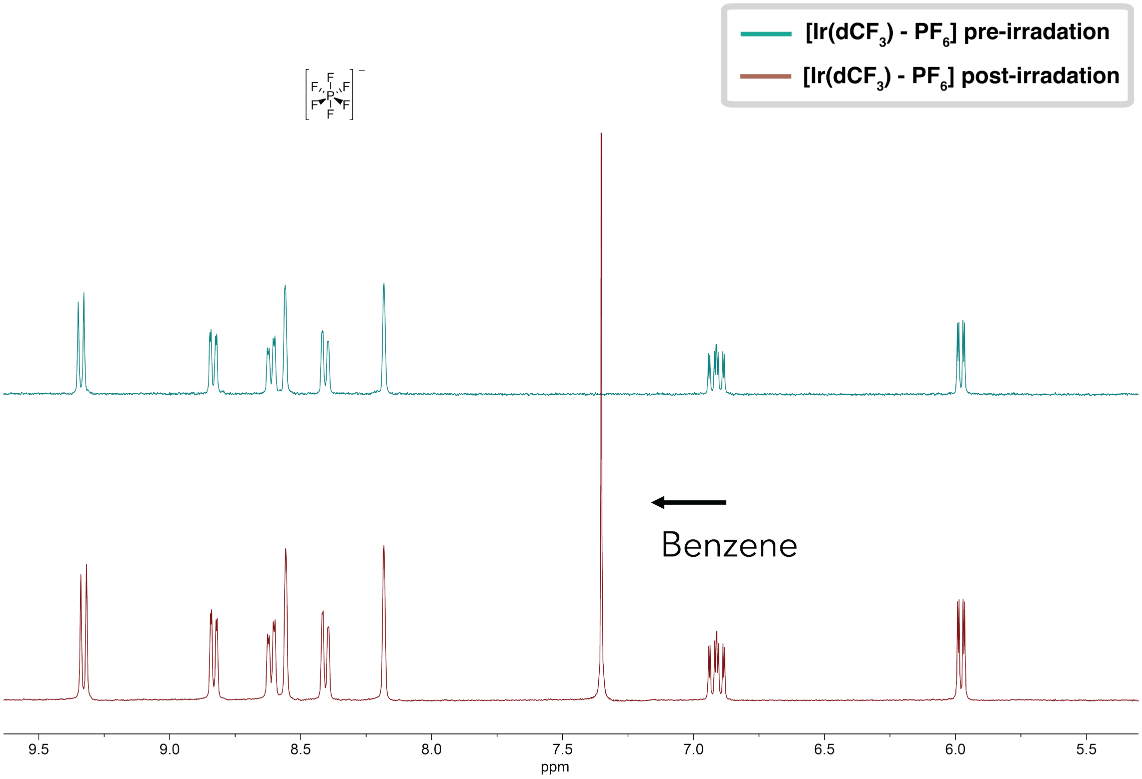

Figure S49: NMR spectra of $[Ir(dCF_3) - PF_6]$ in hfb before (blue) and after (red) irradiation

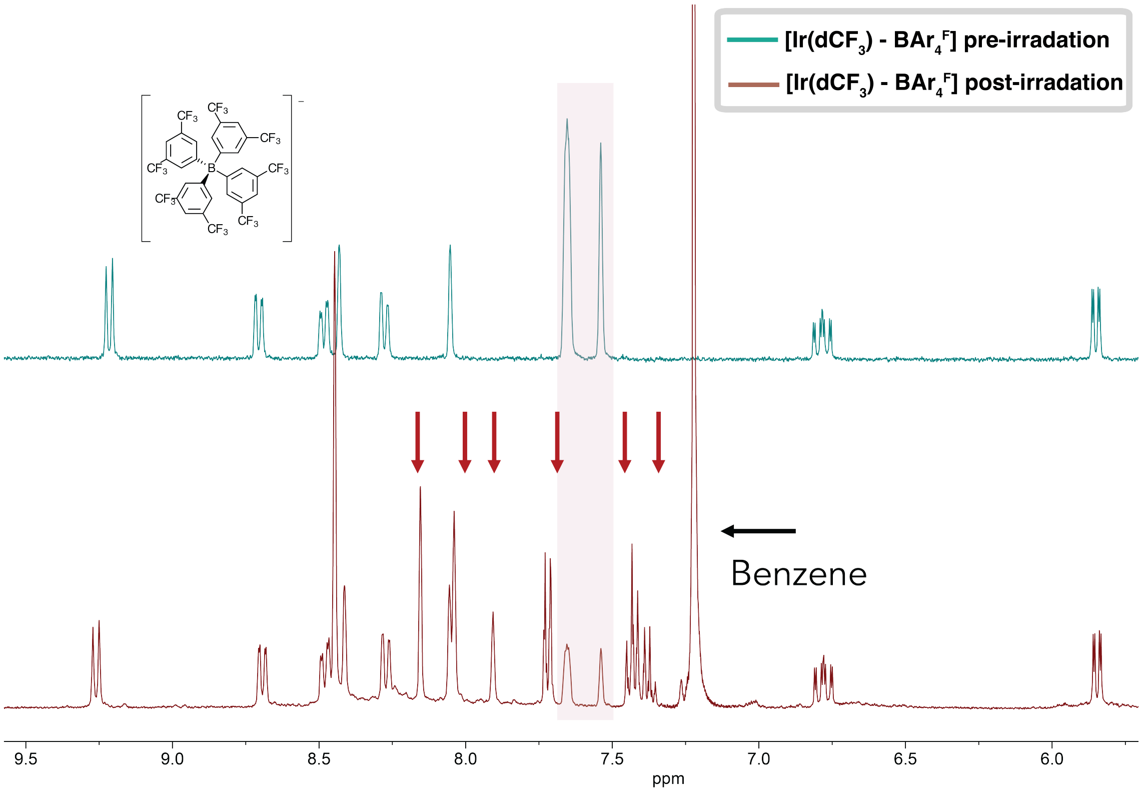

Figure S50: NMR spectra of $[Ir(dCF_3) - BAr_4^F]$ in hfb before (blue) and after (red) irradiation

1.4 Time-Resolved Photoluminescence (long time-scales)

The long-time photoluminescence is observed for $[Ir(dCF_3){-}BAr_4^F]$ and $[Ir(dCF_3){-}PF_6]$ in both hfb and acn using a Horiba Fluoromax-4. Each kinetic trace is excited at 365 nm and monitored at the photoluminescence maxiumum wavelength (625 nm) over 60 min. The normalized photon count for both ion-pairs in acn are do not deviate from their initial values indicating no loss in the lone fluorophore ($[Ir(dCF_3)]^+$) over the long exposure time. $[Ir(dCF_3)]^+$ is the lone fluorophore due to the inability for either counter-anion to absorb at the excitation wavelength and $[Ir(dCF_3^{\cdot-})]^0$ has been shown to be a doublet in the ground and excited-states, therefore, $[Ir(dCF_3^{\cdot-})]^0$ is presumed to not be luminescent in solution. The decay of both ion-pairs while in hfb indicate a decrease in concentration of $[Ir(dCF_3)]^+$ throughout the exposure.

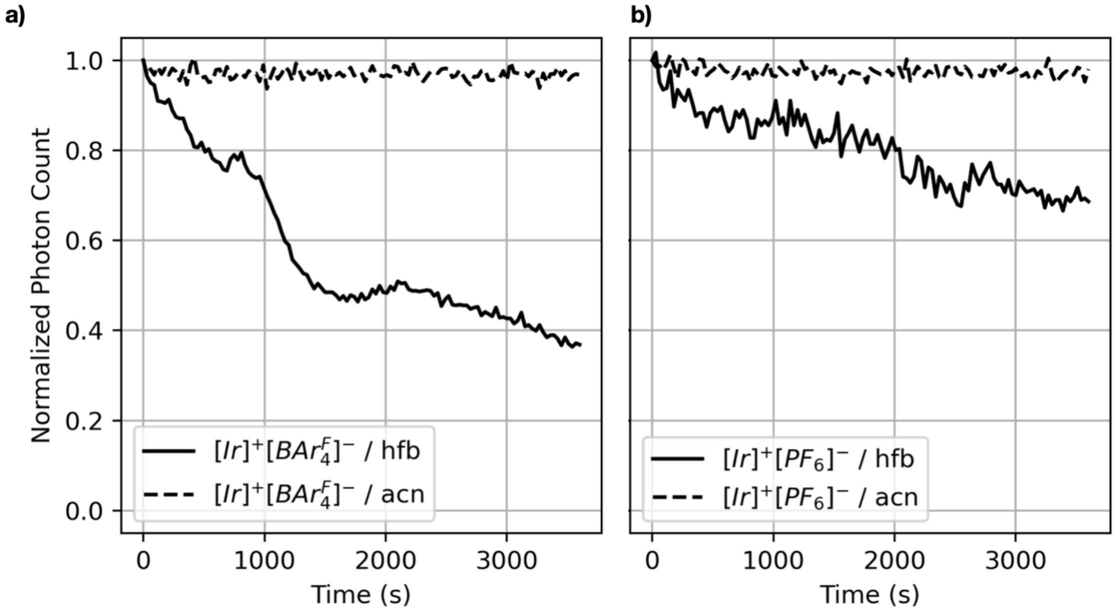

Figure S51: Long-time photoluminescence intensities of a) $[Ir(dCF_3) - BAr_4^F]$ and b) $[Ir(dCF_3) - PF_6]$ in hfb (solid) and acn (dashed).

1.5 Time-Resolved Photoluminescence (short time-scales)

The photoluminescence of both ion-pairs in hfb were measured to obtain the excited state lifetimes of $[Ir(dCF_3)]^+$. Optical excitation with ca. 20 ps pulses at 410 nm for either ion pair in solution was supplied by an NKT supercontinuum fiber laser (SuperK EXU-6-PP) with a 0.152 MHz repetition rate. A Hamamatsu 300–900 nm (C10910-04) streak camera was used to collect time-resolved PL spectra. Transients were analyzed at the wavelength of maximum PL intensity for each solution. The rate constant associated with the MLCT state of $[Ir(dCF_3)]^+$ when paired with $[BAr_4^F]^-$ is 993231 (s^{-1}), or $\tau = 1.01\ \mu s$, and with $[PF_6]^-$ is 1910270 (s^{-1}), or $\tau = 524$ ns.

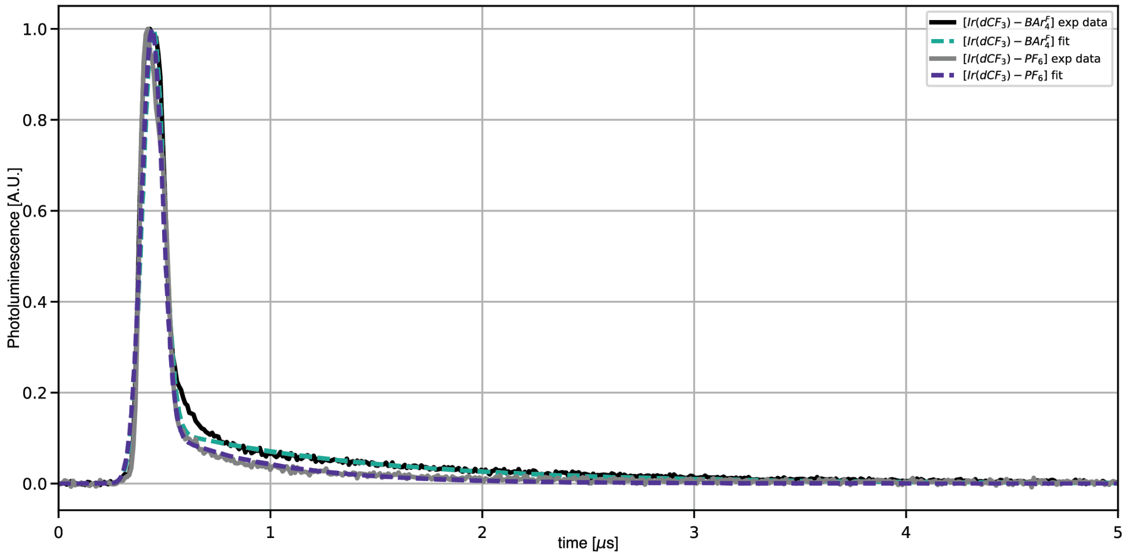

Figure S52: Time-Resolved Photoluminescence (TRPL) of $[Ir(dCF_3) - BAr_4^F]$ and $[Ir(dCF_3) - PF_6]$ in hfb. Both solutions were $\approx$ 0.05 mM.

1.6 Computational Methods

Geometry optimization and frequency calculations were carried out using Gaussian 16 software to obtain molecular radii and center-to-center distance between ions in solution.[3] Each optimized geomoetry was calculated with Density Functional Theory (DFT) with the B3LYP functional, lanl2dz basis set, gd3bj empirical dispersion, and a CPCM model of the perflorobenzene (i.e. hexafluorobenzene) solvent environment. Within each ion-pair system, the Gaussian Fragments option was utilized to maintain charge separation and spin of the ions during the computation. The motivation for using this set of computational parameters is that the B3LYP functional paired with the lanl2dz basis set has been shown to perform the best when heavy atoms, such as iridium, are present in the system.[4] The gd3bj has also been shown to account for intermolecular interactions such as those present in our ion-pair systems. Finally, the dielectric environment has been shown to significantly impact the energetics of the chemical system, therefore, a CPCM model of the hexafluorobenzene solvent environment was used. The xyz coordinates of the optimized structures are included with each ion-pair system.

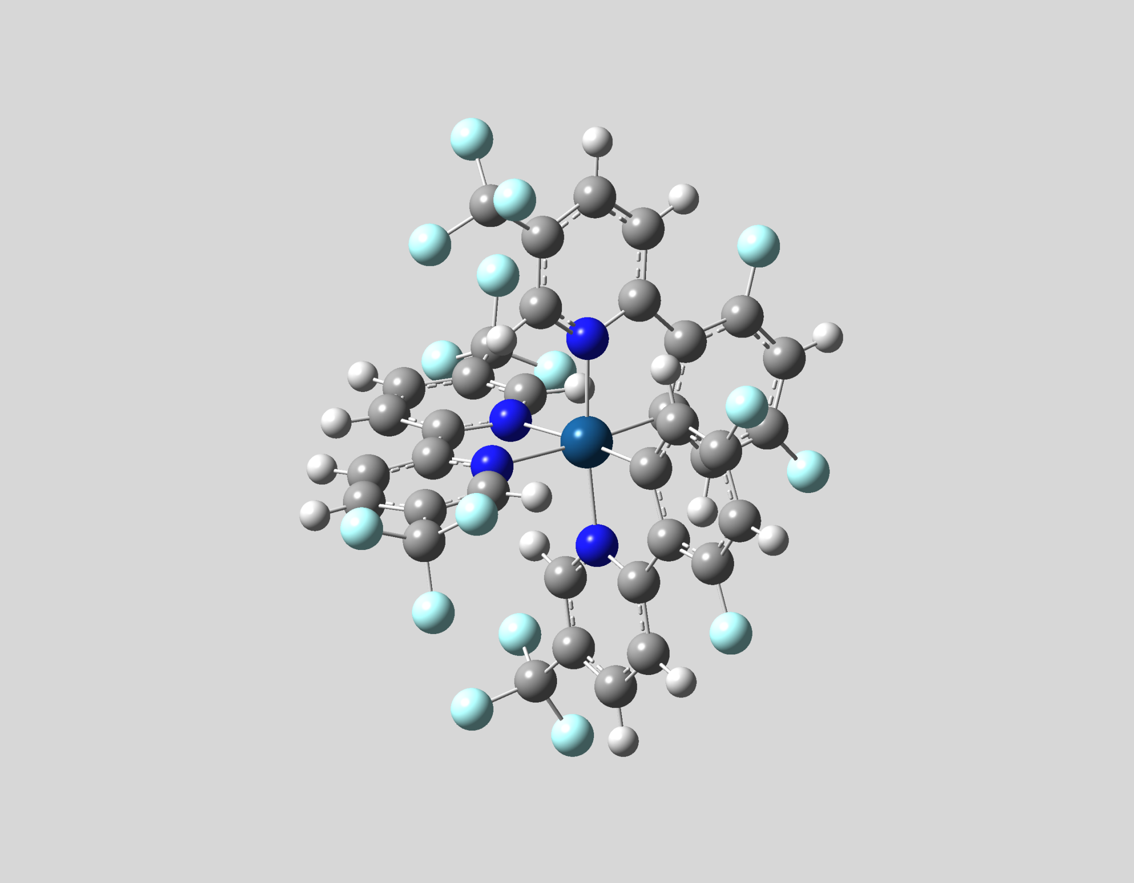

Figure S53: $[Ir(III)(dFCF_3ppy)_2\text{-}(5,5'\text{-}dCF_3bpy)]^+$ (abbv. $[Ir(dCF_3)]^+$) optimized geometry

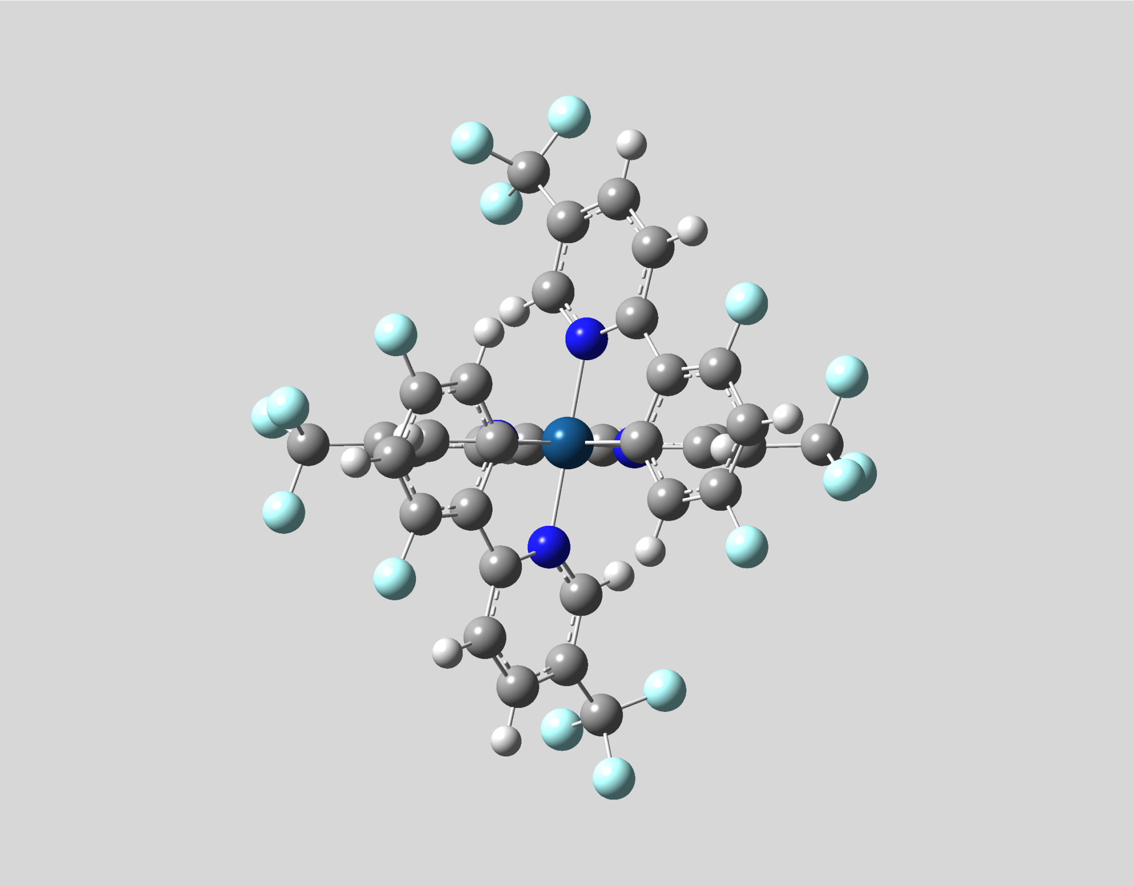

Figure S54: [Ir(III)(dFCF$_3$ppy)$_2$-(5,5'-dCF$_3$bpy)]$^+$ (abbv. [Ir(dCF$_3$)]$^+$) optimized geometry

XYZ Coordinates of optimized structure of [Ir(III)(dFCF$_3$ppy)$_2$-(5,5'-dCF$_3$bpy)]$^+$:

Element	X	Y	Z
Ir	−0.00257	−0.00630	0.36066
C	−0.62586	−0.40389	−2.58646
C	−1.24799	−0.87072	−3.75883
C	−2.38372	−1.68835	−3.66698
C	−2.87402	−2.01874	−2.39311
C	−2.22238	−1.52525	−1.25350
N	−1.12420	−0.73571	−1.34675
H	−2.87748	−2.05225	−4.56215
H	−0.85869	−0.61042	−4.73519
H	−2.58011	−1.74605	−0.25504
C	2.19419	1.54936	−1.24942
C	2.83077	2.06773	−2.38670
C	2.32362	1.76519	−3.66067
C	1.18677	0.94927	−3.75514
C	0.58048	0.45668	−2.58519
H	2.80525	2.14906	−4.55406
H	2.56455	1.74898	−0.25103
H	0.78462	0.71023	−4.73173
C	4.02296	2.97440	−2.21554
C	−4.06289	−2.93088	−2.22829
F	4.81216	3.01806	−3.36221
F	4.83286	2.56588	−1.15131
F	3.62665	4.29277	−1.93610
F	−4.88661	−2.91897	−3.35154
F	−3.66118	−4.26188	−2.02572
F	−4.83885	−2.57376	−1.12177
N	1.09522	0.76153	−1.34519
C	−0.39632	2.71816	1.44410
C	−0.97854	3.99849	1.60759
C	−2.07290	4.38059	0.82720
C	−2.58916	3.47166	−0.11775
C	−1.99500	2.21482	−0.24556
N	−0.92939	1.84269	0.50668
H	−2.52366	5.35967	0.95593
H	−0.56864	4.67547	2.34328
H	−2.38456	1.48632	−0.94333
C	2.22815	0.24899	2.48060
C	2.90744	0.97855	3.46482
C	2.56219	2.28232	3.84146
C	1.47942	2.84527	3.17486
C	0.74708	2.17338	2.17093
H	3.10546	2.82267	4.60790
H	2.56376	−0.75942	2.26728
C	−3.72777	3.84527	−1.02100
F	−4.62470	4.71996	−0.41307
F	−3.27877	4.48084	−2.19697
F	−4.45118	2.71866	−1.44492
C	1.13205	0.83527	1.81890
F	3.98308	0.37760	4.10789
C	−0.72833	−2.22625	2.13129
C	−1.44706	−2.92030	3.12997

Element	X	Y	Z
C	−2.52026	−2.37219	3.82392
C	−2.86997	−1.06026	3.48138
C	−2.20372	−0.30889	2.50476
H	−3.05313	−2.92949	4.58552
H	−2.54157	0.70412	2.31854
C	1.98189	−2.21297	−0.32145
C	2.57388	−3.47412	−0.23389
C	2.06609	−4.40623	0.69322
C	0.98191	−4.04218	1.49595
C	0.40459	−2.75474	1.37649
H	2.50390	−5.39589	0.77878
H	2.35227	−1.47449	−1.01899
F	1.12151	4.14920	3.53667
N	0.92625	−1.85794	0.45307
C	−1.11705	−0.88031	1.81515
C	3.75608	−3.79987	−1.09958
F	3.76390	−3.02965	−2.27724
F	4.97881	−3.54571	−0.45783
F	3.77464	−5.14221	−1.48078
F	−1.08494	−4.23209	3.45754
F	−3.93663	−0.47356	4.15222
H	0.57465	−4.73949	2.21390

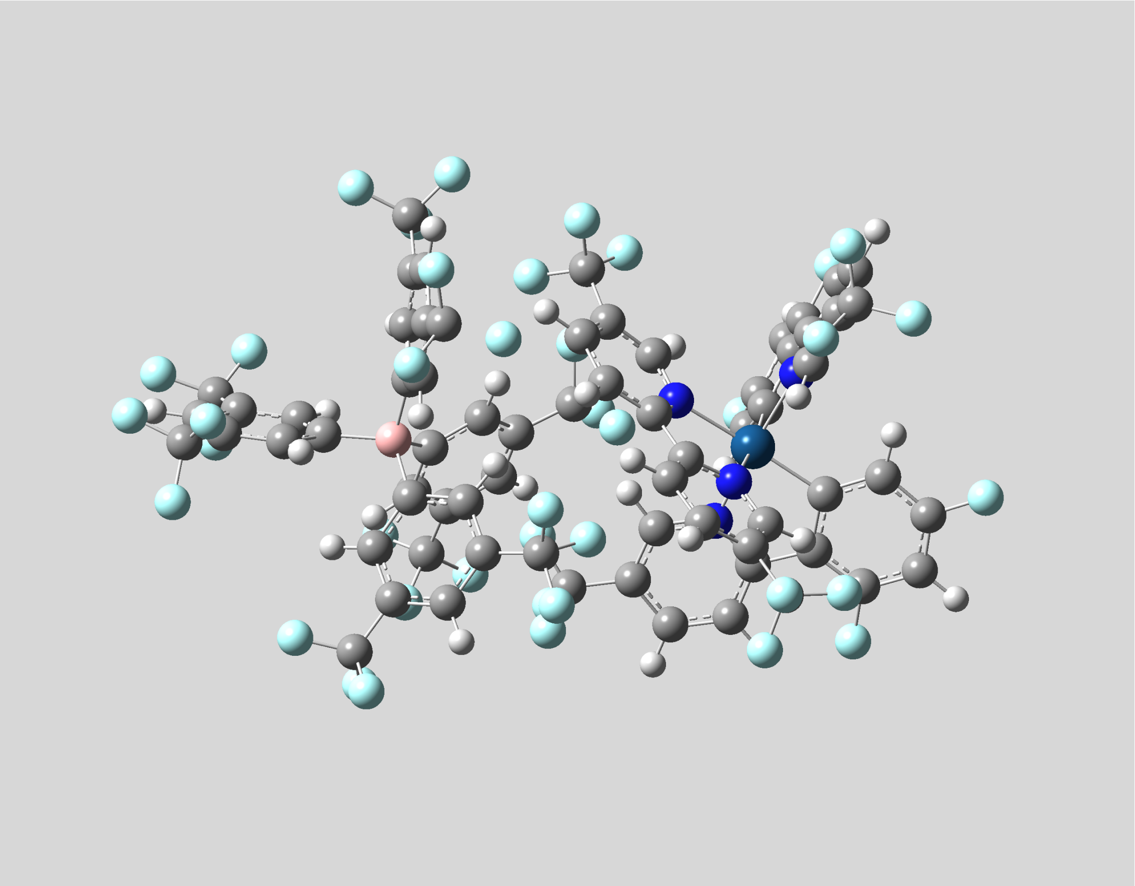

Figure S55: [Ir(III)(dFCF$_3$ppy)$_2$-(5,5'-dCF$_3$bpy)]$^+$ (abbv. [Ir(dCF$_3$)]$^+$) paired with tetrakis[3,5-bis(trifluoromethyl)phenyl]borate (abbv. $[BArF_4]^-$) optimized geometry

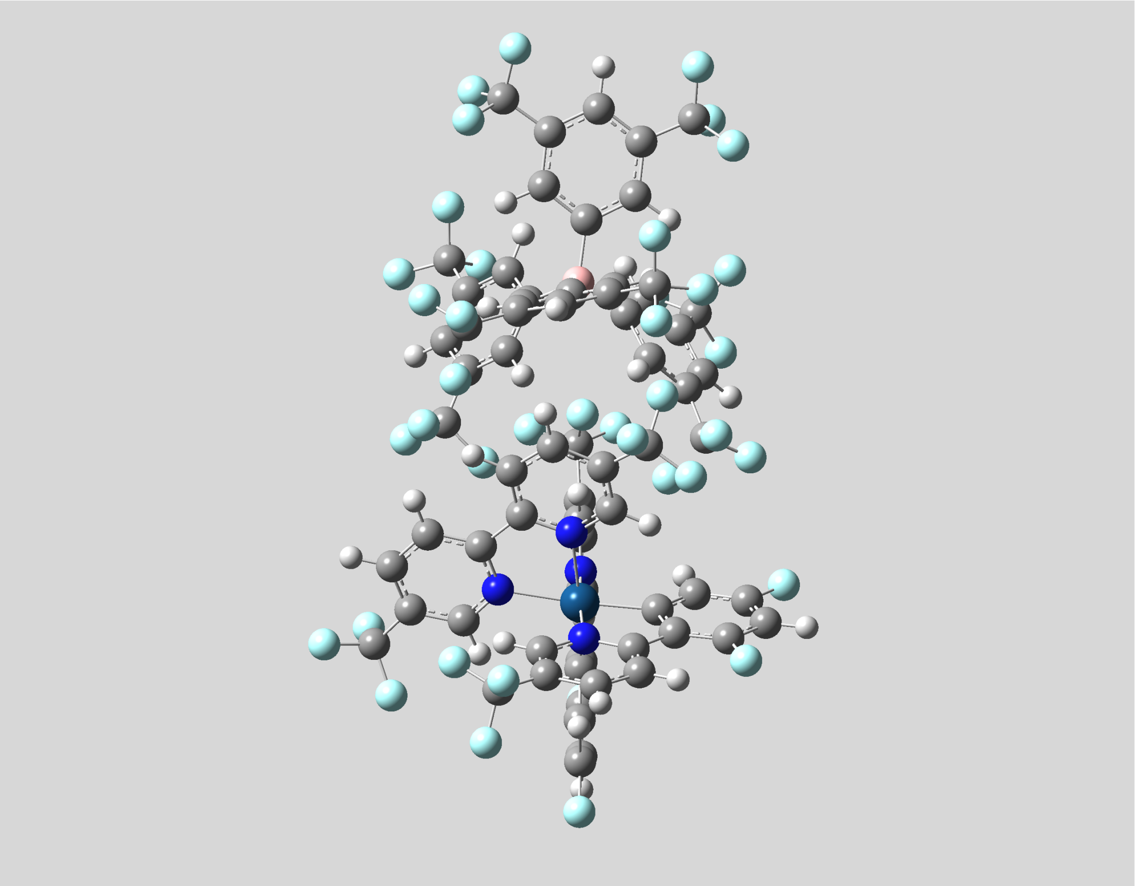

Figure S56: [Ir(III)(dFCF$_3$ppy)$_2$-(5,5'-dCF$_3$bpy)]$^+$ (abbv. [Ir(dCF$_3$)]$^+$) paired with tetrakis[3,5-bis(trifluoromethyl)phenyl]borate (abbv. $[BArF_4]^-$) optimized geometry

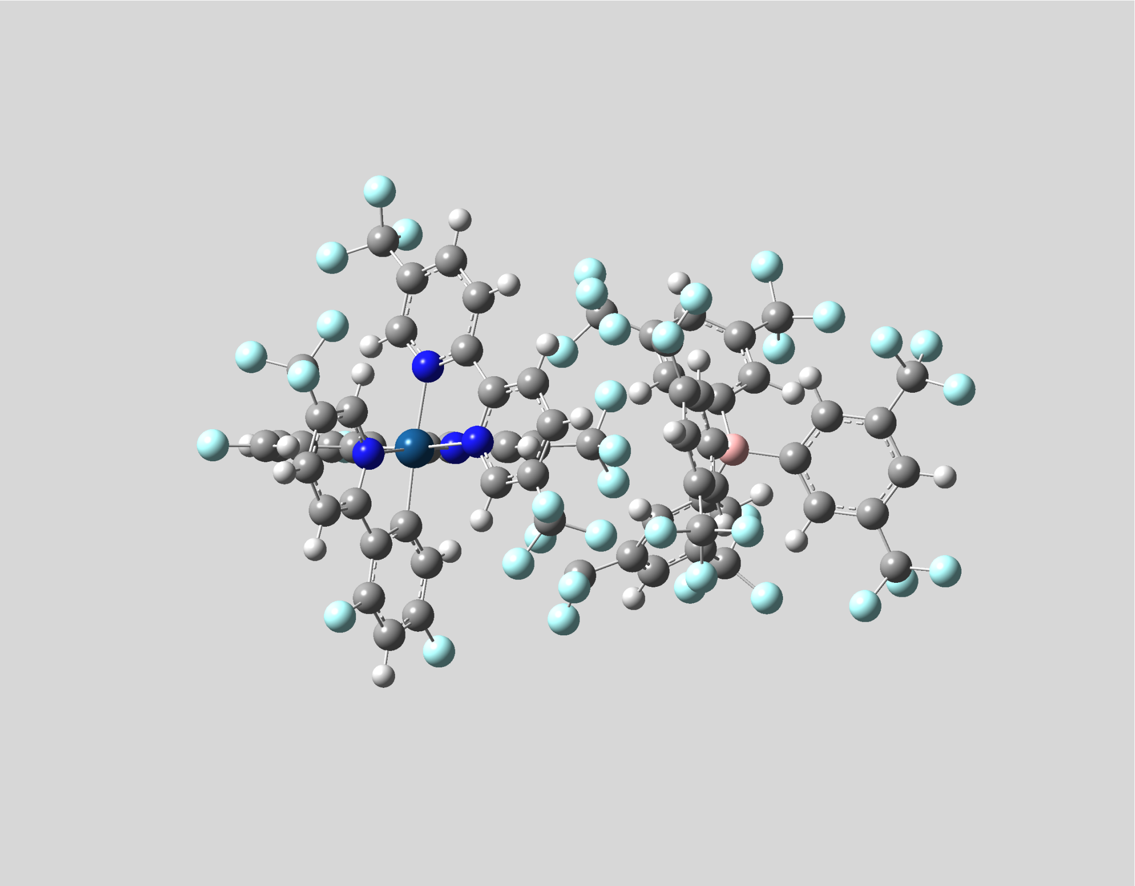

Figure S57: [Ir(III)(dFCF$_3$ppy)$_2$-(5,5'-dCF$_3$bpy)]$^+$ (abbv. [Ir(dCF$_3$)]$^+$) paired with tetrakis[3,5-bis(trifluoromethyl)phenyl]borate (abbv. $[BArF_4]^-$) optimized geometry

XYZ Coordinates of optimized structure of [Ir(III)(dFCF$_3$ppy)$_2$-(5,5'-dCF$_3$bpy)]$^+$ (abbv. [Ir(dCF$_3$)]$^+$) paired with tetrakis[3,5-bis(trifluoromethyl)phenyl]borate (abbv. $[BArF_4]^-$) :

Element	x	y	z
Ir	−5.03685	−0.01133	−0.00029
C	−8.04045	−0.42857	−0.11907
C	−9.28638	−1.05134	−0.31117
C	−9.33797	−2.41342	−0.64407
C	−8.13117	−3.12043	−0.76858
C	−6.91376	−2.45689	−0.56029
N	−6.86901	−1.14151	−0.24267
H	−10.29408	−2.90161	−0.79490
H	−10.21027	−0.49706	−0.20745
H	−5.96606	−2.97493	−0.63358
C	−6.35867	2.76089	0.62325
C	−7.40910	3.65720	0.86727
C	−8.73702	3.20735	0.78314
C	−8.97217	1.86549	0.45288
C	−7.88571	1.00434	0.22199
H	−9.56929	3.87932	0.96299
H	−5.32360	3.07543	0.66637
H	−9.98926	1.50594	0.38234
C	−7.09560	5.07539	1.25283
C	−8.12027	−4.57044	−1.16435
F	−8.11192	5.96177	0.88902
F	−5.91099	5.53612	0.66155
F	−6.91931	5.22222	2.64269
F	−9.28215	−5.23835	−0.77764
F	−8.01048	−4.73612	−2.55978
F	−7.03560	−5.26282	−0.60701
N	−6.59109	1.46454	0.30678
C	−3.88905	0.54247	2.66717
C	−3.73044	0.47835	4.07113
C	−4.58440	−0.31546	4.83982
C	−5.60042	−1.04873	4.19494
C	−5.72832	−0.96105	2.80749
N	−4.90005	−0.18878	2.06032
H	−4.45757	−0.36814	5.91636
H	−2.94218	1.04550	4.54381
H	−6.48830	−1.52171	2.28197
C	−2.70790	1.91933	−0.61743
C	−1.64327	2.72377	−0.20034
C	−1.25059	2.86748	1.13547
C	−1.99200	2.14973	2.06838
C	−3.07991	1.31812	1.72807
H	−0.42049	3.49932	1.42835
H	−2.93369	1.86644	−1.67623
C	−6.55112	−1.89120	4.98944
F	−5.89257	−2.67409	5.94974
F	−7.48741	−1.11679	5.70334
F	−7.29526	−2.76674	4.18828
C	−3.44628	1.19939	0.34459
F	−0.92157	3.42816	−1.16876
C	−3.46347	−1.70787	−1.79791

Element	X	Y	Z
C	−2.57851	−2.73987	−2.17653
C	−1.95601	−3.59096	−1.26955
C	−2.25443	−3.37147	0.08044
C	−3.11787	−2.37032	0.53503
H	−1.28216	−4.37602	−1.59150
H	−3.28284	−2.27363	1.60191
C	−5.63618	1.06212	−2.78537
C	−5.56185	1.11437	−4.17924
C	−4.75289	0.18316	−4.85996
C	−4.04421	−0.76591	−4.11984
C	−4.14026	−0.78663	−2.70894
H	−4.68336	0.19404	−5.94276
H	−6.24763	1.76216	−2.23356
F	−1.60977	2.28631	3.41255
N	−4.95134	0.13868	−2.06622
C	−3.73839	−1.51739	−0.40113
C	−6.31016	2.17362	−4.92814
F	−7.39760	2.68035	−4.20004
F	−5.50563	3.28771	−5.24621
F	−6.81641	1.70651	−6.14819
F	−2.28825	−2.94890	−3.53438
F	−1.64679	−4.20538	1.02338
H	−3.41834	−1.48981	−4.62048
C	4.47147	−0.32431	−0.26093
C	3.91490	−0.09170	−1.54508
C	3.60246	−0.89762	0.69532
C	2.57597	−0.39816	−1.84673
H	4.54468	0.31359	−2.33380
C	2.26060	−1.20987	0.39189
H	3.97433	−1.12614	1.68985
C	1.72586	−0.95700	−0.87834
H	0.69610	−1.20313	−1.11210
C	6.47764	−0.13557	1.59819
C	7.58643	−0.85951	2.08968
C	5.77055	0.64140	2.55382
C	7.96136	−0.82144	3.45018
H	8.17670	−1.46503	1.40755
C	6.13814	0.67308	3.90983
H	4.91930	1.23655	2.23198
C	7.24352	−0.05834	4.37870
H	7.52604	−0.03781	5.42530
C	6.83784	−1.19905	−0.89623
C	6.73320	−2.56884	−0.53398
C	7.53409	−0.93042	−2.09507
C	7.30654	−3.59294	−1.30695
H	6.18883	−2.84061	0.36709
C	8.11127	−1.95891	−2.87174
H	7.62455	0.09432	−2.44408
C	8.00987	−3.30213	−2.48903
H	8.45671	−4.09098	−3.08402

Element	X	Y	Z
C	6.53098	1.47520	−0.43454
C	7.90745	1.81387	−0.45685
C	5.63115	2.53246	−0.70666
C	8.35294	3.11440	−0.75909
H	8.64505	1.05062	−0.21852
C	6.07711	3.83839	−0.99897
H	4.56207	2.34265	−0.68988
C	7.44325	4.14799	−1.03730
H	7.78398	5.15087	−1.26895
C	7.12824	−5.02428	−0.90131
C	8.87143	−1.58649	−4.10183
C	1.38947	−1.78932	1.46346
C	2.04264	−0.09076	−3.21169
C	9.16530	−1.59806	3.88286
C	5.38200	1.54519	4.86417
C	9.81790	3.37821	−0.81367
C	5.07088	4.92422	−1.23200
F	6.99020	−5.19341	0.48414
F	5.97736	−5.62162	−1.47019
F	8.20218	−5.84313	−1.29648
F	9.20320	−2.67060	−4.91933
F	8.20478	−0.65696	−4.91738
F	10.12187	−0.95281	−3.79978
F	10.36821	−1.10210	3.31916
F	9.11895	−2.95243	3.49283
F	9.36733	−1.59677	5.26905
F	5.41697	1.07469	6.18790
F	5.89290	2.86702	4.92636
F	4.02714	1.68870	4.52872
F	0.93945	−0.82120	2.39391
F	2.04782	−2.76239	2.23943
F	0.22850	−2.40087	0.96685
F	0.90326	−0.83974	−3.54452
F	2.98100	−0.32272	−4.23448
F	1.66030	1.26496	−3.36496
F	4.57580	5.48532	−0.03041
F	3.93602	4.48160	−1.92983
F	5.58962	6.00678	−1.96321
F	10.54760	2.78406	0.23020
F	10.16235	4.73771	−0.81071
F	10.43635	2.85764	−2.00400
B	6.07282	−0.04790	0.00302

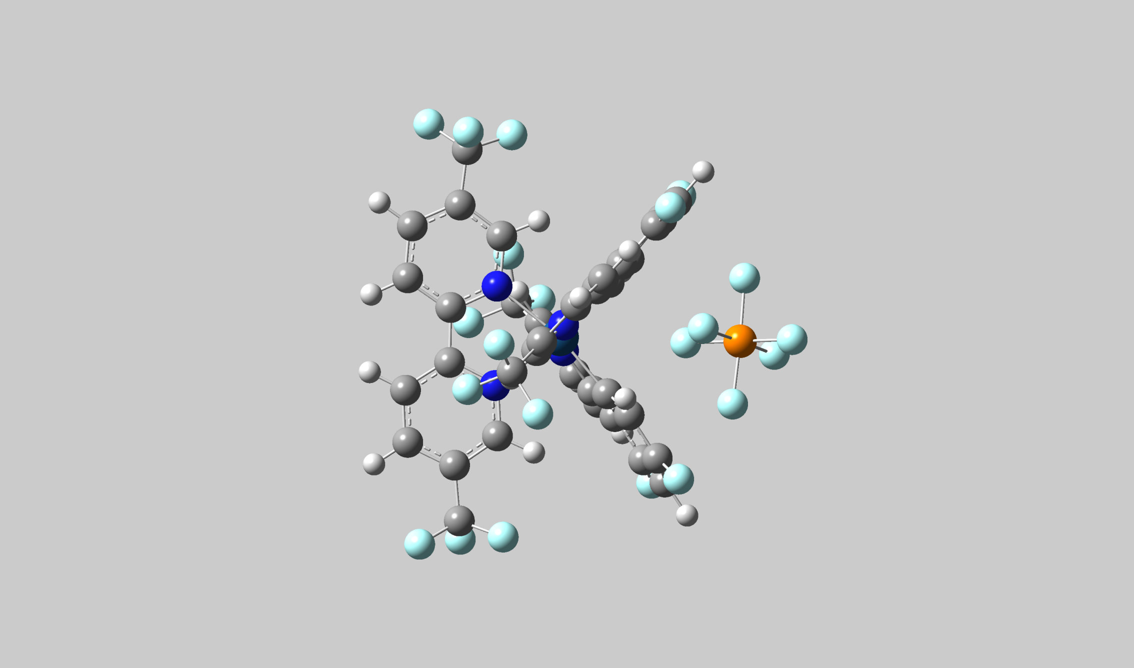

Figure S58: $[Ir(III)(dFCF_3ppy)_2$-(5,5'-$dCF_3bpy)]^+$ (abbv. $[Ir(dCF_3)]^+$) paired with hexafluorophosphate (abbv. $[PF_6]^-$) optimized geometry

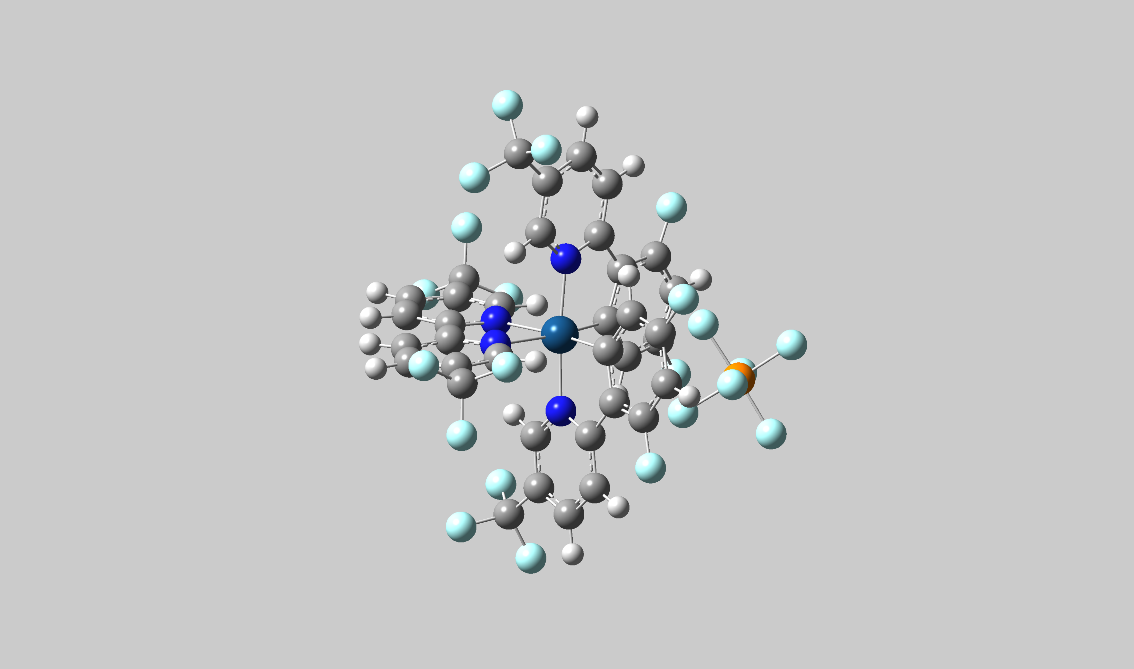

Figure S59: $[Ir(III)(dFCF_3ppy)_2$-(5,5'-$dCF_3bpy)]^+$ (abbv. $[Ir(dCF_3)]^+$) paired with hexafluorophosphate (abbv. $[PF_6]^-$) optimized geometry

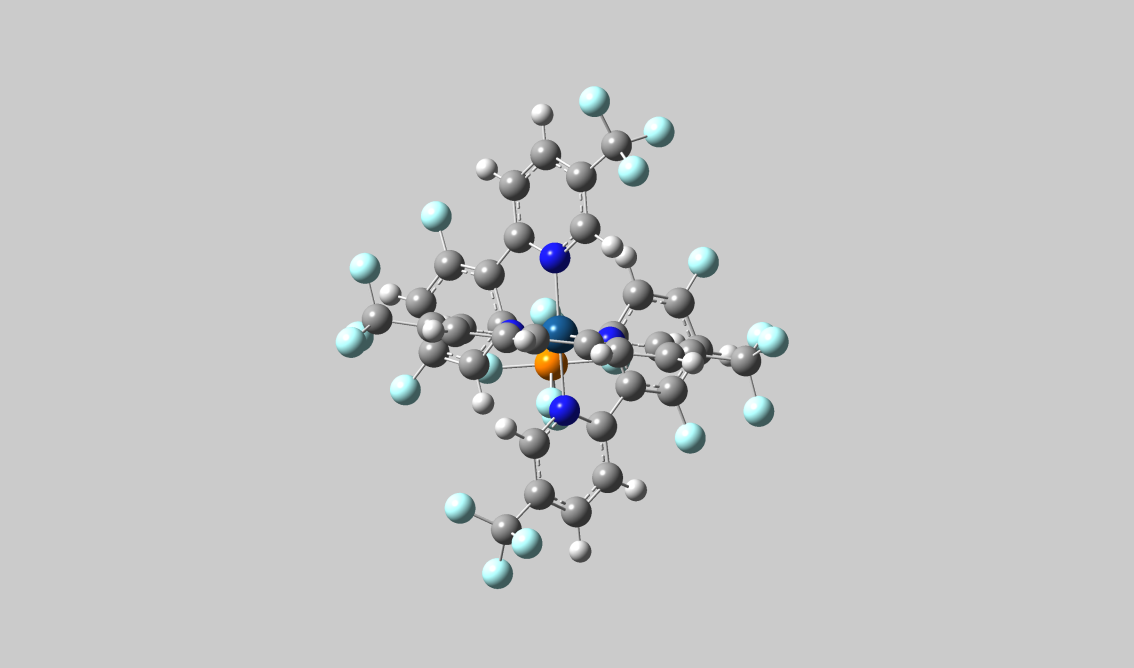

Figure S60: $[Ir(III)(dFCF_3ppy)_2\text{-}(5,5'\text{-}dCF_3bpy)]^+$ (abbv. $[Ir(dCF_3)]^+$) paired with hexafluorophosphate (abbv. $[PF_6]^-$) optimized geometry

XYZ Coordinates of optimized structure of bpy)]$^+$ (abbv. $[Ir(dCF_3)]^+$) paired with hexafluorophosphate (abbv. $[PF_6]^-$) :

Element	X	Y	Z
Ir	−0.08702	0.14199	0.02266
C	−1.82273	2.62230	0.28234
C	−2.86992	3.53642	0.06505
C	−3.91214	3.20638	−0.81341
C	−3.88116	1.95785	−1.45568
C	−2.81850	1.07813	−1.20329
N	−1.81250	1.40147	−0.35388
H	−4.72926	3.89886	−0.98760
H	−2.88094	4.49693	0.56494
H	−2.76824	0.10088	−1.66816
C	1.37076	2.06394	2.02745
C	1.53504	3.20326	2.82890
C	0.56073	4.21403	2.80103
C	−0.55493	4.04933	1.96719
C	−0.67759	2.88657	1.18492
H	0.67187	5.10734	3.40691
H	2.10789	1.27011	2.01138
H	−1.31441	4.82065	1.93817
C	2.73507	3.29973	3.73625
C	−4.94768	1.56429	−2.44574
F	3.01724	4.61761	4.08720
F	3.87811	2.75964	3.13862
F	2.53155	2.58851	4.93040
F	−6.14222	2.24451	−2.22051
F	−4.55225	1.85066	−3.76315
F	−5.21730	0.19352	−2.39738
N	0.29059	1.90849	1.22329
C	0.08059	−1.61446	2.39718
C	−0.34065	−2.28530	3.57083
C	−1.63618	−2.10226	4.06149
C	−2.51510	−1.24651	3.36810
C	−2.06806	−0.60835	2.20968
N	−0.80955	−0.77976	1.73362
H	−1.96196	−2.62071	4.95783
H	0.34974	−2.94129	4.08132
H	−2.72704	0.03374	1.64152
C	2.80081	−0.94519	−0.09510
C	3.83792	−1.73090	0.42403
C	3.71862	−2.50562	1.58439
C	2.48523	−2.46192	2.22508
C	1.39393	−1.69268	1.76359
H	4.53827	−3.10545	1.96245
H	2.98654	−0.38900	−1.00679
C	−3.89948	−0.96903	3.87642
F	−4.45371	−2.06309	4.53596
F	−3.91823	0.10476	4.79024
F	−4.77511	−0.60716	2.83978
C	1.55799	−0.91648	0.56639
F	5.05408	−1.74748	−0.24854
C	−0.15492	−1.19837	−2.58289

Element	X	Y	Z
C	−0.45572	−2.20474	−3.52755
C	−1.15176	−3.36729	−3.21489
C	−1.55454	−3.50423	−1.88079
C	−1.29117	−2.55127	−0.88845
H	−1.36824	−4.12229	−3.96166
H	−1.63415	−2.75308	0.11983
C	1.38622	2.03490	−1.85330
C	1.91485	2.48113	−3.06576
C	1.77087	1.67596	−4.21345
C	1.09969	0.45523	−4.10530
C	0.57321	0.03697	−2.85913
H	2.16764	2.00076	−5.17026
H	1.48529	2.62748	−0.95435
F	2.34852	−3.22836	3.38832
N	0.72997	0.85249	−1.74583
C	−0.58198	−1.38192	−1.22402
C	2.65328	3.78698	−3.11672
F	2.23320	4.66002	−2.09619
F	4.03842	3.62832	−2.95018
F	2.46859	4.44838	−4.33162
F	−0.04734	−2.04967	−4.85736
F	−2.25201	−4.65268	−1.52494
H	0.97626	−0.17946	−4.97103
P	1.56615	−3.22878	−0.78209
F	2.20008	−4.68253	−1.15846
F	0.06588	−3.85959	−0.87252
F	1.50115	−2.84721	−2.36547
F	0.93222	−1.77504	−0.40573
F	3.06642	−2.59798	−0.69167
F	1.63115	−3.61036	0.80128

1.7 Dielectric Loss

Ground-state measurement of the imaginary permittivity as a function of $[Ir(dCF_3)-PF_6]$ concentration ($[C_{PF6}]$) in hfb (black) with a linear fit of the form $\varepsilon'' = 4.47$e-21($[C_{PF6}]$) (dashed black line). Ground-state measurement of the imaginary permittivity as a function of $[Ir(dCF_3)-BAr_4^F]$concentration ($[C_{BArF}]$) in hfb (grey) with linear fit of the form $\varepsilon = 2.58$e-21 ($[C_{BArF}]$) (dashed grey line).All errors bars represent s.d. which includes systematic error introduced from our fitting procedure. Due to previous work showing the $[PF_6]^-$ counter-ion being tightly associated with the iridium complex in a low dielectric solvent,[5] it was asssumed that $[Ir(dCF_3)-PF_6]$ would also be a close contact ion-pair. Therefore, its calculated rotational time is 140 ps and its calculated electronic dipole moment magnitude is 13.9 [D]. Previous work shows $[BAr_4^F]^-$ to be not tightly associated with $[Ir(dCF_3)]^+$ allowing the cation to rotate freely, therefore, measure the intramolecular dipole moment of $[Ir(dCF_3)]^+$. The calculated rotational time of a freely rotating $[Ir(dCF_3)]^+$ is 37.8 ps and its calculated electronic dipole moment magnitude is 6.1 [D].

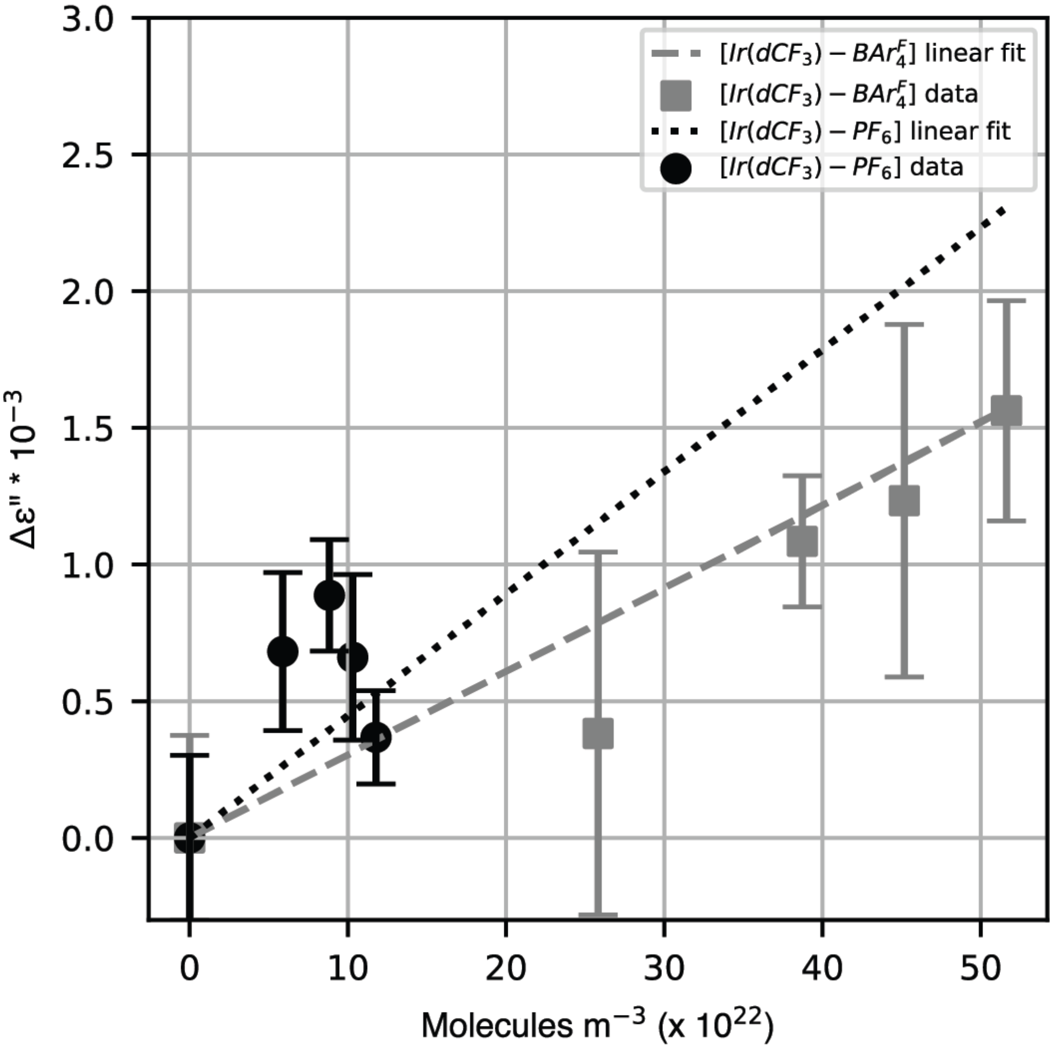

Figure S61: Ground-state measurement of the change in the imaginary permittivity as a function of ion-pair concentration. Both ion-pairs were dissolved in hfb.

Ground-state dielectric loss measurements were also performed on the solvent mixtures of fluorobenzene and 1,4-difluorobenzene at various volume ratios to obtain the real permittivity (ε'), or dielectric constant (ε_r), of each mixture at the GHz frequency range. The solvents were mixed at various volume ratios, where the notation 75 (dfb) / 25 (fbz) equal a solution containing 75% of dfb and 25% of fbz by volume). The real part of the permittivity of each solvent mixture was measured to be a linear combination of the volume and respective dielectric of each neat solvent at the GHz frequency range. Therefore, it is assumed that solvent mixtures of fluorobenzene and 1,4-difluorobenzene have dielectric constants at low frequencies to be a linear combination as well. The table below contains the measured ε' for each solvent mixture, the predicted ε' by taking a linear combination of the neat solvent components, and the predicted value of ε' at low frequencies ($\varepsilon'(0)$) used in determining electrostatic corrections to ΔG_{PET} used in the manuscript.

Table S3: Summary of dielectric constants of fluorobenzene/1,4-difluorobenzene solvent mixtures measured by dielectric loss spectroscopy.

**Solvent**	**Measured ϵ'**	**Predicted ϵ'**	**Calculated ϵ'(0)**
1,4-difluorobenzene (dfb)	1.9431 ±0.0004	1.9431	2.26
87.5 (dfb) / 12.5 (fbz)	2.31 ±0.01	2.29	2.67
75 (dfb) / 25 (fbz)	2.665 ±0.005	2.645	3.08
50 (dfb) / 50 (fbz)	3.33 ±0.01	3.35	3.91
25 (dfb) / 75 (fbz)	4.00 ±0.02	4.04	4.73
fluorobenzene (fbz)	4.75 ±0.02	4.75	5.55

1.8 Time-Resolved Dielectric Loss (TRDL)

Time-Resolved Dielectric Loss (TRDL) was performed on $[Ir(dCF_3) - PF_6]$ in hfb to see if the ion-pair's overall dipole moment (μ) would be depleted upon photoexcitation suggesting a PET reaction occurring. This was not observed as the $\Delta\mu$ is determined to be 3.30 [D] for the fast-living species (Species A) and 1.83 [D] for the longer living species (Species B), which do not coincide with the magnitude nor sign of a depletion of the ground-state dipole moment of the ion-pair of 13.9 [D]. Therefore, this data indicates no PET for $[Ir(dCF_3) - PF_6]$ when excited at 430 nm. Rather, the ion-pair maintains the same relative orientation compared to the ground-state, and the increase in the dipole moment upon photoexcitation is attributed to the formation of the MLCT state of $[Ir(dCF_3)]$. The increase in dipole moment does not indicate ion-pair reorganization upon photoexcitation as seen in similar systems, where the intramolecular dipole moment of the Ir-complex switches direction upon photoexcitation inducing the $[PF_6]^-$ counterion to reorganize to the lowest energy configuration and decreasing the overall dipole moment of the ion-pair.[5] The Ir-complex used in that study had tert-butyl groups attached to the bipyridine ligand, whereas the one within this study has CF_3 groups on the bipyridine ligand. The CF_3 groups have significantly more electron withdrawing character than the tert-butyl groups, leading to $[Ir(dCF_3)]^+$ having a ground-state dipole moment in the direction of the Ir-bipyridine bond with the partial positive charge on the Ir metal center and the partial negative charge residing on the bipyridine ligane. This coincides with DFT calculations of the Ir-complex described above. With this electron configuration of the Ir-complex in the ground-state, the $[PF_6]^-$ ion most likely resides closest to the partial positive charge on the Ir metal center and opposite the bipyridine ligand as shown in the structures above in Figures S58, S59, and S60. Upon excitation of the Ir-complex, these heteroleptic complexes form MLCT states with electron density residing on the bipyridine ligand.[5] An MLCT state with $[Ir(dCF_3) - PF_6]$ would increase the overall dipole moment of the ion-pair as the intramolecular dipole moment of $[Ir(dCF_3)]^+$ would increase due to the MLCT state and maintain a similar vector as the ground-state. Therefore, $[PF_6]^-$ ion already being in the lowest energy configuration and would not need to reorganize to achieve the minimum energy configuration of the system.

TRDL data illustrated in Figure S62 show the separated real (red) and imaginary (blue) permittivities with the global kinetic fits (black dashed traces) used to determine the trajectories of species A and B shown in Figure S63 previously described by Earley et al.[5] The positive change in the imaginary permittivity of species A and B demonstrates an increase in the excited-state dipole moment of $[Ir(dCF_3) - PF_6]$ in hfb compared to the ground-state, which demonstrates the persistence of the ion-pair instead of a PET between ions. The trajectories of species A and B shown in Figure S63 obtained by the global fit using Equations 6 and 7 .

$$\frac{d[A]}{dt} = g(t)N_0 - k_{AB}[A] - k_{AG}[A] \tag{6}$$

$$\frac{d[B]}{dt} = k_{AB}[A] - k_{BG}[B] \tag{7}$$

where [A] is the concentration of species A, [B] is the concentration of species B, $g(t)$ is a Gaussian profile modeling the laser pulse, N_0 is the concentration of excited

states of the solute, k_{AB} is the rate of conversion between species A and B, k_{AG} is the rate of conversion between species A and the ground-state, k_{BG} is the rate of conversion between species B and the ground-state. The values for the rate constants and the changes in permittivity are listed in the table below.

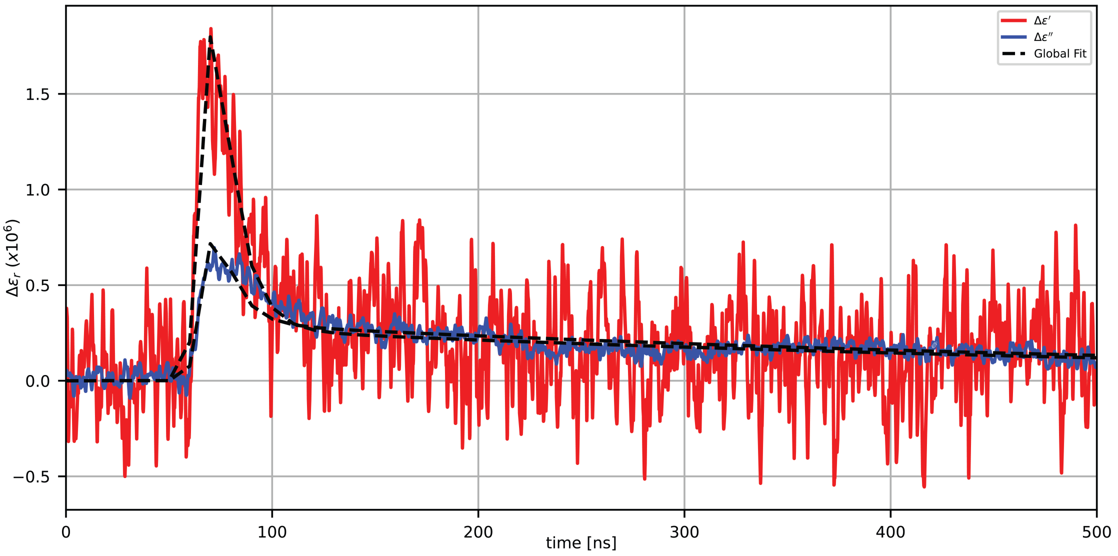

Figure S62: TRDL transients for $[Ir(dCF_3) - PF_6]$ in hfb with a concentration of 0.10 mM excited at 430 nm with an average pulse power of 1.82 mJ showing the real (red) and imaginary (blue) components of the signal. The black dashed lines are global fitting results using the kinetic model defined by Equations 6 and 7

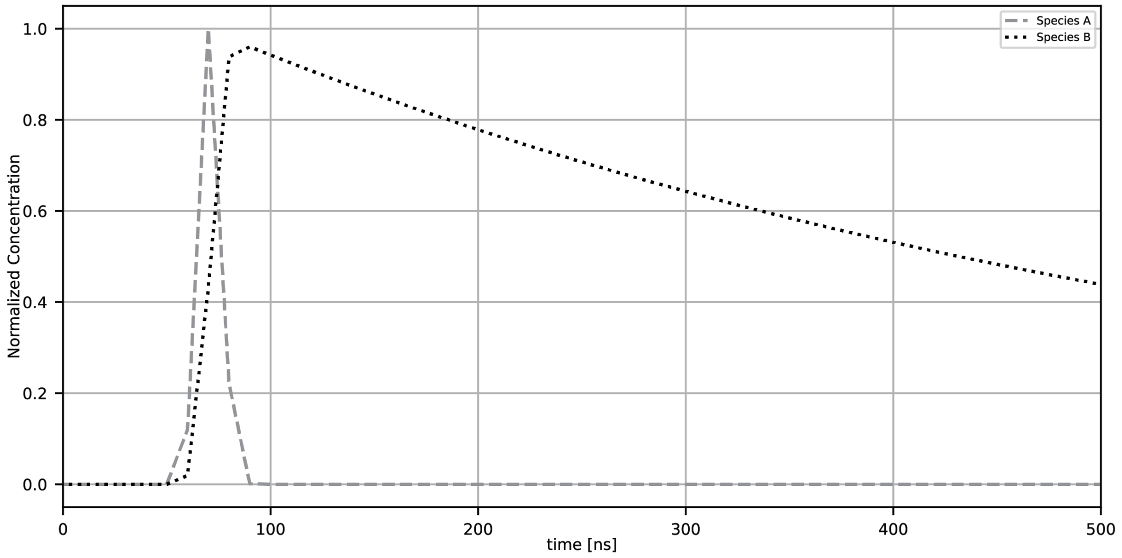

Figure S63: The black dashed traces show the species-associated kinetic trajectories that result from fitting our sequential two species model. Trajectories are normalized to show ratio between populations.

Table S4: Summary of the $[Ir(dCF_3)-PF_6]$ kinetic fit parameters and excited-state changes in electronic dipole moment and polarizability volume.

**Species**	ε_r **per molecule** $[10^{-27}]$	$\Delta\mu$ [**D**]	V_p [$\AA^3$]	**Rate constant** $[s^{-1}]$
A	$\Delta(149.9 - 54.8i)$	3.30 ± 0.5	677	7.48 X 10^7 (A → B)
B	$\Delta(15.4 - 17.0i)$	1.83 ± 0.1	70.2	1.91 X 10^6 (B → GS)

1.9 Ultrafast Transient Absorption

Ultrafast transient absorption (UTA) is shown of $[Ir(dCF_3) - PF_6]$ in hfb and acn excited at 400 nm. A UTA setup from Ultrafast Systems consisting of an Astrella Ti:Sapphire 1 kHz pump laser and an EOS probe laser was used. The pump pulse was routed through a TOPAS OPA to adjust the wavelength. Its pulse width was 100 fs and pulse energy 170 nJ. The probe pulse width was approximately 100 ps, though the overall instrumental IRF was roughly 1 ns. There is no long-lived absorption feature past 100 ns detectable by this intrumentation setup indicating the $[Ir(dCF_3) - PF_6]$ ion-pair does not produce long-lived neutral product species in low dielectric solvents.

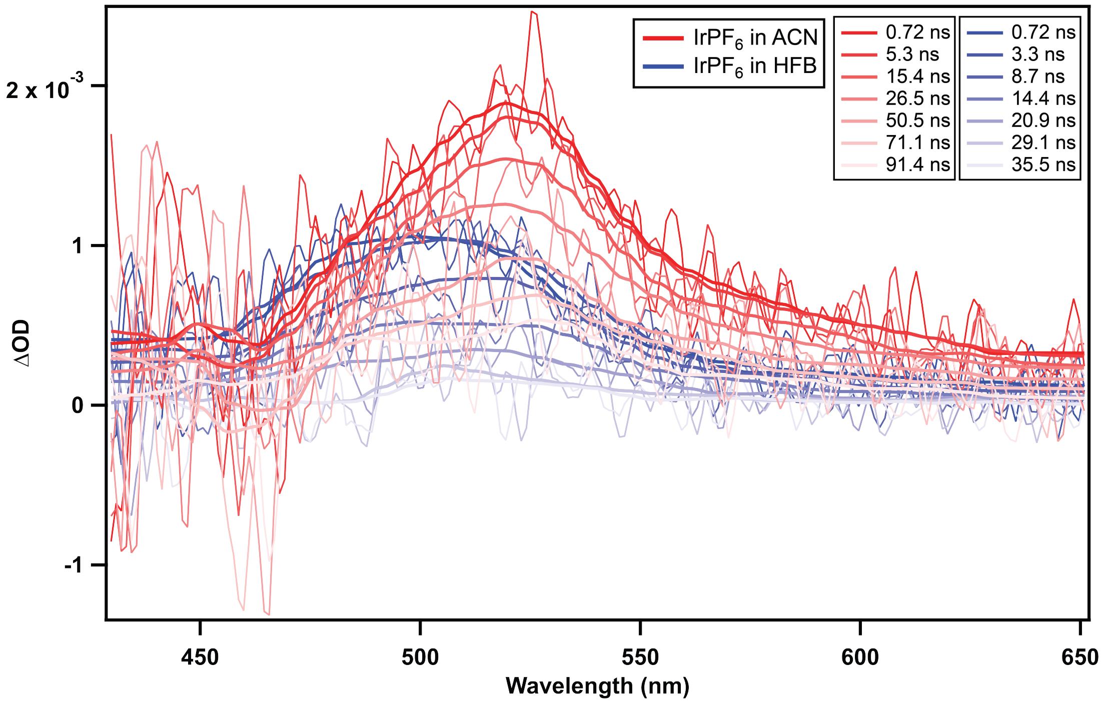

Figure S64: UTA spectra of $[Ir(dCF_3) - PF_6]$ in hfb and acn at a range of delay times after the excitation.

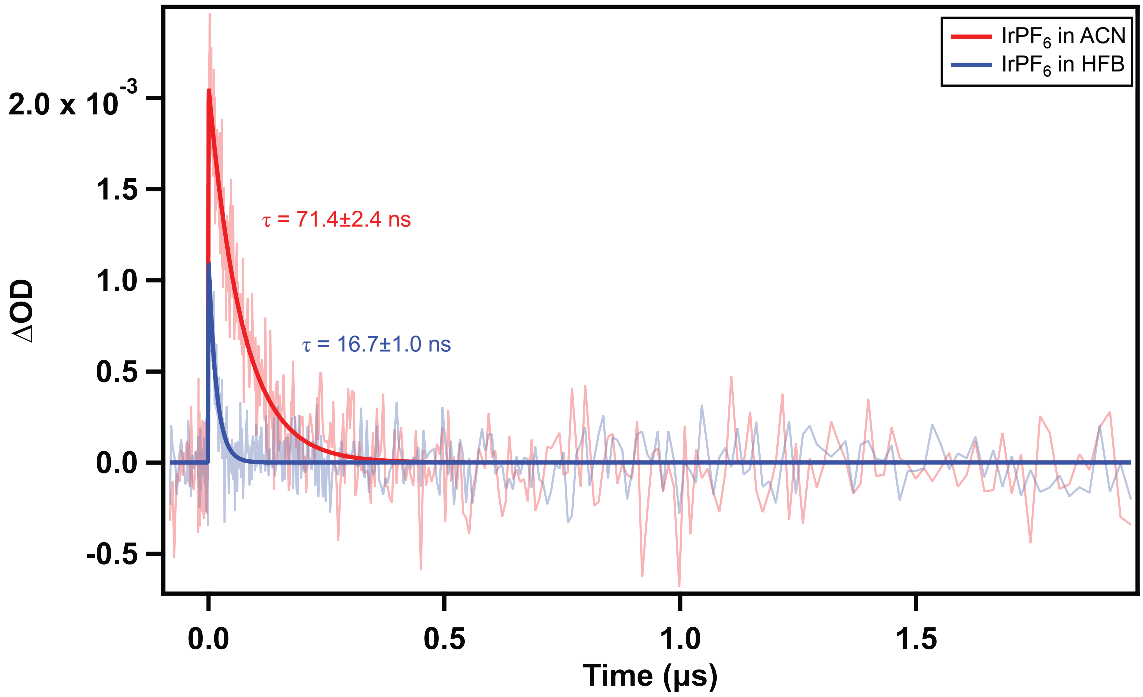

Figure S65: Kinetic traces of $[Ir(dCF_3) - PF_6]$ in hfb and acn monitored at 500 nm and 530 nm, respectively.

2 Oxidizing Solvents

The diagnostic UV/vis peak of $[Ir(dCF_3^{\cdot-})]^0$ centered around 530 nm appeared in two solvents, *N*,*N*-Dimethylformamide (DMF) and pyridine, whose calculated value of ΔG_{PET} between the ions in the ion-pair of $[Ir(dCF_3) - PF_6]$ was positive. Therefore, both of these solvent systems ought to be treated as outliers in the dataset demonstrating the PET within an ion-pair system becomes exergonic at a sufficiently low ε_r due to electrostatic work. These outliers in the data set can be explained via a PET between a photoexcited $[Ir(dCF_3)]^+$ and a molecule other than the counterion. In DMF, the solvent molecules have a sufficiently low oxidation potential (1.22 V vs $Fc^{0/+}$)[6, 7] for an photoexcited $[Ir(dCF_3)]^+$ (1.3 V vs $Fc^{0/+}$) to oxidize. Thus, the appearance of $[Ir(dCF_3^{\cdot-})]^0$ in the UV/vis spectra when $[Ir(dCF_3)-PF_6]$ is irradiated in DMF occurs due the PET between solvent DMF molecules and $[Ir(dCF_3)]^+$.

The electron source for the appearance of $[Ir(dCF_3^{\cdot-})]^0$ when $[Ir(dCF_3) - PF_6]$ is irradiated in pyridine is less clear. However, we are confident that the $[PF_6]^-$ counterion cannot be oxidized in pyridine due to the lack of reactivity observed in solvents with ε_r lower than pyridine ($\varepsilon_r = 12.5$), the reported larger oxidation potential of $[PF_6]^-$ compared to $[BAr_4^F]^-$, and the positive predicted ΔG_{PET} values for $[Ir(dCF_3)-PF_6]$ throughout the range of ε_r. Therefore, the observed production of $[Ir(dCF_3^{\cdot-})]^0$ must be forming due to a different mechanism than PET within the ion-pair and the evident reactivity in Figure S67 ought to be treated as an outlier of the dataset presented. Two competing explanations for the reported reactivity in pyridine are given below.

The first hypothesis is that the pyridine solvent used (Sigma Aldrich; Product Number: 5.89579) contains an impurity with an oxidation potential low enough to be oxidized by the excited state of $[Ir(dCF_3)]^+$ and with sufficient concentration to generate a significant amount of $[Ir(dCF_3^{\cdot-})]^0$. Previous reports indicate the presence of impurities in pyridine solvents and the necessary purification of pyridine solutions.[8]

The second hypothesis states a PET occurs between the chromophore within $[Ir(dCF_3)-PF_6]$ and a pyridine solvent molecule assisted by the energetic advantage of creating a more closely bound ion-pair in the product state, $[pyridine^+ - PF_6^-]$, compared to the reactant state, $[Ir(dCF_3) - PF_6]$. The testing of this hypothesis is out of the scope of this manuscript, however, we feel the arguments given below would be valuable to the photoredox community as many synthetic transformations utilize this framework of a neutral organic substrate being oxidized via the chromophore and creating a new ion-pair system between the organic substrate cation and the original counterion of the photocatalyst. The ΔG_{PET} for a PET between one of the ions within and ion-pair and a neutral substrate can be calculated using the Born correction term reported herein and a modified Coulomb correction term given below:

$$\Delta\mathcal{W} = \Big(\frac{q^2 n^2}{4\pi\varepsilon_0\varepsilon_r}\Big)\Big(\frac{1}{R_{DA}^R} - \frac{1}{R_{DA}^P}\Big) \tag{8}$$

where all of the constants are the came as the Coulombic correction of the manuscript, except R_{DA}^R and R_{DA}^P are the ion-pair center-to-center distances of the reactant and product states, respectively, assuming contact ion-pairing. The equation of $\Delta\mathcal{W}$ rep-

resents the difference in stabilization energy the association of ions provide to the system before and after the PET between the chromophore and a neutral organic solvent molecule. In the present case, $[pyridine^{+} - PF_6^{-}]$ can achieve a much smaller R_{DA} compared to $[Ir(dCF_3) - PF_6]$, thus providing additional driving force for the PET process to occur. When one takes the oxidation potential of pyridine to be 1.82 V vs $Fc^{0/+}$,[6, 7] the radius of pyridine to be 1.87$\mathring{A}$, $\varepsilon_r = 12.5$, $R_{DA}^{R} = 5.25\mathring{A}$, and $R_{DA}^{P} = 1.32\mathring{A}$, the ΔG_{PET} is calculated to be negative and the process is predicted to be exergonic.

The possibility for $R_{DA}^{P} = 1.32\mathring{A}$ to be a valid ion-pair distance is remarkably low due to repulsive forces from the nuclei restricting tighter ion-pairing making the first hypothesis much more likely to be valid. However, the calculations and explanation of the electrostatic contribution to a PET between an ion-pair and a neutral substrate provided within this section may provide inspiration for others to utilize the dielectric environment to perform novel chemistry.

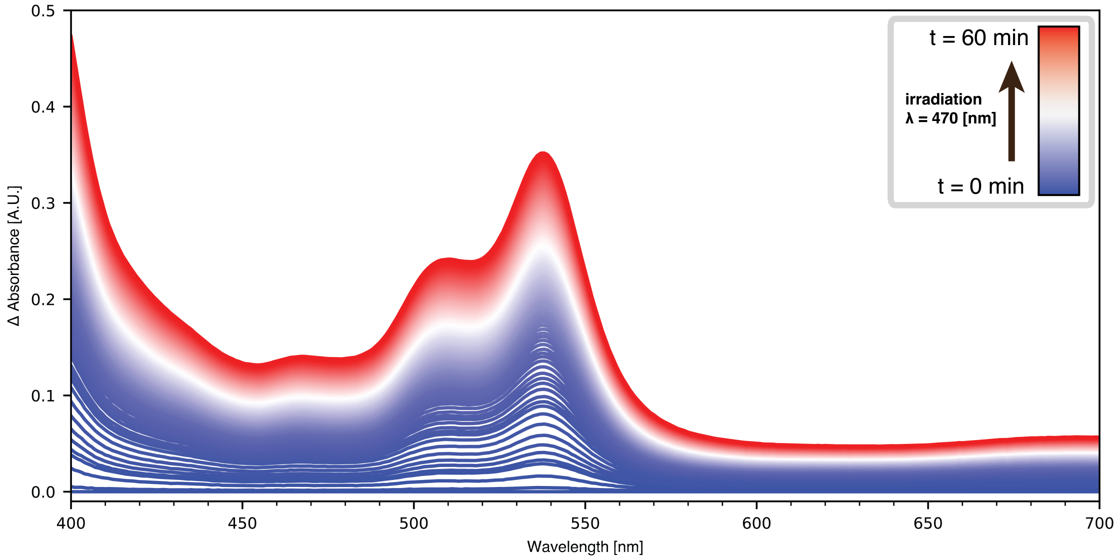

Figure S66: $[Ir(dCF_3) - PF_6]$ dissolved in *N*,*N*-Dimethylformamide (dmf) with a concentration of 0.10 mM and irradiated with 29.0 mW of 470 nm light in the photoreactor setup described above. A total of 120 spectra were collected every 30 seconds. The first absorption spectrum is subtracted from each spectrum resulting in the resulting difference spectra.

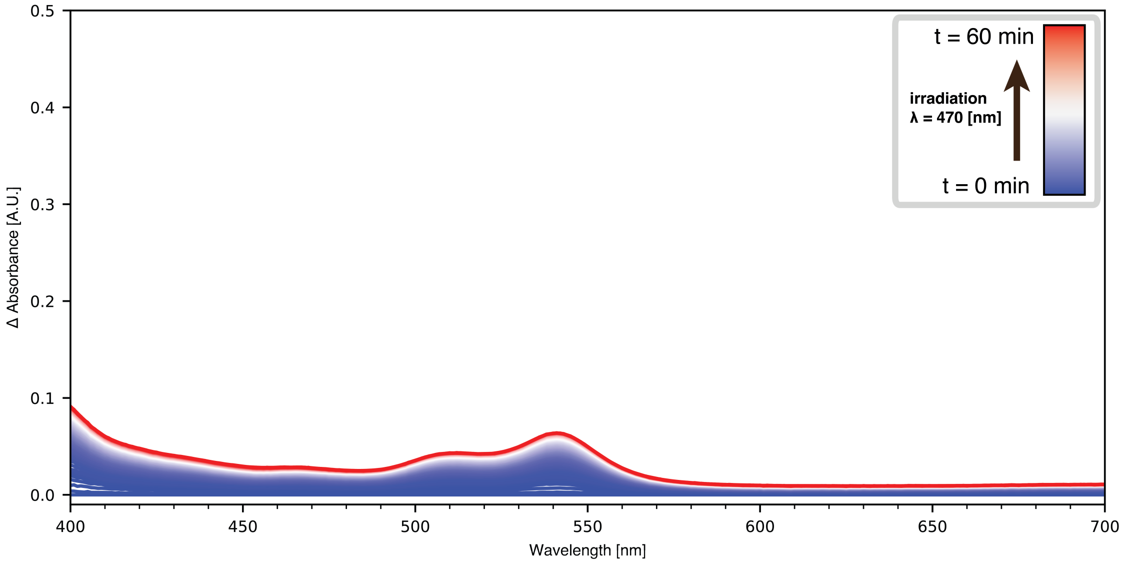

Figure S67: $[Ir(dCF_3) - PF_6]$ dissolved in pyridine (pyr) with a concentration of 0.10 mM and irradiated with 29.0 mW of 470 nm light in the photoreactor setup described above. A total of 120 spectra were collected every 30 seconds. The first absorption spectrum is subtracted from each spectrum resulting in the resulting difference spectra.

3 Correction term values in eV Tables

Table S5: Summary of the $[Ir(dCF_3) - BAr_4^F]$ Born and Columbic correction terms to the change in the Gibbs free energy in units of eV/mole and the measured composite rate constant for the formation of $[Ir(dCF_3^{\cdot -})]^0$ in each solvent condition in units of s^{-1}. The Gibbs free energy change without corrections ($\Delta G_{PET}^{Uncorrected}$) is provided as a reference. The constants used to calculate the correction terms and ΔG_{PET} as a function of ε_r are listed as follows: $F = 96485.3321\ C \cdot mol^{-1}$, $E_{1/2}^{ox}(D) = +1.52\ V$ vs $\text{Fc}^{0/+}$, $E_{1/2}^{red}(A) = -1.07\ V$ vs $\text{Fc}^{0/+}$, $\mathcal{E}_{0-0} = 2.37\ eV$, $q = 1.602176487\ e - 19\ C$, $n = 1$, $\varepsilon_0 = 8.854e - 12\ F \cdot m^{-1}$, $z_D = -1$, $z_A = +1$, $r_D = 5.58e - 10\ m$, $r_A = 5.16e - 10\ m$, $\varepsilon_D = \varepsilon_A = 37.5$, $R_{DA} = 8.25e - 10\ m$.

**Solvent**	ε_r	$\mathcal{W}$	ΔG_s	ΔG_{PET}	$\Delta G_{PET}^{Uncorrected}$	k_{rxn}
hexafluorobenzene	2.02	0.863	-1.258	-0.175	0.22	5.2 x10^3
1,4-difluorobenzene	2.26	0.772	-1.118	-0.126	0.22	1.7 x10^3
87.5 (dfb) / 12.5 (fbz)	2.67	0.653	-0.935	-0.0618	0.22	1.5 x10^3
75 (dfb) / 25 (fbz)	3.08	0.566	-0.801	-0.0148	0.22	5.6 x10^2
50 (dfb) / 50 (fbz)	3.91	0.446	-0.616	0.0500	0.22	2.5 x10^2
25 (dfb) / 75 (fbz)	4.73	0.369	-0.497	0.0917	0.22	6.2 x10^1
fluorobenzene	5.55	0.314	-0.413	0.121	0.22	3.5 x10^1
tetrahydrofuran	7.58	0.230	-0.284	0.167	0.22	4.8 x10^0
α, α, α-trifluorotoluene	9.40	0.191	-0.215	0.186	0.22	-1.6 x10^2
acetonitrile	37.5	0.047	0.00	0.266	0.22	-1.1 x10^1

Table S6: Summary of the $[Ir(dCF_3) - PF_6]$ Born and Columbic correction terms to the change in the Gibbs free energy in units of eV/mole and the measured composite rate constant for the formation of $[Ir(dCF_3^{\cdot -})]^0$ in each solvent condition in units of s^{-1}.The Gibbs free energy change without corrections ($\Delta G_{PET}^{Uncorrected}$) is provided as a reference. The constants used to calculate the correction terms and ΔG_{PET} as a function of ε_r are listed as follows: $F = 96485.3321\ C \cdot mol^{-1}$, $E_{1/2}^{ox}(D) = +2.626\ V$ vs $\text{Fc}^{0/+}$, $E_{1/2}^{red}(A) = -1.07\ V$ vs $\text{Fc}^{0/+}$, $\mathcal{E}_{0-0} = 2.37\ eV$, $q = 1.602176487\ e - 19\ C$, $n = 1$, $\varepsilon_0 = 8.854e - 12\ F \cdot m^{-1}$, $z_D = -1$, $z_A = +1$, $r_D = 1.71e - 10\ m$, $r_A = 5.16e - 10\ m$, $\varepsilon_D = \varepsilon_A = 37.5$, $R_{DA} = 5.25e - 10\ m$.

**Solvent**	ε_r	$\mathcal{W}$	ΔG_s	ΔG_{PET}	$\Delta G_{PET}^{Uncorrected}$	k_{rxn}
hexafluorobenzene	2.02	1.36	-2.63	0.056	1.33	-4.2 x10^1
1,4-difluorobenzene	2.26	1.21	-2.33	0.206	1.33	2.6 x10^1
87.5 (dfb) / 12.5 (fbz)	2.67	1.03	-1.95	0.401	1.33	-2.9 x10^0
75 (dfb) / 25 (fbz)	3.08	0.890	-1.67	0.544	1.33	-1.4 x10^1
50 (dfb) / 50 (fbz)	3.91	0.701	-1.29	0.741	1.33	-1.4 x10^1
25 (dfb) / 75 (fbz)	4.73	0.579	-1.04	0.868	1.33	5.9 x10^0
fluorobenzene	5.55	0.494	-0.862	0.957	1.33	-9.3 x10^0
tetrahydrofuran	7.58	0.362	-0.592	1.10	1.33	7.9 x10^0
α, α, α-trifluorotoluene	9.40	0.292	-0.449	1.16	1.33	-4.0 x10^1
acetonitrile	37.5	0.073	0.00	1.40	1.33	9.7 x10^1

References

(1) Stoll, S.; Schweiger, A. *Journal of magnetic resonance* **2006**, *178*, 42–55.

(2) Baek, Y.; Reinhold, A.; Tian, L.; Jeffrey, P. D.; Scholes, G. D.; Knowles, R. R. *Journal of the American Chemical Society* **2023**.

(3) Frisch, M. J. et al. Gaussian˜16 Revision C.01, Gaussian Inc. Wallingford CT, 2016.

(4) Sperger, T.; Sanhueza, I. A.; Kalvet, I.; Schoenebeck, F. *Chemical Reviews* **2015**, *115*, 9532–9586.

(5) Earley, J. D.; Zieleniewska, A.; Ripberger, H. H.; Shin, N. Y.; Lazorski, M. S.; Mast, Z. J.; Sayre, H. J.; McCusker, J. K.; Scholes, G. D.; Knowles, R. R.; Reid, O. G.; Rumbles, G. *Nature Chemistry* **2022**, *14*, Publisher: Nature Research, 746–753.

(6) Fuchigami, T.; Atobe, M.; Inagi, S., *Fundamentals and applications of organic electrochemistry: synthesis, materials, devices*; John Wiley & Sons: 2014.

(7) Pavlishchuk, V. V.; Addison, A. W. *Inorganica Chimica Acta* **2000**, *298*, 97–102.

(8) Lindauer, R.; Mukherjee, L. *Pure and Applied Chemistry* **1971**, *27*, 265–272.
